# Effect of disability, homelessness, and neighborhood marginalization on risk adjustment for hospital performance measurement

**DOI:** 10.1093/aje/kwae401

**Published:** 2024-10-16

**Authors:** Surain B Roberts, Michael Colacci, Jiamin Shi, Hilary K Brown, Mahliqa Ashraf, Therese Stukel, Fahad Razak, Amol A Verma

**Affiliations:** Li Ka Shing Knowledge Institute, St Michael’s Hospital, Toronto, Ontario, Canada; Institute of Health Policy, Management and Evaluation, University of Toronto, Toronto, Ontario, Canada; Institute of Health Policy, Management and Evaluation, University of Toronto, Toronto, Ontario, Canada; Department of Medicine, University of Toronto, Toronto, Ontario, Canada; Li Ka Shing Knowledge Institute, St Michael’s Hospital, Toronto, Ontario, Canada; Department of Health & Society, University of Toronto Scarborough, Toronto, Ontario, Canada; Dalla Lana School of Public Health, University of Toronto, Toronto, Ontario, Canada; Institute of Health Policy, Management and Evaluation, University of Toronto, Toronto, Ontario, Canada; Institute of Health Policy, Management and Evaluation, University of Toronto, Toronto, Ontario, Canada; Institute for Clinical Evaluative Sciences, Toronto, Ontario, Canada; Li Ka Shing Knowledge Institute, St Michael’s Hospital, Toronto, Ontario, Canada; Institute of Health Policy, Management and Evaluation, University of Toronto, Toronto, Ontario, Canada; Department of Medicine, University of Toronto, Toronto, Ontario, Canada; Li Ka Shing Knowledge Institute, St Michael’s Hospital, Toronto, Ontario, Canada; Institute of Health Policy, Management and Evaluation, University of Toronto, Toronto, Ontario, Canada; Department of Medicine, University of Toronto, Toronto, Ontario, Canada; Department of Laboratory Medicine and Pathobiology, University of Toronto, Toronto, Ontario, Canada

**Keywords:** hospital performance measurement, risk adjustment, health equity, disability, homelessness, indices of neighborhood marginalization, in-hospital mortality, readmission

## Abstract

It is not known how disability, homelessness, or neighborhood marginalization influence risk-adjusted hospital performance measurement in a universal health care system. In this study, we evaluated the effect of including these equity-related factors in risk-adjustment models for in-hospital mortality, and 7- and 30-day readmission in 28 hospitals in Ontario, Canada. We compared risk adjustment with commonly used clinical factors to models that also included homelessness, disability, and neighborhood indices of marginalization. We evaluated models using historical data using internal-external cross-validation. We calculated risk-standardized outcome rates for each hospital in a recent reporting period using mixed-effects logistic regression. The cohort included 544 805 admissions. Adjustment for disability, homelessness, and neighborhood marginalization had little impact on discrimination or calibration of risk-adjustment models. However, the adjustment influenced comparative hospital performance on risk-standardized 30-day readmission rates, resulting in 5 hospitals being reclassified among below-average, average, and above-average groups. No hospital was reclassified for mortality and 7-day readmission. In a system with universally insured hospital services, adjustment for disability, homelessness, and neighborhood marginalization influenced estimates of hospital performance for 30-day readmission but not 7-day readmission or in-hospital mortality. These findings can inform researchers and policymakers as they consider when to adjust for these factors in hospital performance measurement.

## Introduction

Equity-related factors, such as disability, homelessness, and neighborhood marginalization, influence patient care and clinical outcomes.^1–5^ Whether, and how, to account for these factors when comparing the performance of different health care institutions is an important area of research.^6^ Risk-standardized measures of hospital performance, such as mortality and readmission rates, facilitate benchmarking across institutions with different patient populations, which can inform quality improvement and may affect allocation of funding.^7^^,^^8^ Recent analyses have demonstrated that Medicare’s new health equity adjustment will significantly reclassify hospital performance status and redistribute payments to hospitals caring for a greater proportion of minoritized and low-income populations.^9^ Some have argued that it may be harmful to include equity-related factors in risk adjustment, because this could potentially mask inequitable care provision.^6^ Others have suggested that omission of this information may perpetuate inequity by penalizing health care institutions that serve patients with higher complexity that is not accounted for in current risk adjustment methods.^10^ This ethical debate can be informed by an empirical understanding of how risk adjustment for equity-related factors influences estimates of hospital performance.

The reported importance of equity-related factors in predicting in-hospital outcomes varies. Several studies have identified associations between clinical outcomes, like hospital mortality and readmission, and individual-level factors like disability, homelessness, income and health insurance status, as well as neighborhood-level poverty, housing status, and education.^1^^,^^2^^,^^4^^,^^5^^,^^11–13^ Most of these studies have evaluated a single equity-related factor in the context of patients with a single disease category, with limited use of detailed clinical data (eg, laboratory test results) in risk adjustment. In addition, the majority of these studies have been conducted in the United States. Thus, there remain important gaps in understanding how equity-related factors influence risk adjustment across heterogeneous patient populations with high-quality clinical risk adjustment and in understanding whether the observed relationships are consistent in different health care systems.

The objective of this study was to evaluate the impact of including disability, homelessness, and neighborhood marginalization in risk-adjustment procedures for benchmarking hospital mortality and readmission rates in Ontario, Canada. These factors were selected because they can be measured with established methods in Ontario and are known to be associated with clinical outcomes.^3^^,^^14–22^ We investigated whether the inclusion of disability, homelessness, and neighborhood marginalization improved the predictive performance of risk-adjustment models and whether this influenced risk-standardized estimates across hospitals. We refer to disability, homelessness, and neighborhood marginalization as “equity-related factors” for brevity throughout this article, with full acknowledgement that they do not reflect all relevant axes of equity.

## Methods

### Data source

We conducted a retrospective cohort study using data from 28 hospitals (*n* = 12 academic and 16 community hospitals) in Ontario, Canada, that are part of the GEMINI hospital research network (representing approximately 50%-60% of inpatient beds in the province).^20^^,^^21^ GEMINI collects administrative and clinical data from hospital information systems with 98%-100% accuracy of selected data elements compared with manual chart review.^22^

We used the Ontario Marginalization Index (ON-Marg)^23^ as a well-established measure of neighborhood-level marginalization.^24–27^ The ON-Marg combines a wide range of sociodemographic indicators into 4 distinct dimensions of marginalization using data from Statistics Canada’s 2016 census. We linked these data to GEMINI admissions at the dissemination-area-level using Postal Code Conversion File Plus postal code linkage.^28^^,^^29^

### Study population

We included adults aged 18 years or older who were admitted to or discharged from a general medicine inpatient service. General medicine is a useful cohort for this study because it is the largest patient population cared for by a single service unit within hospitals (accounting for approximately 40% of all emergency admissions at study hospitals)^20^ and it includes a heterogeneous population with numerous conditions of widely varying severity,^30^ making risk adjustment essential for interfacility comparisons.^30^ In keeping with established Canadian risk adjustment methods,^31^ we excluded patients with a length of stay of longer than 1 year and age older 120 years, with palliative care as the most responsible diagnosis (*International Classification of Diseases, 10th Revision, Canada* [ICD-10-CA] code Z51.5), or with medical assistance in dying. Additionally, we excluded patients without a recorded census dissemination area, because their data could not be linked to neighborhood exposures.

Patient data were linked across hospitalizations using provincial health card numbers. Patients without a valid health card number were excluded from readmission analyses because readmission status cannot be determined without the health card number. These hospitalizations were not excluded from in-hospital mortality analyses. This is consistent with established Canadian risk adjustment methods.^31^^,^^32^

Data were divided into “training” and “reporting” periods based on discharge date. The training period was from May 1, 2018, through April 30, 2021, and the reporting period was from May 1, 2021, through April 20, 2022. These dates were selected to maximize the number of hospitals with complete data in the reporting period. All fitting and evaluation of risk adjustment models were done in the training data (including internal-external cross validation), and all hospital comparisons were performed in the reporting data.

### Inpatient risk adjustment methodology

Our base risk-adjustment models align with the high-quality clinical risk-adjustment methods developed by Kaiser Permanente and are as reported in our previous internal-external validation study.^33–35^ Risk factors included age, sex, admission urgency (elective or urgent), chronic comorbidities as measured by the Charlson Comorbidity Index, severity of acute illness as measured by the modified Laboratory-Based Acute Physiology Score (mLAPS), and restricted 2-way interactions between age, Charlson score, and mLAPS.^33–35^ Models for 7-day and 30-day readmission also included the number of discharges in the past 6 months (0, 1, ≥2). Only preadmission values were included in risk-adjustment models. Age was fit with a 4-knot restricted cubic spline.

Hospitalizations were grouped by most responsible discharge diagnosis, defined by the ICD-10-CA codes, which were further grouped into clinically relevant categories using the Clinical Classification Software Refined.^36–39^ Separate logistic regression models were fit within each diagnosis group, allowing for diagnosis-specific intercepts and coefficients. Diagnosis groups with fewer than 200 events were grouped together, and divided into 3 “other” groups by tertiles of observed event rate in the training data. Models trained on a given diagnosis group apply predictions to patients from that diagnosis group in the reporting period. An L2 penalty was applied to each model based on the maximum corrected Akaike information criterion using penalized maximum likelihood estimation.^40^

Canadian hospitals do not collect patient race, ethnicity, or language data in a standardized fashion.^40^ We included the following equity-related factors, which are associated with the clinical outcomes homelessness,^14^^,^^15^^,^^41^ disability,^3^^,^^16–18^ and 4 components of the ON-Marg^19^^,^^25^^,^^27^: neighborhood housing and dwellings, racialized and newcomer population, material resources, and age and labor force.^42^ The expanded models with equity-related factors included all of the same variables as the base models. Disability was a binary individual-level variable defined using established ICD-10 algorithms for physical, sensory, and cognitive disability.^3^^,^^43–51^ Homelessness was a binary individual-level variable defined based on modified criteria proposed by Richard et al. in Ontario, using a combination of no postal code, ICD-10 codes, and transfer to or from supportive housing.^52^

The ON-Marg dimensions are continuous neighborhood-level measures derived from principal component factor analysis of 42 a priori–selected neighborhood-level variables that capture theoretical aspects of different axes of marginalization.^42^^,^^53^^,^^54^ Higher values indicate greater marginalization.^23^ The 4 ON-Marg domains describe the following information: (1) households and dwellings: type and density of residential accommodations, proportion of residents living alone and proportion of rental dwellings; (2) material resources: attainment of basic material needs, proportion unemployed, proportion receiving governmental financial support, proportion considered low-income, and proportion without a high-school degree; (3) age and labor force: proportion of youth and senior dependents to adults aged 15-64 years, and proportion of adults not participating in labor force; and (4) racialized and newcomer population: proportion of population who immigrated in the past 5 years, and proportion of the population who self-identify as a visible minority. Details on the composition of each ON-Marg dimension are available in the ON-Marg User Guide.^55^

### Outcomes

We selected 3 outcomes that are commonly used in hospital performance measurement: in-hospital mortality, 7-day unplanned readmission, and 30-day unplanned readmission. In-hospital mortality was defined as death within the facility and was analyzed at the level of the hospitalization, with each admission treated as conditionally independent (consistent with established Canadian risk-adjustment methods).^31^ Readmission was defined as an admission to any medical or intensive care unit within 7 or 30 days of discharge. Our definition of readmission was based on the Canadian Institute for Health Information’s definition for national reporting of medical patients readmitted to hospital.^32^ To avoid counting interfacility transfers as unplanned readmissions, all contiguous inpatient hospitalizations within GEMINI were considered a single “episode of care,” and readmissions were defined only as occurring after the completion of the episode of care. Readmissions were attributed to the last hospital from which the patient was discharged before readmission. Consistent with Canadian Institute for Health Information approaches, the following episodes of care were excluded: episodes with discharge as death, episodes with at least 1 record for palliative care or mental health as most responsible discharge diagnosis, episodes in which a patient was transferred to and discharged from a hospital outside of the GEMINI network, and episodes where the last record was self sign-out. The following were not considered readmissions: episodes where the initial encounter was elective and episodes with at least 1 record for chemotherapy for neoplasm, palliative care, obstetric delivery, mental health, and medical assistance in dying.

GEMINI receives data after the inpatient has been discharged from hospital. If a patient is still hospitalized at the time of data extraction, that information will not be provided to GEMINI until the hospitalization has ended. This could result in an underestimation of readmission rates near the end of the study period because incomplete hospitalizations have not yet been included in GEMINI. To minimize this bias, 7-day readmission rates exclude the most recent 37 days collected from each hospital and 30-day readmission rates exclude the most recent 60 days.

### Evaluation of risk-adjustment models

Risk-adjustment models were evaluated using internal-external cross-validation.^56^ We removed a single hospital from the training data, fit risk-adjustment models on all other hospitals, and evaluated the performance on the specific held-out hospital. This procedure was repeated for each hospital. Evaluation of model performance considered smoothed calibration curves, area under the curve (AUC), Brier Skill Score [with the score based on the mortality rate of the held-out hospital as the reference: Brier Skill Score = 1−(Brier Skill Score/reference Brier Skill Score)], calibration slope, calibration intercept, Integrated Calibration Index, 95th percentile absolute vertical distance between the calibration curve and perfect calibration and discrimination slope.^57^^,^^58^ Predictions at each hospital were bootstrapped to calculate the SE of each performance metric at each hospital (*n* = 1000 iterations). We used random-effects meta-analysis to report pooled estimates of model performance that summarize metrics at each hospital.

### Risk-standardized hospital estimates

For each hospital in the reporting period, we calculated risk-standardized rates derived from the base models and from the expanded equity-related models. Our method of calculating risk-standardized rates aligned with method 4 discussed by Mohammed et al.^59^ The risk-standardized rate is the ratio of the predicted number of events at each hospital to the expected number of events given that hospital’s case mix, multiplied by the event rate across the cohort in the reporting period.^60^ The predicted and expected numbers of events were obtained from a mixed-effects logistic regression model based only on reporting period data. Probabilities from risk-adjustment models were treated as a fixed effect, and hospitals were treated as a random intercept. The predicted number of events is the sum of predicted probabilities including hospital random intercepts, and the expected number of events is the sum of predicted probabilities for a “typical” hospital.^59^^,^^61^ We report 95% coverage intervals calculated with cluster bootstrap methods in which hospitals were considered fixed entities and admissions were a random sample of admissions treated at that hospital. Within each hospital, we resampled admissions with replacement and held constant the sample size of each hospital at its observed value in the reporting period. This structure allows for resampling to capture that a patient who is admitted more often to a given hospital should be proportionally represented across bootstrap replications for that hospital. We computed the risk-standardized rate over 1000 iterations using this approach and reported the 2.5th and 97.5th percentiles. These 95% CIs were used to classify each hospital into below-average, average, or above average categories based on whether the upper or lower bound of the 95% CIs crossed the crude event rate.

We further sought to understand how the different equity-related factors might influence hospital risk-standardized hospital estimates. Specifically, we investigated how changes in hospital risk-standardized rates after adjustment for equity-related factors were associated with differences in the distribution of equity-related characteristics across hospitals. We fit univariable regression models with hospital as a categorical exposure and each equity-related variable as an outcome. We used logistic regression for binary variables and proportional odds regression for continuous variables,^62^ and reported log of the odds ratios for each hospital in a heat map for visual inspection. For each variable, the hospital whose median value (ie, proportion for binary variables) was ranked 12 of 23 in the reporting period was used as the reference.

All analyses were performed in R, version 4.1.0.^63^ The *rms* package^64^ was used for logistic regression, penalization, and proportional odds models. The *metafor* package was used for random-effects meta-analysis.^65^ The *lme4* package was used for mixed-effects logistic regression.^66^

## Results

The cohort included 569 288 admissions to 28 hospitals from May 2018 to April 2022. After excluding 2.9% of admissions without neighborhood-level data, the cohort included 552 960 admissions. All 28 hospitals were included in the training period (May 2018 to April 2021) and 23 hospitals were present in the reporting period (May 2021 to April 2022). Five hospitals’ data did not cover the full reporting period year and were excluded from the reporting period (*n* = 8155 hospitalizations), resulting in a final cohort of 544 805 hospitalizations. The cohorts for 30-day and 7-day readmission included 441 009 and 442 418 episodes of care, respectively. Missing provincial health card number resulted in the exclusion of 0.98% of admissions from readmission analyses. No other values were missing.

In the mortality cohort, median age at admission was 72.0 years (IQR = 58.0, 83.0), 49.7% were female patients, the median mLAPS score was 16 (IQR = 5, 30), 97.9% of admissions were urgent, 30.2% of patients had a Charlson Comorbidity Index score ≥ 2, the in-hospital mortality rate was 6.3%, the 30-day readmission rate was 13.1%, and the 7-day readmission rate was 4.5% (Table 1). No hospital contributed less than 1.4% or greater than 6.4% of admissions to the cohort. The top 5 most common diagnosis groups were heart failure (5.2%), pneumonia (3.9%), chronic obstructive pulmonary disease (3.5%), urinary tract infections (3.5%), and neurocognitive disorders (3.4%). The readmission cohorts are described in Table 1 and Table S1.

**Table 1:** Baseline characteristics of patients in each outcome cohort.

**Characteristic**	**In-hospital mortality** ^a^	**7-day readmission** ^a^	**30-day readmission** ^a^
No. of encounters	544 805	442 418	441 009
Age, median (Q1-Q3), years	72.0 (58.0-83.0)	72.0 (58.0-83.0)	72.0 (58.0-83.0)
Female sex	270 723 (49.7)	224 640 (50.8)	223 913 (50.8)
Admission category			
Elective	11 273 (2.1)	9095 (2.1)	9089 (2.1)
Urgent	533 532 (97.9)	433 323 (97.9)	431 920 (97.9)
mLAPS, median (Q1-Q3)	16.0 (5.00-30.0)	16.0 (5.00-29.0)	16.0 (5.00-29.0)
Charlson Comorbidity Score			
0	297 902 (51.3)	230 656 (52.1)	229 910 (52.1)
1	100 404 (18.4)	84 457 (19.1)	84 174 (19.1)
≥2	164 699 (30.2)	127 305 (28.8)	126 925 (28.8)
No. of discharges in the prior 6 months			
0	375 782 (69.0)	307 992 (69.6)	306 933 (69.6)
1	102 220 (18.8)	82 145 (18.6)	81 913 (18.6)
≥2	66 803 (12.3)	52 281 (11.8)	52 163 (11.8)
Equity-related factors			
Experiencing homelessness	10 906 (2.0)	8029 (1.8)	8018 (1.8)
Disability	75 496 (13.9)	60 664 (13.7)	60 527 (13.7)
Cognitive disability	8263 (1.5)	6331 (1.4)	6313 (1.4)
Physical disability	65 326 (12.0)	52 755 (11.9)	52 640 (11.9)
Sensory disability	4616 (0.8)	3698 (0.8)	3691 (0.8)
Housing and dwellings			
Most marginalized quintile	209 828 (38.5)	168 565 (38.1)	168 154 (38.1)
Least marginalized quintile	78 237 (14.3)	64 925 (14.7)	64 660 (14.7)
Racialized and newcomer population			
Most marginalized quintile	182 982 (33.6)	147 589 (33.3)	147 031 (33.3)
Least marginalized quintile	52 840 (9.7)	42 892 (9.7)	42 817 (9.7)
Material resources			
Most marginalized quintile	143 978 (26.4)	115 118 (26.0)	114 925 (26.0)
Least marginalized quintile	99 289 (18.2)	81 525 (18.4)	81 314 (18.4)
Age and labor force			
Most marginalized quintile	139 196 (25.5)	112 492 (25.4)	112 214 (25.4)
Least marginalized quintile	106 928 (19.6)	87 248 (19.7)	86 949 (19.7)
Outcome			
In-hospital deaths	34 516 (6.3)		
7-Day readmission		20 002 (4.5)	
30-Day readmission			57 875 (13.1)

Abbreviations: mLAPS, Modified Laboratory-based Acute Physiology Score; Q, quintile.

a Data are reported as no. (%) unless otherwise indicated.

Patients experiencing homelessness represented 2.0% of the cohort (*n* = 10 906), and patients with a disability represented 13.9% (1.5% cognitive, 12.0% physical, 0.8% sensory; *n* = 75 496) of the cohort (Table 1). The proportion of patients in the most and least marginalized quintiles for each ON-Marg domain are presented in Table 1.

### Performance of risk-adjustment models with and without equity-related factors

Performance of risk-adjustment models for in-hospital mortality was very similar across all metrics with and without equity-related factors (Table 2, Figure S2.1). Models for mortality, with and without equity-related factors, showed strong discrimination (pooled AUC = 0.84; 95% CI, 0.83-0.85 with and without equity-related adjustment) and were well calibrated along the range of predicted probabilities and hospitals (Figure S1.1).

**Table 2:** Pooled risk-adjustment model performance from internal-external cross-validation by hospital.^a^

**Parameter**	**Without adjustment for equity-related factors**	**With adjustment for equity-related factors**
Mortality		
AUC	0.84 (0.83-0.85)	0.84 (0.83-0.85)
Brier Skill Score	0.13 (0.12-0.14)	0.13 (0.12-0.14)
Calibration slope	1.00 (0.97-1.03)	0.99 (0.96-1.02)
Calibration intercept	−0.01 (−0.12 to 0.11)	−0.03 (−0.14 to 0.08)
Nagelkereke’s *R*^2^	0.25 (0.24-0.26)	0.25 (0.24-0.26)
Discrimination slope	0.137 (0.130-0.144)	0.138 (0.131-0.144)
Integrated Calibration Index	0.012 (0.010-0.014)	0.012 (0.009-0.014)
E95	0.043 (0.032-0.053)	0.041 (0.031-0.052)
7-day readmission		
AUC	0.64 (0.63-0.64)	0.64 (0.63-0.64)
Brier Skill Score	0.01 (0.01-0.01)	0.01 (0.01-0.01)
Calibration slope	0.94 (0.90-0.99)	0.92 (0.88-0.96)
Calibration intercept	−0.015 (−0.30 to −0.01)	−0.22 (−0.35 to −0.08)
Nagelkereke’s *R*^2^	0.03 (0.03-0.03)	0.03 (0.03-0.03)
Discrimination slope	0.012 (0.011-0.013)	0.013 (0.012-0.014)
Integrated Calibration Index	0.006 (0.005-0.007)	0.006 (0.005-0.007)
E95	0.013 (0.011-0.016)	0.012 (0.010-0.014)
30-day readmission		
AUC	0.68 (0.67-0.68)	0.68 (0.67-0.68)
Brier Skill Score	0.05 (0.04-0.05)	0.05 (0.04-0.05)
Calibration intercept	−0.06-(−0.13 to 0.02)	−0.08 (−0.15 to −0.01)
Calibration slope	0.97 (0.94-1.00)	0.95 (0.93-0.98)
Nagelkereke’s *R*^2^	0.08 (0.07-0.09)	0.08 (0.07-0.09)
Discrimination slope	0.053 (0.05-0.055)	0.053 (0.05-0.056)
Integrated Calibration Index	0.013 (0.01-0.016)	0.012 (0.009-0.014)
E95	0.029 (0.022-0.035)	0.028 (0.021-0.034)

Abbreviations: AUC, area under the curve; E95, 95th percentile absolute vertical distance between the calibration curve and line of perfect calibration.

a This table presents pooled estimates from the random-effects meta-analysis of internal-external cross-validation by hospital. 95% CIs are presented in parentheses. Forest plots are presented in Figures S2.1, S2.2, and S2.3.

**Figure 1:**
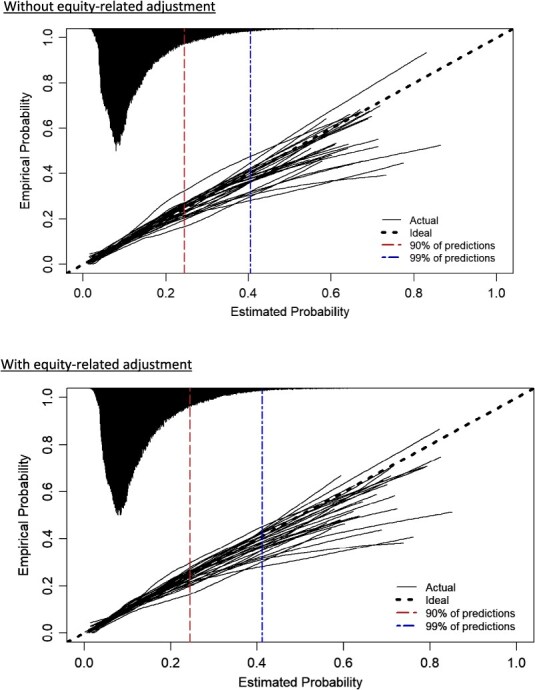
Calibration curves for 30-day readmission models with and without adjustment for equity-related factors. Each curve represents a held-out hospital during internal-external cross-validation. The histogram on the top axis represents the distribution of predicted probabilities and is not scaled to the *y*-axis. Calibration curves for in-hospital mortality and 7-day readmission are presented in Figures S1.1 and S1.2.

Performances of risk-adjustment models for 30-day and 7-day readmission were also similar with and without equity-related factors (Table 2, Figures S2.2 and S2.3). For 30-day readmission, models had moderate discrimination (pooled AUC =0.68; 95% CI, 0.67-0.68 with and without equity-related adjustment) and were well calibrated across the range of predicted probabilities and hospitals, though both tended to overestimate high risk in patients above the 90th percentile (Figure 1). Summary calibration metrics were similar when including the equity-related factors (pooled calibration intercept −0.06 [95% CI, −0.13 to 0.02] vs −0.08 [95% CI, −0.15 to −0.01]; pooled calibration slope 0.97 [95% CI, 0.94-1.00] vs 0.95 [95% CI, 0.93-0.98]). Discrimination was similar for 7-day readmission with and without equity-related factors (pooled AUC = 0.64; 95% CI, 0.63-0.64 for both), and models were reasonably calibrated at low and moderate values, though meaningful miscalibration was evident for high risk in patients above the 90th percentile at most hospitals (Figure S1.2). Models for 7-day readmission tended to overestimate the probability of 7-day readmission with and without equity-related adjustment.

### Hospital performance measurement

**Figure 2:**
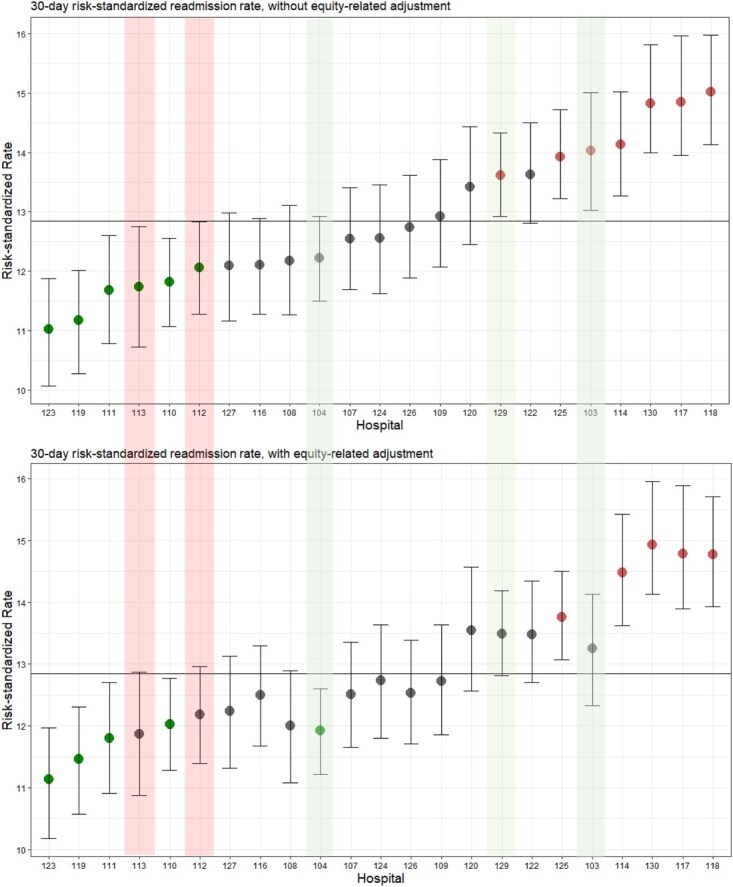
Caterpillar plots of risk-standardized 30-day hospital readmission rates with and without equity-related adjustment. The rank order of hospitals in the latter plot is maintained in the former to visualize changes in rank after equity-related adjustment. The horizontal line represents the crude 30-day readmission rate across all hospitals. Caterpillar plots for in-hospital mortality and 7-day readmission are presented in Figures S3.1 and S3.2. Green shaded bars denote hospitals that were reclassified downward and red shaded bars denote hospitals that were reclassified upward.

Risk-standardized 30-day readmission rate was the outcome most affected by adjustment for equity-related factors. We observed shrinkage to the mean after equity-related adjustment. Without equity-related adjustment, 6 hospitals were below average, 10 were average, and 7 were above average. After equity-related adjustment, 5 hospitals were reclassified: 2 hospitals moved from below average to average, 1 from average to below, and 2 from above average to average (Figure 2).

Classification of hospitals based on risk-standardized 7-day readmission rates was not affected by adjustment for equity-related factors. No hospitals were reclassified; in both cases, 5 were below average, 14 were average, and 4 were above average (Figure S3.2).

Risk-standardized mortality rates remained stable with or without adjustment for equity-related factors. No hospitals were reclassified after adjustment; in both cases, 7 hospitals were below average, 9 were average, and 7 were above average. (Figure S3.1).

Adjusting for equity-related factors tended to reduce risk-standardized rates for all outcomes across hospitals.

**Figure 3:**
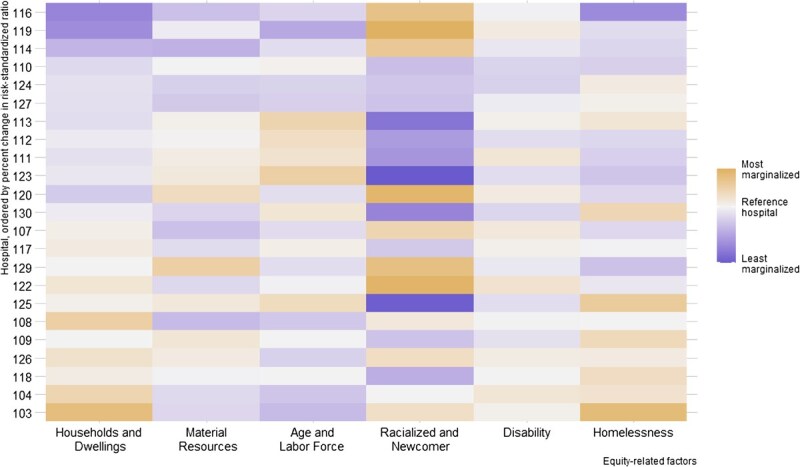
Heat map showing how differences in equity-related characteristics across hospitals were associated with changes in risk-standardized rates after adjustment for equity-related factors. Each row of the heat map corresponds to a hospital, and the hospitals are ordered by their relative change in risk-standardized rate after adjustment for equity-related factors. Thus, hospitals experiencing a greater increase after equity-related adjustment are at the top and hospitals experiencing a greater decrease are at the bottom of the heat map. Differences in equity-related characteristics across hospitals are depicted with color shading. Hospitals with lower marginalization are shaded in purple and higher marginalization in yellow. More extreme differences are shaded more darkly. For instance, darker cells in the racialized and newcomer column compared with the disability column indicate larger between-hospital difference in the former than the latter. The plot allows readers to visually inspect the association between the change in risk-standardized ratio and the marginalization of patients at a given hospital. Hospitals with the most marginalized patients in terms of households and homelessness have the largest relative decreases in risk-standardized rates, whereas for other factors, there is no apparent association between the change in risk-standardized rate and the degree of marginalization. To depict the shading for differences in equity-related factors, we fit univariable regression models with hospital as a categorical exposure and each equity-related variable as an outcome. We used logistic regression for binary variables and proportional odds regression for continuous variables^62^ and reported the log of odds ratios for each hospital in a heat map for visual inspection. The hospital whose median value (proportion for binary variables) was ranked 12th of 23 in the reporting period was used as the reference for that equity-related variable. These data are based on the reporting period only. Heat maps for 7-day readmission and in-hospital mortality are presented in Figures S4.1 and S4.2.

We present heat maps to visualize how changes in hospital risk-standardized rates are associated with differences in the distribution of equity-related factors by hospital (Figure 3 presents 30-day readmission data, Figure S4.1 presents mortality data, and Figure S4.2 presents 7-day readmission data). We observe a visible gradient for homelessness and the ON-Marg housing and dwellings domain, with hospitals who treat more patients marginalized in those factors tending to have larger relative reductions in risk-standardized rates. Hospitals with the most marginalized patients in terms of housing and homelessness had the largest relative decreases in risk-standardized rates. This trend was not apparent in the other equity-related factors.

One hospital (hospital 103) had a 5.6% relative reduction in risk-standardized 30-day readmission rate (reclassified from above average to average) and a 7.3% relative reduction in risk-standardized 7-day readmission rate (average in both cases). This relative reduction was larger than the second-largest reductions of 2.4% for both 30-day and 7-day readmissions. This hospital was an outlier in terms of the proportion of patients experiencing homelessness and in the most marginalized quintile of the ON-Marg housing domain (Figures S5.1-5.6).

## Discussion

This study of a heterogeneous cohort of general medicine hospital admissions across 28 Ontario hospitals found that adjustment for disability, homelessness, and neighborhood marginalization meaningfully influenced hospitals’ risk-standardized 30-day readmission rates but not in-hospital mortality or 7-day readmission. This adjustment resulted in 5 of 23 hospitals in the reporting period (22%) being reclassified, 4 of which were reclassified to average. Homelessness and neighborhood-level marginalization related to housing appear to be most associated with changes in risk-standardized 30-day readmission rates. Including these specific equity-related factors in risk-adjustment models had relatively little effect on overall model performance.

A small body of literature has examined how equity-related factors influence hospital risk-adjustment comparisons. These studies predominantly were conducted in the United States, and they found that the inclusion of individual- and neighborhood-level equity-related factors (eg, race, disability, housing, and neighborhood-level marginalization) can account for differences in several disease-specific care process measures such as blood pressure screening, hemoglobin A_1c_ measurement, statin medication prescription, and cost of diabetes-related hospitalization and readmission.^66–68^ These studies identified that adjustment for equity-related factors in payment for care process measures reduced physician-group–level and institution-level variance and increased payment to institutions caring for the most marginalized patient populations.^66–68^ This literature has important limitations. These studies concentrated on individual care processes and did not assess performance across a range of diagnoses. They did not adjust for a comprehensive set of clinical characteristics, often only adjusting for age, sex, and comorbidities; thus, it is unclear whether adjustment for equity-related factors is impactful after more granular adjustment for illness severity and main diagnoses. Our study advances this literature by evaluating readmission and mortality rates within the entire general medicine cohort at 28 hospitals and using a high-quality and granular clinical risk adjustment method. Our findings are aligned with prior reports showing the impact of equity-related variables on individual hospital profiling.^9^ We have similarly identified that this difference in hospital performance measurement is most notable for those hospitals that serve the greatest proportion of marginalized patients.

Our findings suggest the importance of equity-related factors in risk adjustment may be influenced by the other included covariates, study population, and outcomes of interest. We theorize that disability, homelessness, and neighborhood marginalization could potentially affect prehospital factors (eg, complexity at the time of presentation), in-hospital factors (eg, variation in care received), and posthospital factors (eg, access to follow-up care, medications, and assistance with living independently). Given our finding that adjustment for these equity-related factors had the greatest effect on risk adjustment for 30-day readmission and had little effect on outcomes that are more proximate to the hospitalization itself (ie, mortality and 7-day readmission), it is possible that disability, homelessness, and neighborhood marginalization act most on posthospital factors. This corresponds with other studies showing that both individual and neighborhood equity-related variables influence the risk of readmission.^13^^,^^69–71^ The inclusion of a comprehensive set of clinical characteristics may have attenuated prehospital or in-hospital effects of these equity-related factors on mortality and 7-day readmission. Seto et al.^72^ previously found that race predicted inpatient death among cardiac surgery patients when clinical covariates were limited to binary comorbid conditions but was no longer predictive when clinical severity data (eg, laboratory values) were included.^72^ Furthermore, Davis et al.^73^ found in Alberta, Canada, that neighborhood-level measures of marginalization did not improve prediction models for readmission after considering granular clinical data.

Our findings should be contextualized within the Ontario health care system, which provides universal insurance for hospital care to all residents irrespective of their ability to afford insurance. This may mitigate selection effects across hospitals (ie, all patients are eligible to attend all hospitals), which may reduce equity-related differences across hospitals compared with other jurisdictions, like the United States, where hospital case mix could be influenced by insurance coverage. Furthermore, Ontario has 1 of the most diverse populations in the world with respect to race/ethnicity, language, and country of origin.^74–77^

Our study has several limitations. We included only selected equity-related measures that could be measured reliably using established methods in Ontario. These did not include important equity-related factors, such as race/ethnicity, language, and social support, which are associated with patient outcomes^78–87^ but data on these factors are not collected reliably in the Canadian health care system. Our findings should be interpreted as specific to the included equity-related factors: disability, homelessness, and neighborhood marginalization. We can make no conclusions about the impact of adjustment for other equity-related factors. For example, minoritized race/ethnic groups generally have poorer health outcomes than nonminoritized groups.^88–93^ We were able to measure some factors that might explain this disparity, such as clinical severity of presenting conditions or neighborhood socioeconomic exposures, but we were not able to account for individual exposures, such as interpersonal racism. Thus, the absence of individual equity-related factors may lead to an underestimation of the impact of equity-related factors on risk adjustment. Given the importance of measuring equity-related differences in care, we would advocate for concerted and thoughtful efforts to collect individual-level equity data across Canada and internationally.^40^ There are early examples of such efforts in a Canadian primary care context.^94–96^ Our approach to identifying homeless patients likely has low sensitivity because we did not have access to all data sources in the Richard et al. algorithm.^52^ Furthermore, our analysis considered homelessness as a binary variable because we are not able to accurately differentiate between sheltered and unsheltered homelessness. Disability was measured using diagnosis codes, and we did not have self-reported disability information.^94–96^ This may have affected what types of disabilities were included and which may reduce the relative impact of disabilities that were missed in the coding or that were collinear with other comorbidities included in the Charlson Comorbidity Index.

Within Ontario’s universal health care system, the inclusion of disability, homelessness, and neighborhood marginalization to risk-adjusted hospital performance measures meaningfully influenced risk-standardized 30-day readmission rates but not in-hospital mortality or 7-day readmission rates. Adjustment made little difference to the accuracy of risk-adjustment models in all 3 outcomes. Our findings can inform researchers and policymakers as they consider when to adjust for these equity-related factors in hospital performance measurement.

## Acknowledgments

The authors acknowledge the individuals and organizations who made the data available for this research.

## Supplementary Material

Web_Material_kwae401

## Data Availability

Unlimited open access to GEMINI data is unavailable because of data-sharing agreements and research ethics board protocols with participating hospitals. However, researchers can request access to GEMINI data through an established process approved by our institutional research ethics boards. Please see full details at https://www.geminimedicine.ca/access-data.
