# Supplementary material for: Effect of disability, homelessness, and neighborhood marginalization on risk adjustment for hospital performance measurement
Source: Am J Epidemiol. 2024 Oct 16;194(11):3079–90. doi: 10.1093/aje/kwae401 (PMC12634112; doi:10.1093/aje/kwae401)
Supplement: Web_Material_kwae401 [file web_material_kwae401.docx]

Supplementary Tables and Figures

[Table S1: Top 10 Most Responsible Discharge Diagnoses 2](#_Toc178347294)

[Figure S1.1: Calibration curves for in-hospital mortality models. 3](#_Toc178347295)

[Figure S1.2: Calibration curves for 7-day readmission models. 4](#_Toc178347296)

[Figure S2.1: In-hospital mortality: Forest plots of risk adjustment model performance metrics. 5](#_Toc178347297)

[Figure S2.2: 30-day readmission: Forest plots of risk adjustment model performance metrics. 9](#_Toc178347298)

[Figure S2.3: 7-day readmission: Forest plots of risk adjustment model performance metrics. 13](#_Toc178347299)

[Figure S3.1: Caterpillar plot of risk-standardized in-hospital mortality rates. 17](#_Toc178347300)

[Figure S3.2: Caterpillar plot of risk-standardized 7-day readmission rates. 18](#_Toc178347301)

[Figure S4.1: Heatmap to visualize how changes in hospital risk-standardized in-hospital mortality rate are associated with disability, homelessness, and neighborhood marginalization 19](#_Toc178347302)

[Figure S4.2: Heatmap to visualize how changes in hospital risk-standardized 7-day readmission rate are associated with disability, homelessness, and neighborhood marginalization 20](#_Toc178347303)

[Figures S5.1-5.6. Distribution of equity-related factors by hospital 21](#_Toc178347304)

### Table S1: Top 10 Most Responsible Discharge Diagnoses

Most responsible discharge diagnosis were defined by the International Classification of Diseases and Related Health Problems, 10th edition Canada (ICD-10-CA) codes, which were further grouped into clinically relevant categories using the Clinical Classification Software Refined (CCSR).^36-39^

| In-Hospital Mortality Cohort  N = 544,805 | | 7-day Readmission Cohort  N = 442,418 | | 30-day Readmission Cohort  N = 441,009 | |
| --- | --- | --- | --- | --- | --- |
| Heart failure | 28,185  (5.2%) | Heart failure | 24,344  (5.5%) | Heart failure | 24,262 (5.5%) |
| Pneumonia (except that caused by tuberculosis) | 21,187  (3.9%) | Pneumonia (except that caused by tuberculosis) | 18,436  (4.2%) | Pneumonia (except that caused by tuberculosis) | 18,385  (4.2%) |
| Chronic obstructive pulmonary disease and bronchiectasis | 19,258  (3.5%) | Urinary tract infections | 18,136  (4.1%) | Urinary tract infections | 18,075 (4.1%) |
| Urinary tract infections | 19,205  (3.5%) | Chronic obstructive pulmonary disease and bronchiectasis | 17,283  (3.9%) | Chronic obstructive pulmonary disease and bronchiectasis | 17,235  (3.9%) |
| Neurocognitive disorders | 18,473  (3.4%) | Coronavirus disease - 2019 (COVID-19) | 12,519  (2.8%) | Coronavirus disease - 2019 (COVID-19) | 12,443  (2.8%) |
| Coronavirus disease - 2019 (COVID-19) | 18,185  (3.3%) | Diabetes mellitus with complication | 12,060  (2.7%) | Diabetes mellitus with complication | 12,025  (2.7%) |
| Septicemia | 16,293  (3.0%) | Cerebral infarction | 11,734  (2.7%) | Cerebral infarction | 11,696  (2.7%) |
| Cerebral infarction | 14,724  (2.7%) | Septicemia | 11,413  (2.6%) | Septicemia | 11,388  (2.6%) |
| Diabetes mellitus with complication | 13,506  (2.5%) | Acute and unspecified renal failure | 10,655  (2.4%) | Acute and unspecified renal failure | 10,623  (2.4%) |
| Acute and unspecified renal failure | 12,564  (2.3%) | Gastrointestinal hemorrhage | 10,338  (2.3%) | Gastrointestinal hemorrhage | 10,320  (2.3%) |

### Figure S1.1: Calibration curves for in-hospital mortality models.

Calibration curves for in-hospital mortality models, with and without adjustment for equity-related factors. Each curve represents a held-out hospital during internal-external cross-validation. The histogram on the top axis represents the distribution of predicted probabilities, and is not scaled to the y-axis.

Without equity-related adjustment
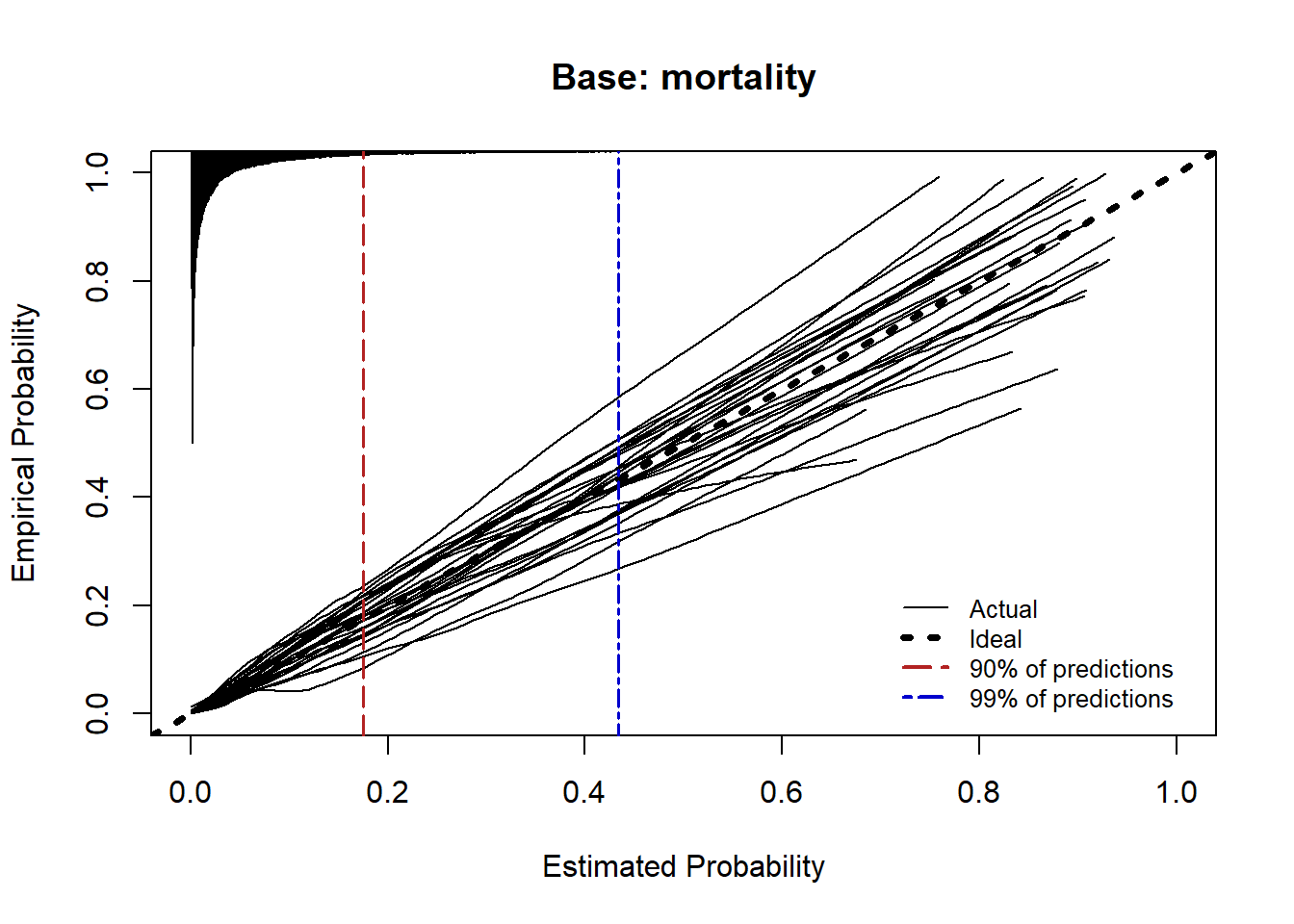


With equity-related adjustment
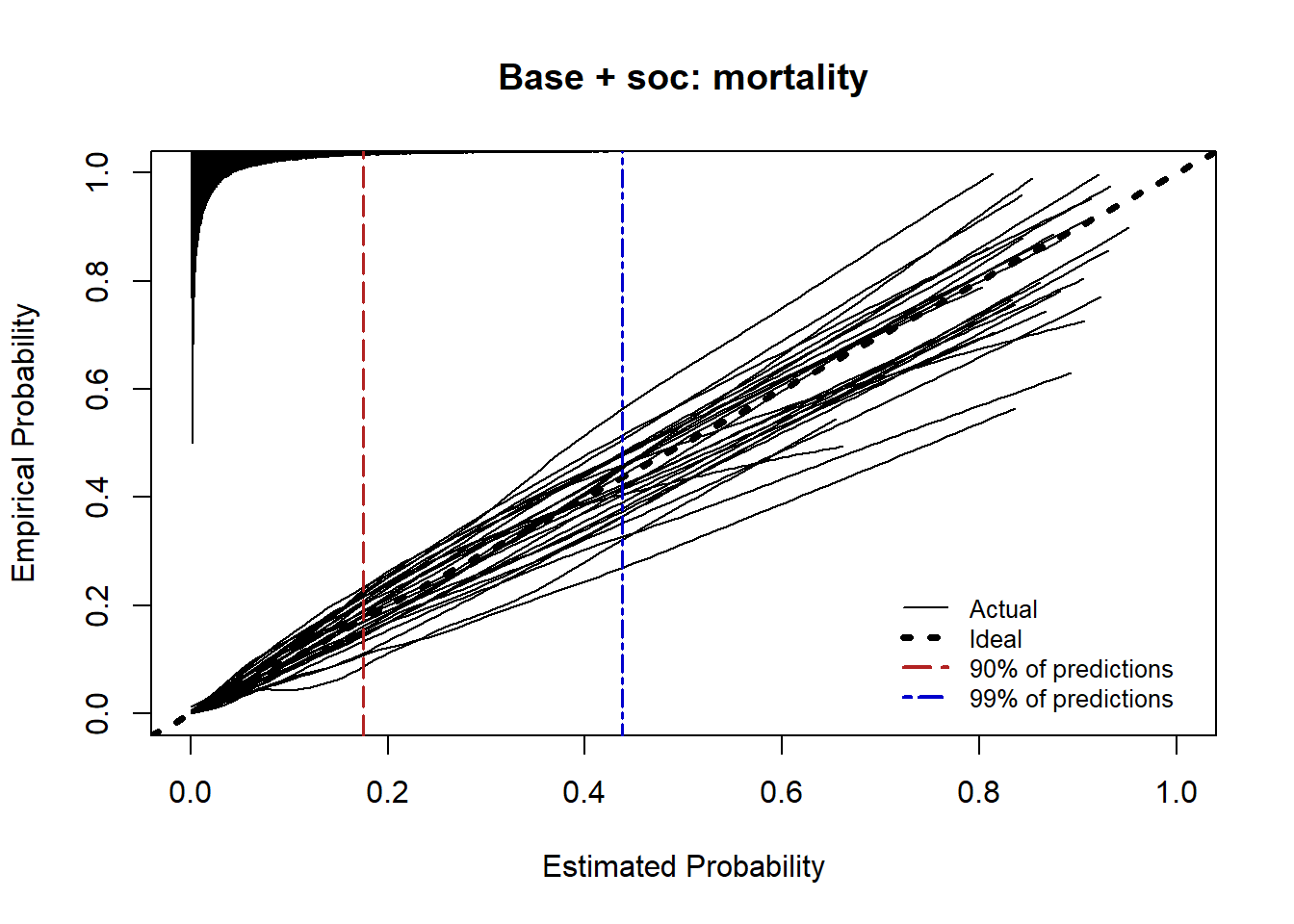


### Figure S1.2: Calibration curves for 7-day readmission models.

Calibration curves for 7-day readmission models, with and without adjustment for equity-related factors. Each curve represents a held-out hospital during internal-external cross-validation. The histogram on the top axis represents the distribution of predicted probabilities, and is not scaled to the y-axis.

Without equity-related adjustment


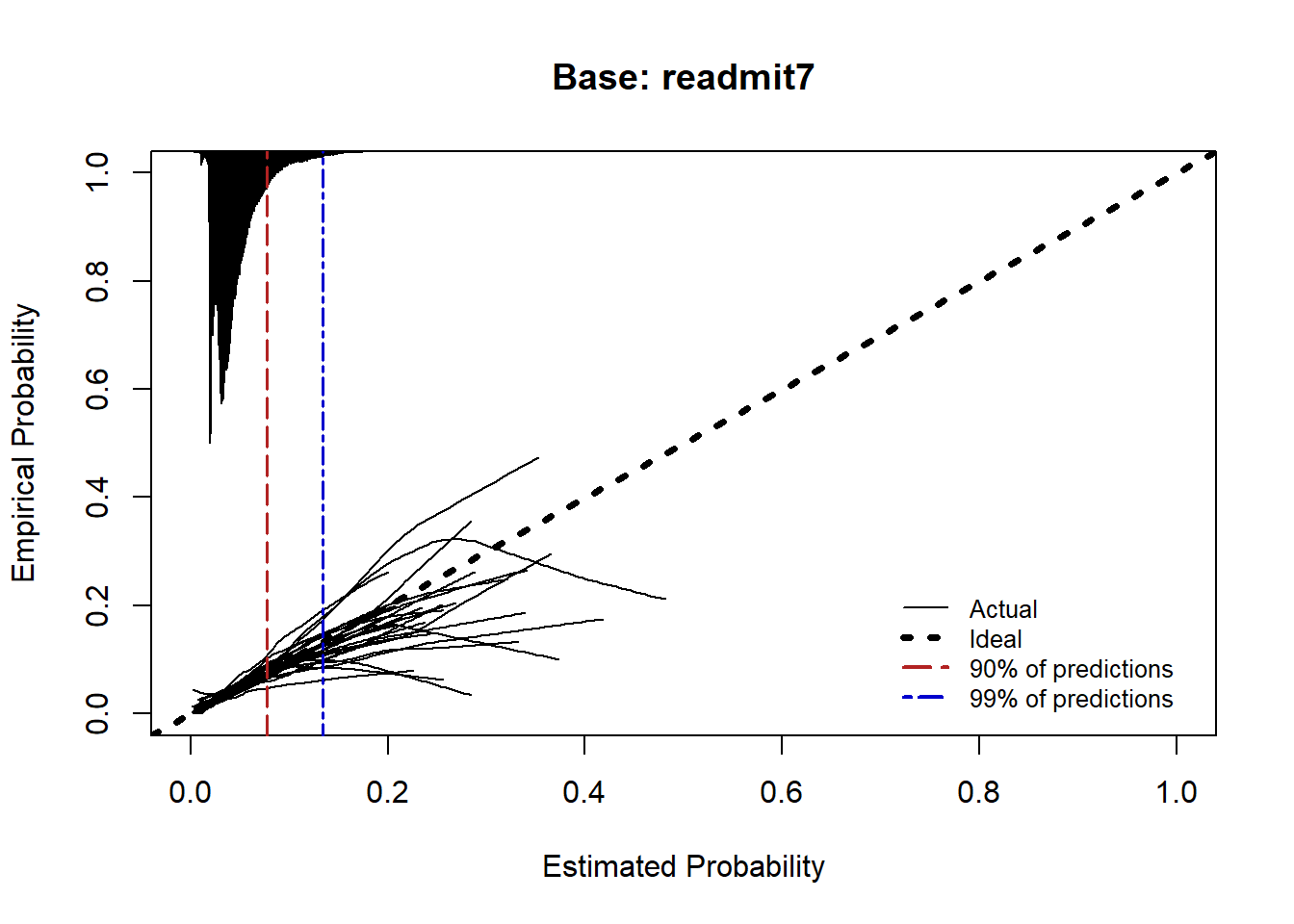


With equity-related adjustment


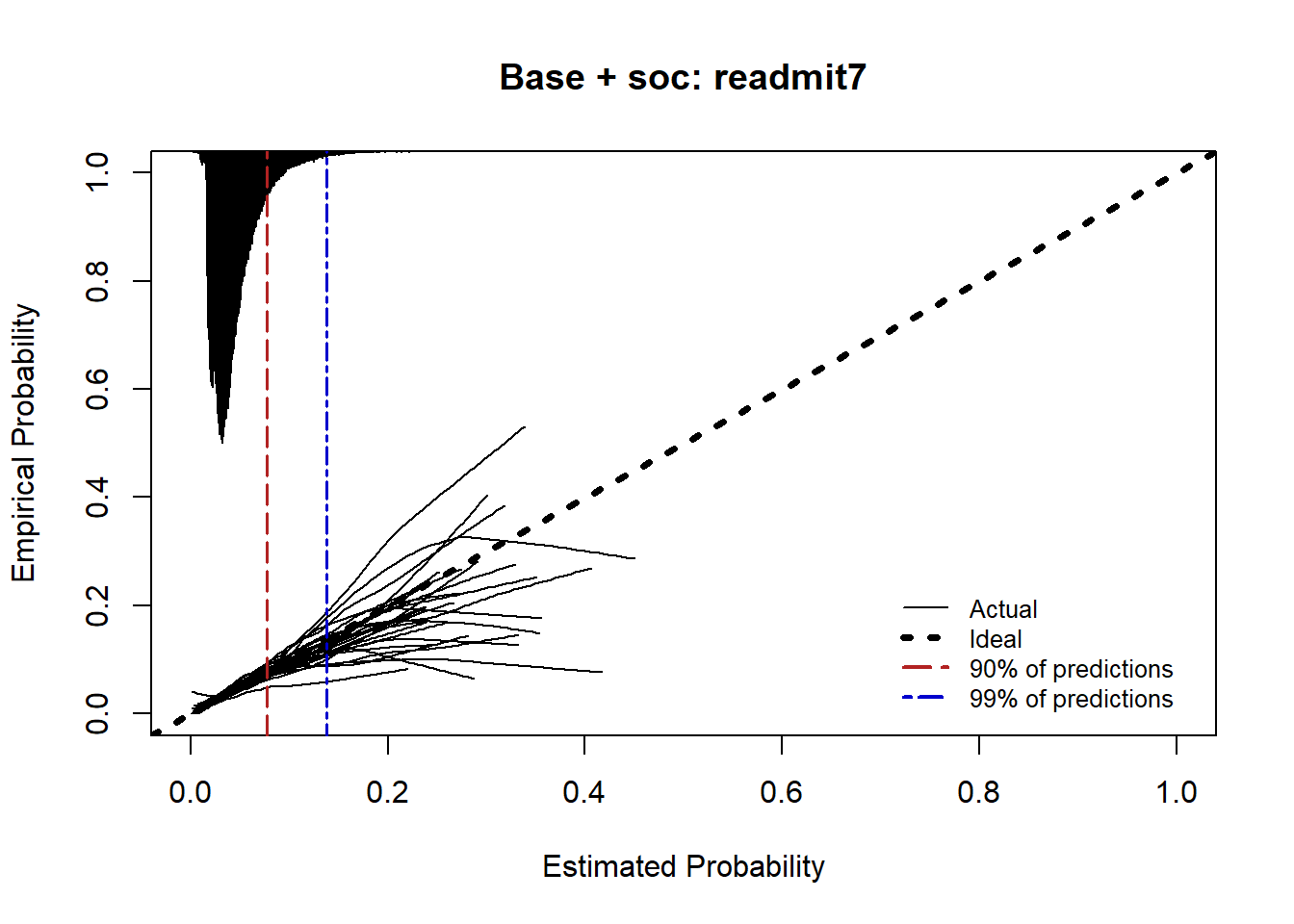


### Figure S2.1: In-hospital mortality: Forest plots of risk adjustment model performance metrics.

*Plots on the left are without equity-related adjustment, plots on the right are with equity-related adjustment*

Metrics were obtained through internal-external cross-validation where each hospital is held out separately, models are re-fit in the remaining 27 hospitals, and model performance is assessed in the held-out hospital.
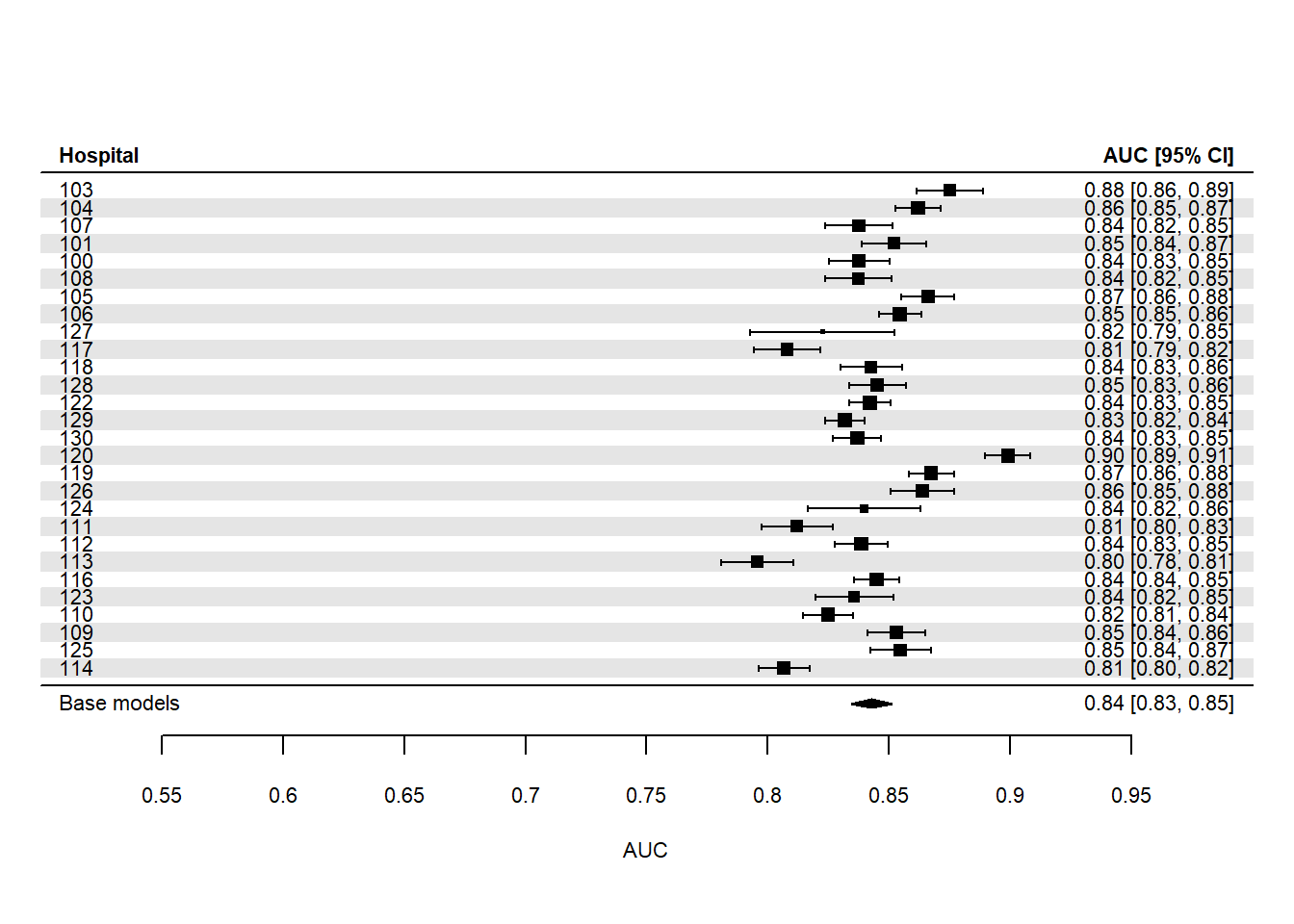

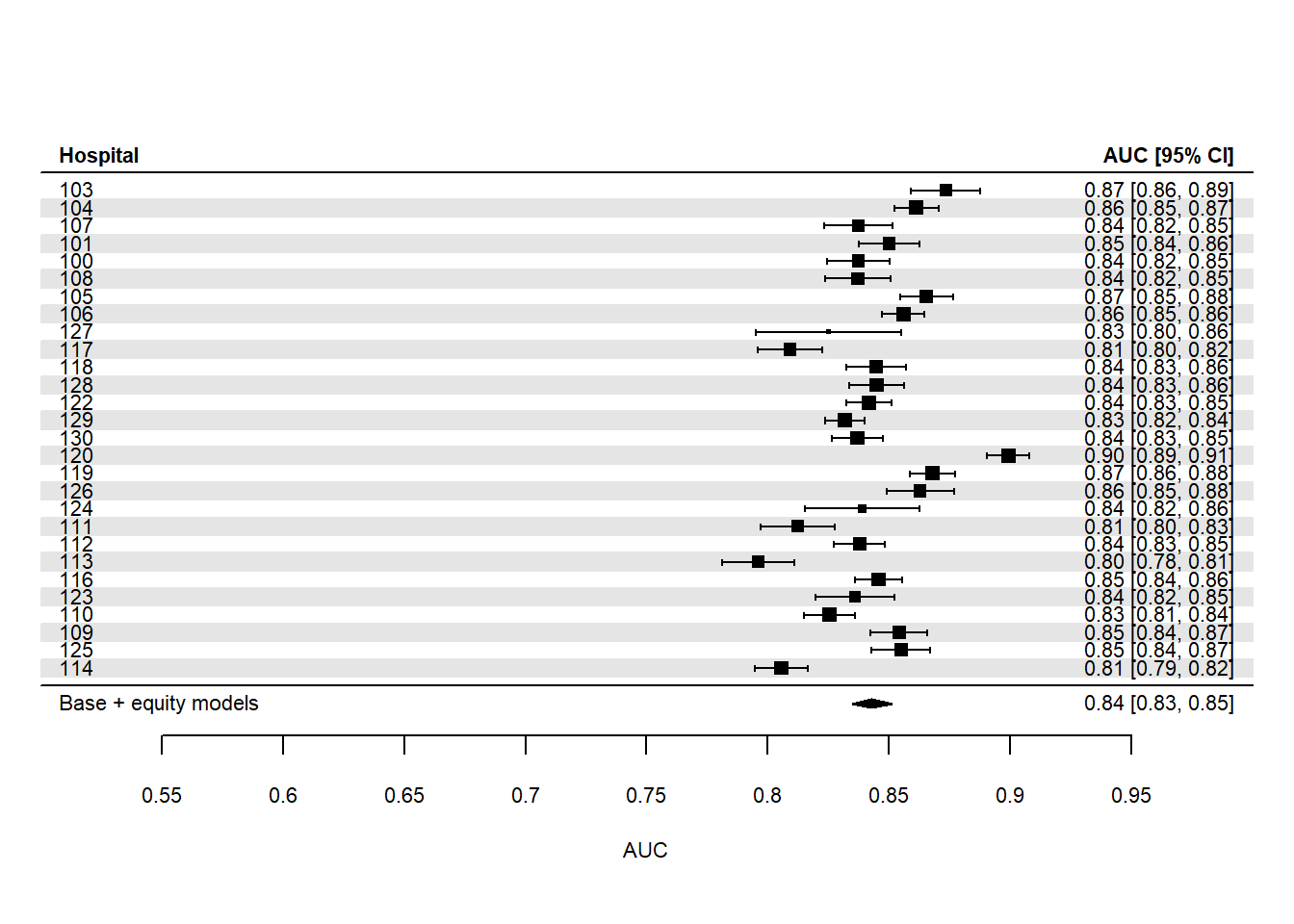


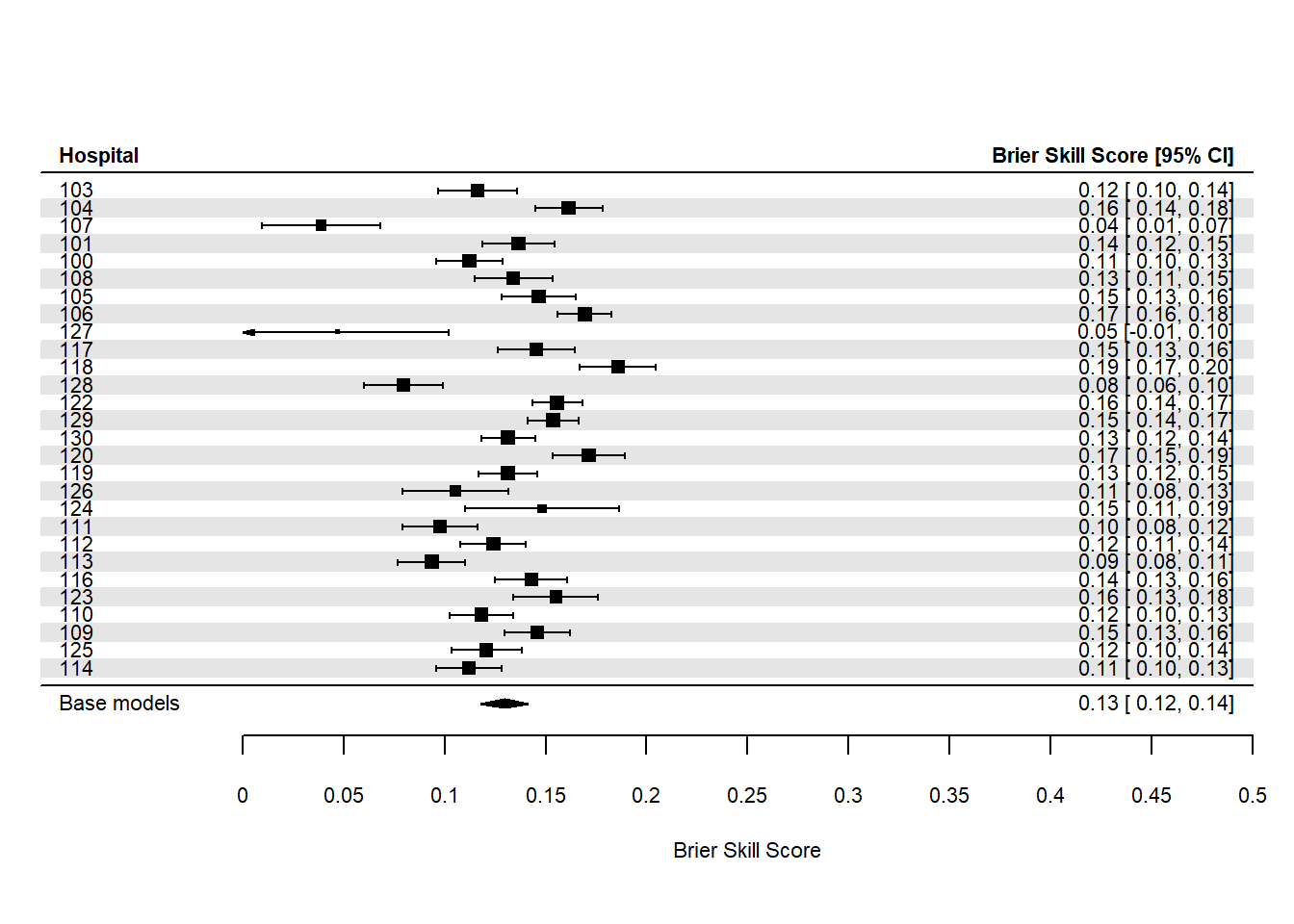

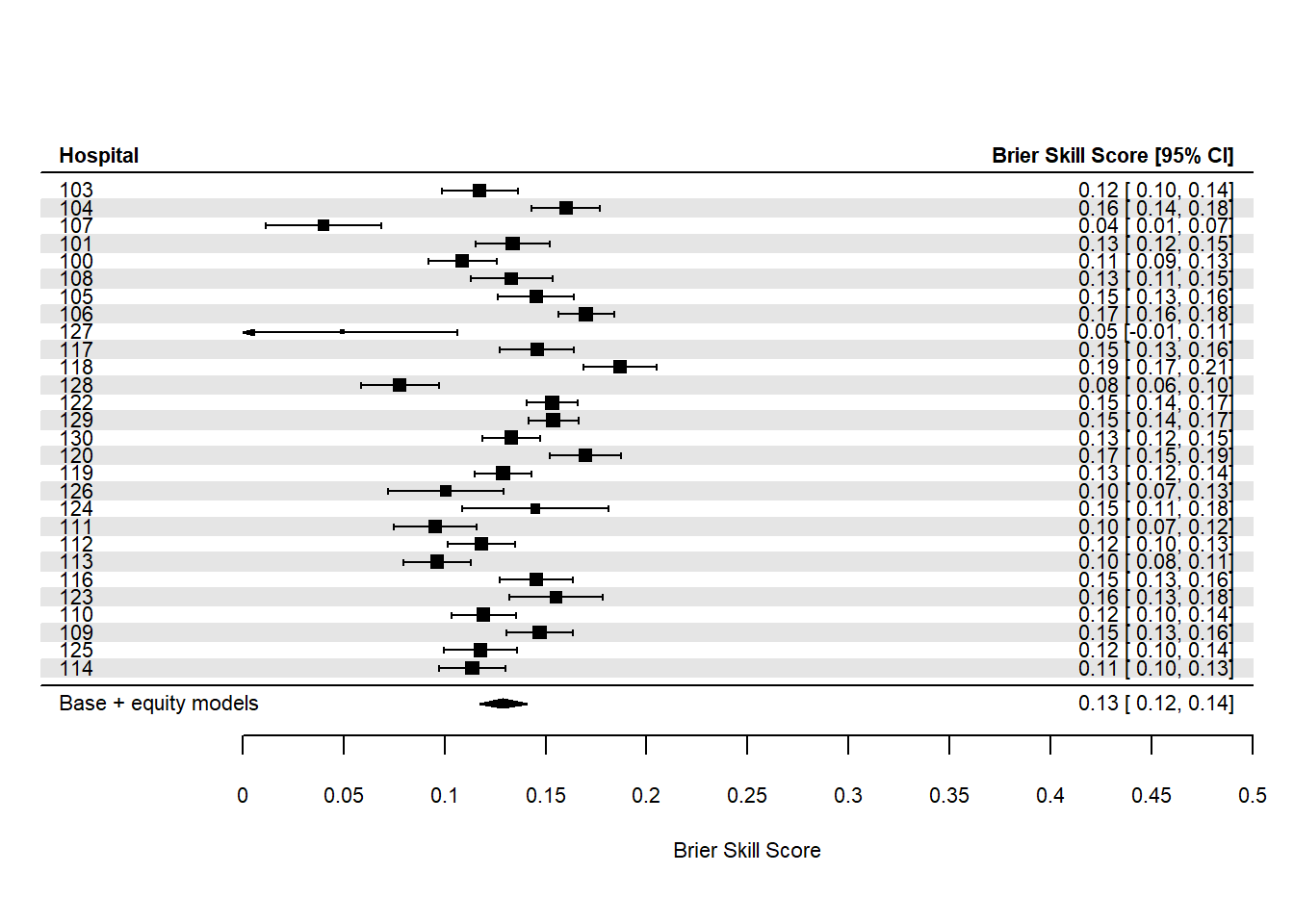


**Figure S2.1 continued: In-hospital mortality: Forest plots of risk adjustment model performance metrics.**
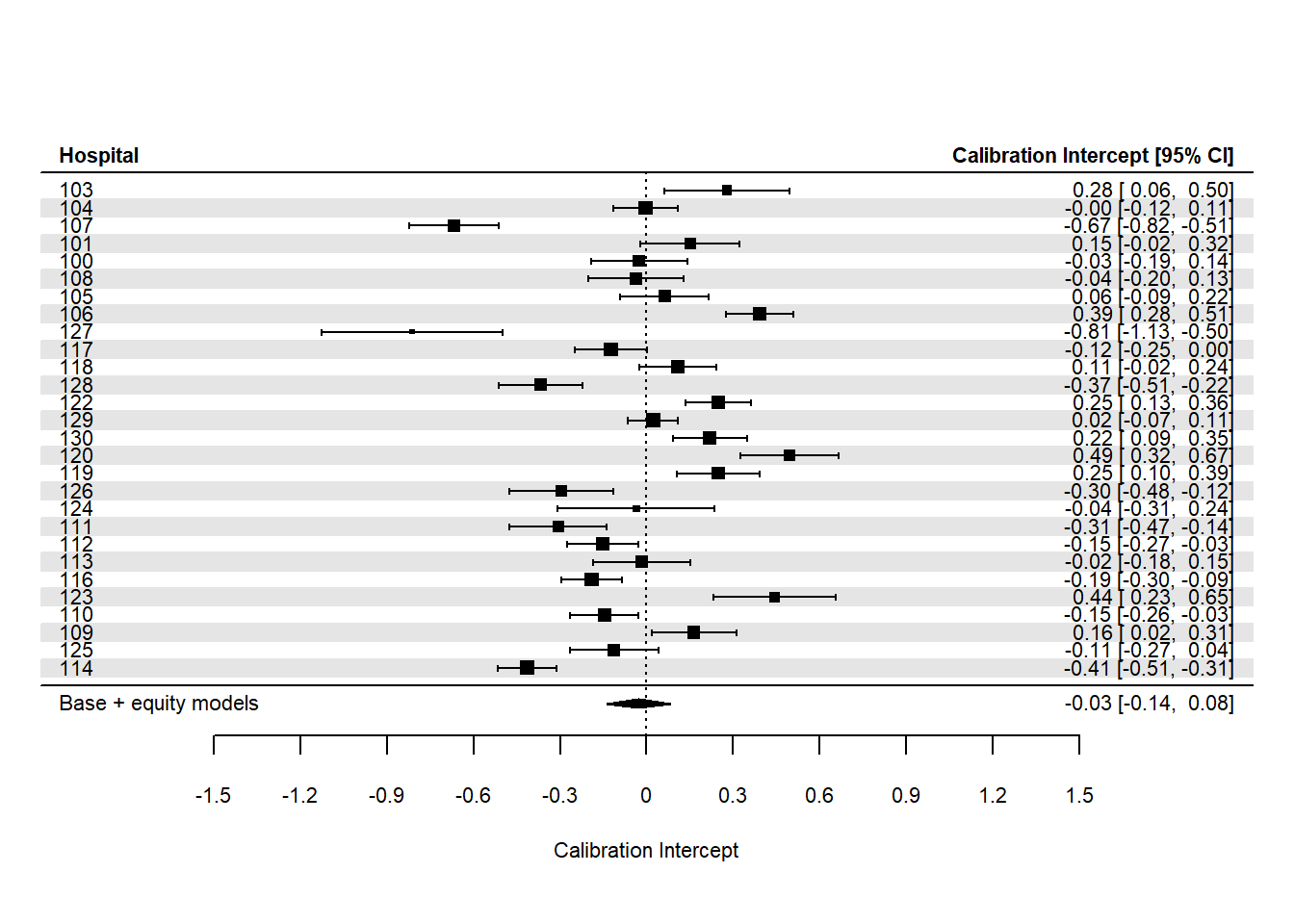

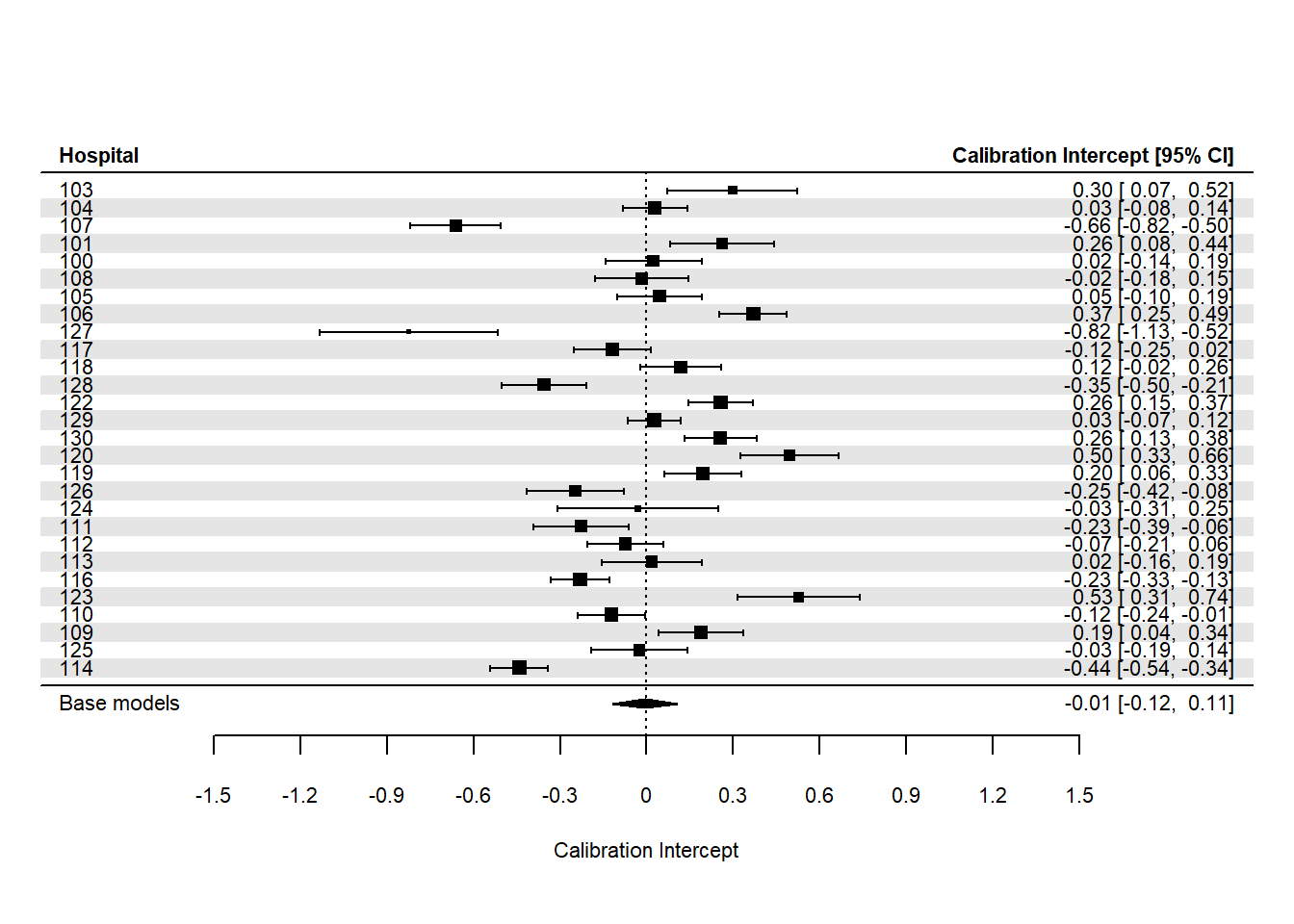


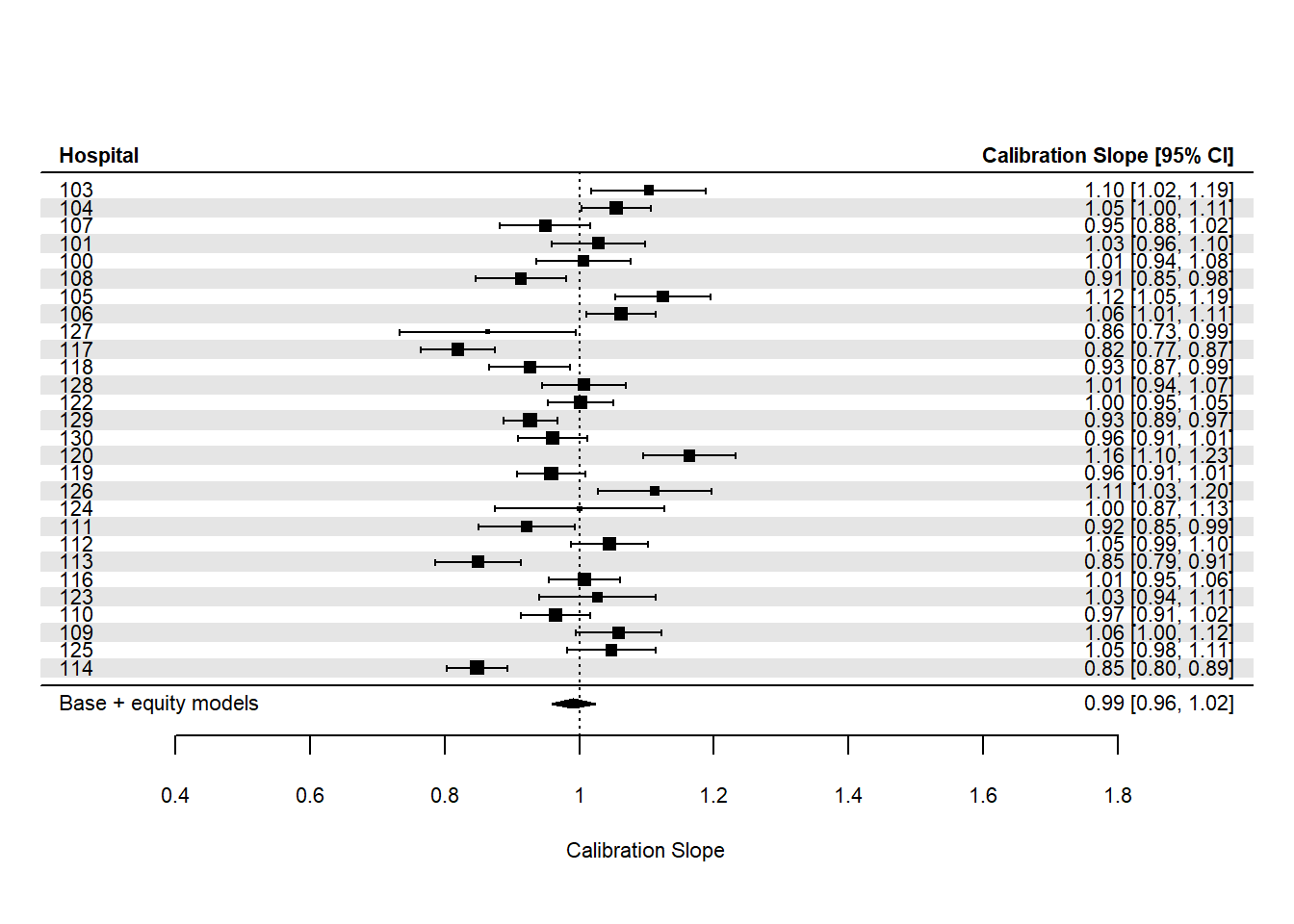

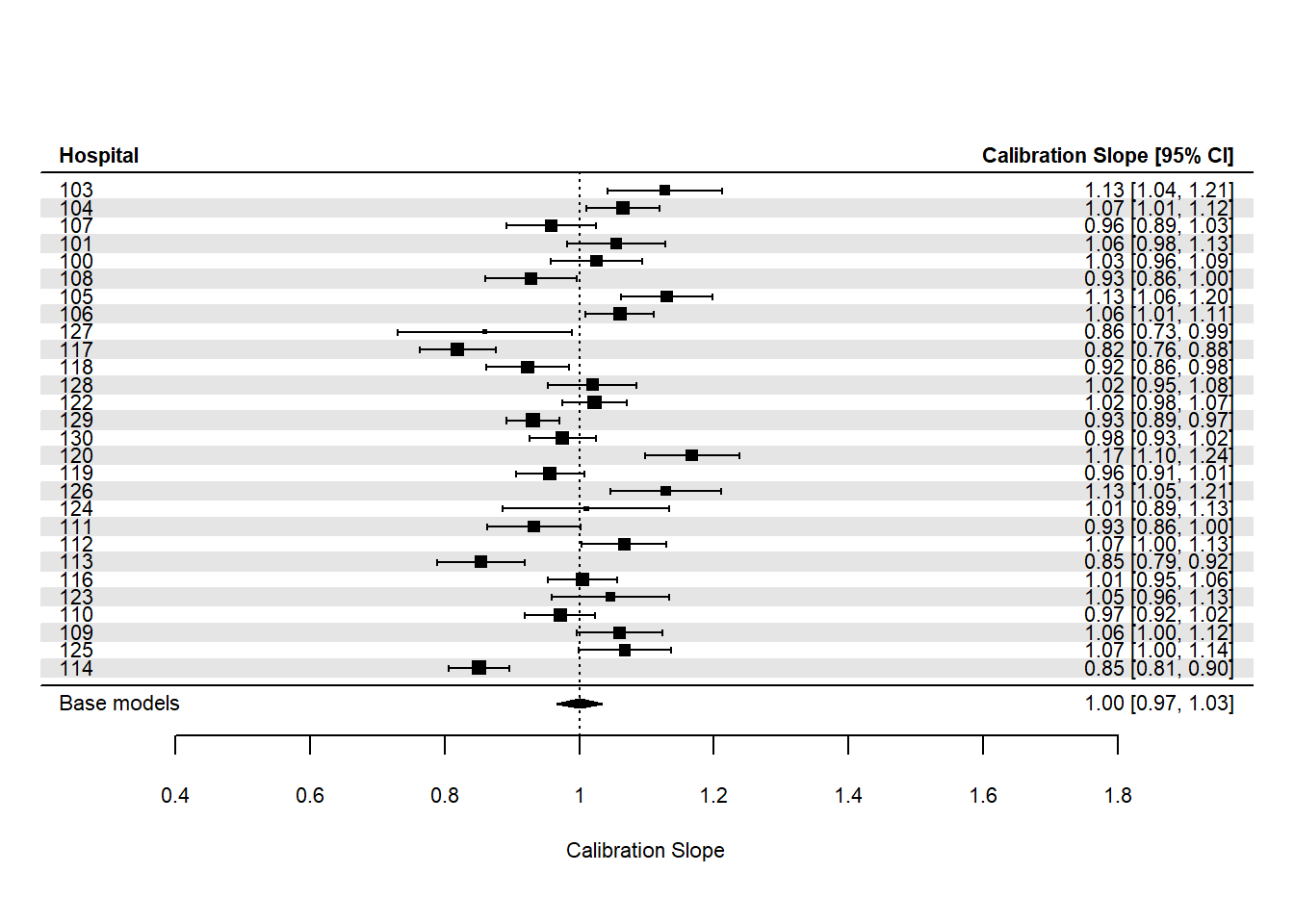


**Figure S2.1 continued: In-hospital mortality: Forest plots of risk adjustment model performance metrics.**


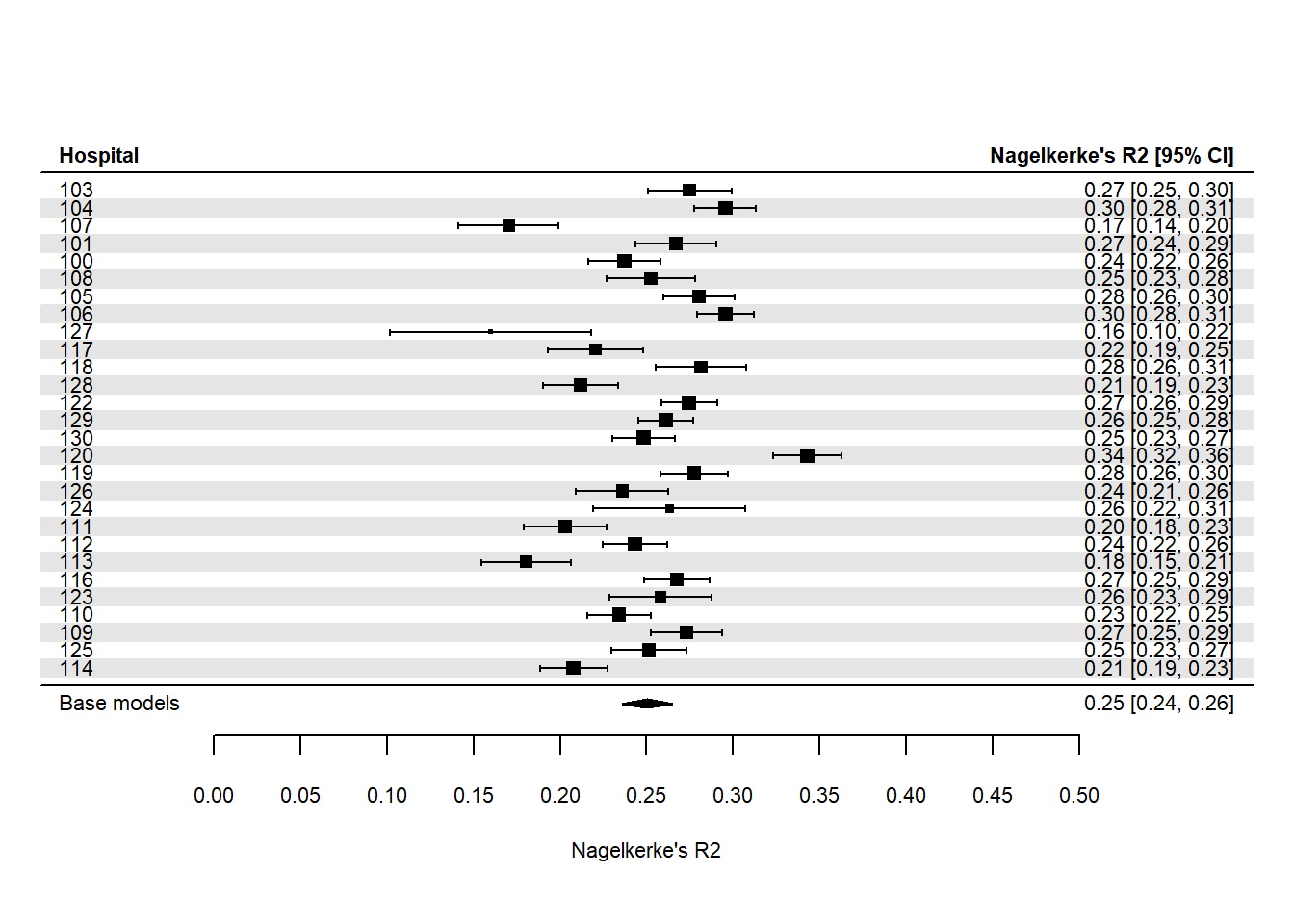

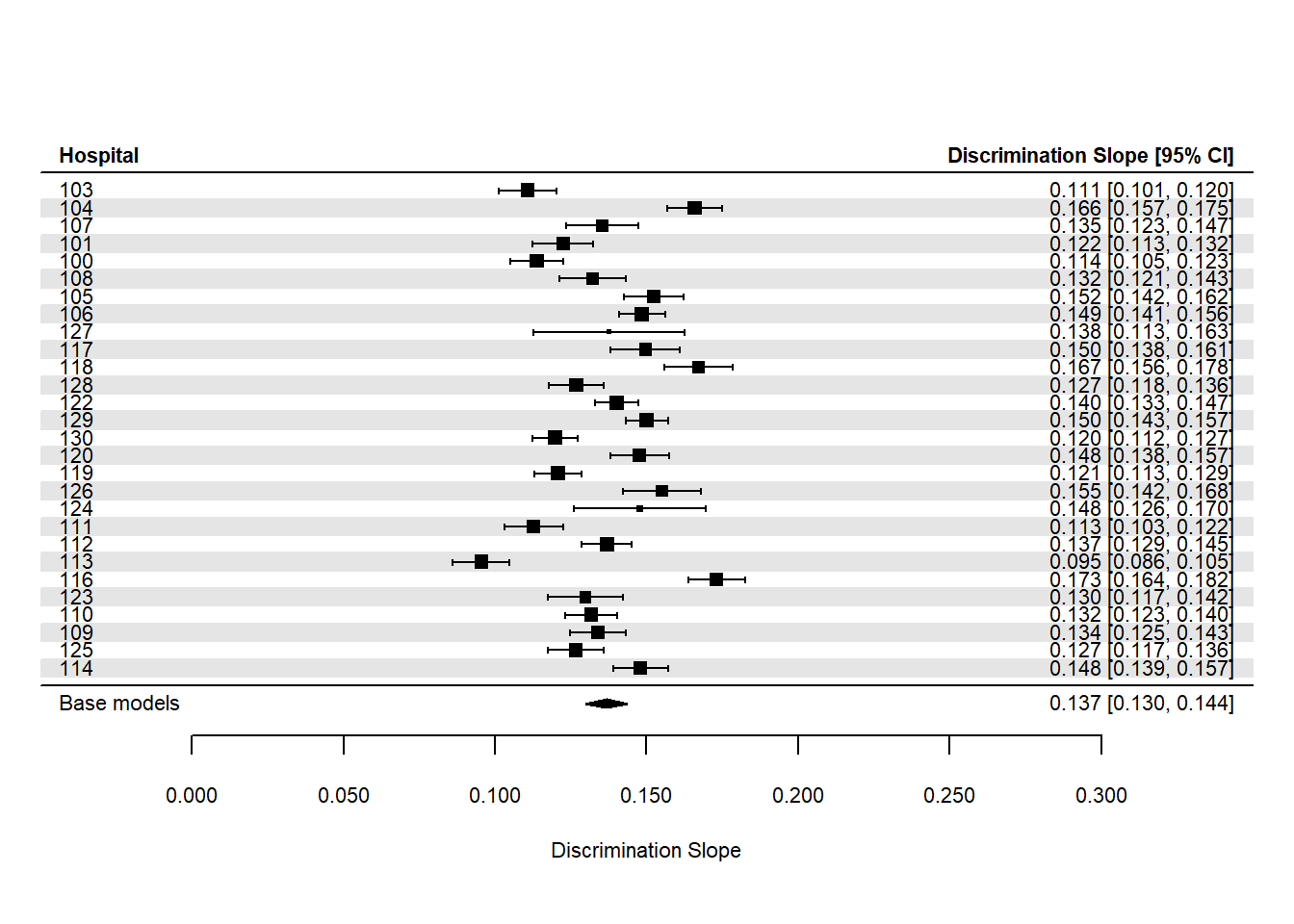

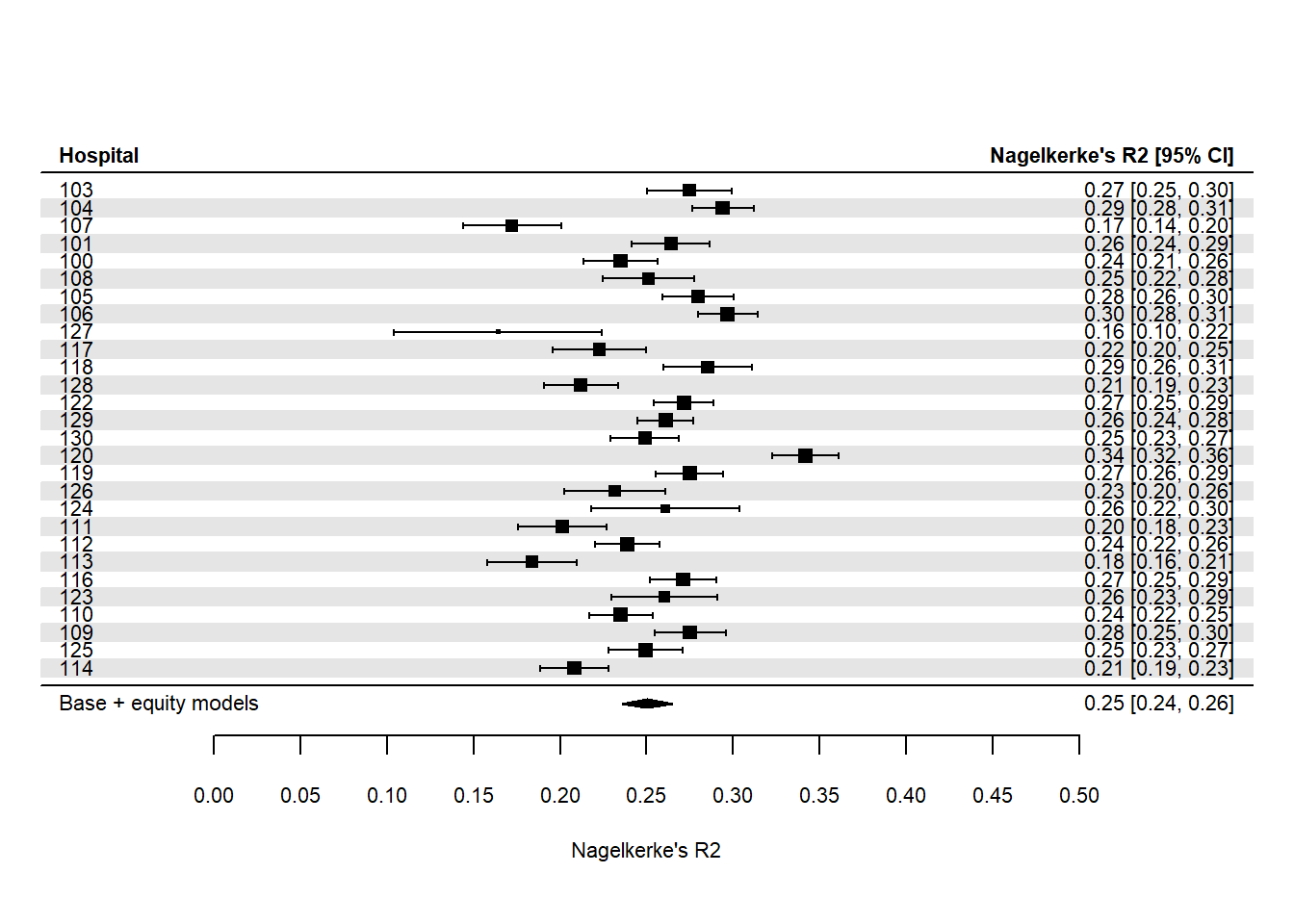

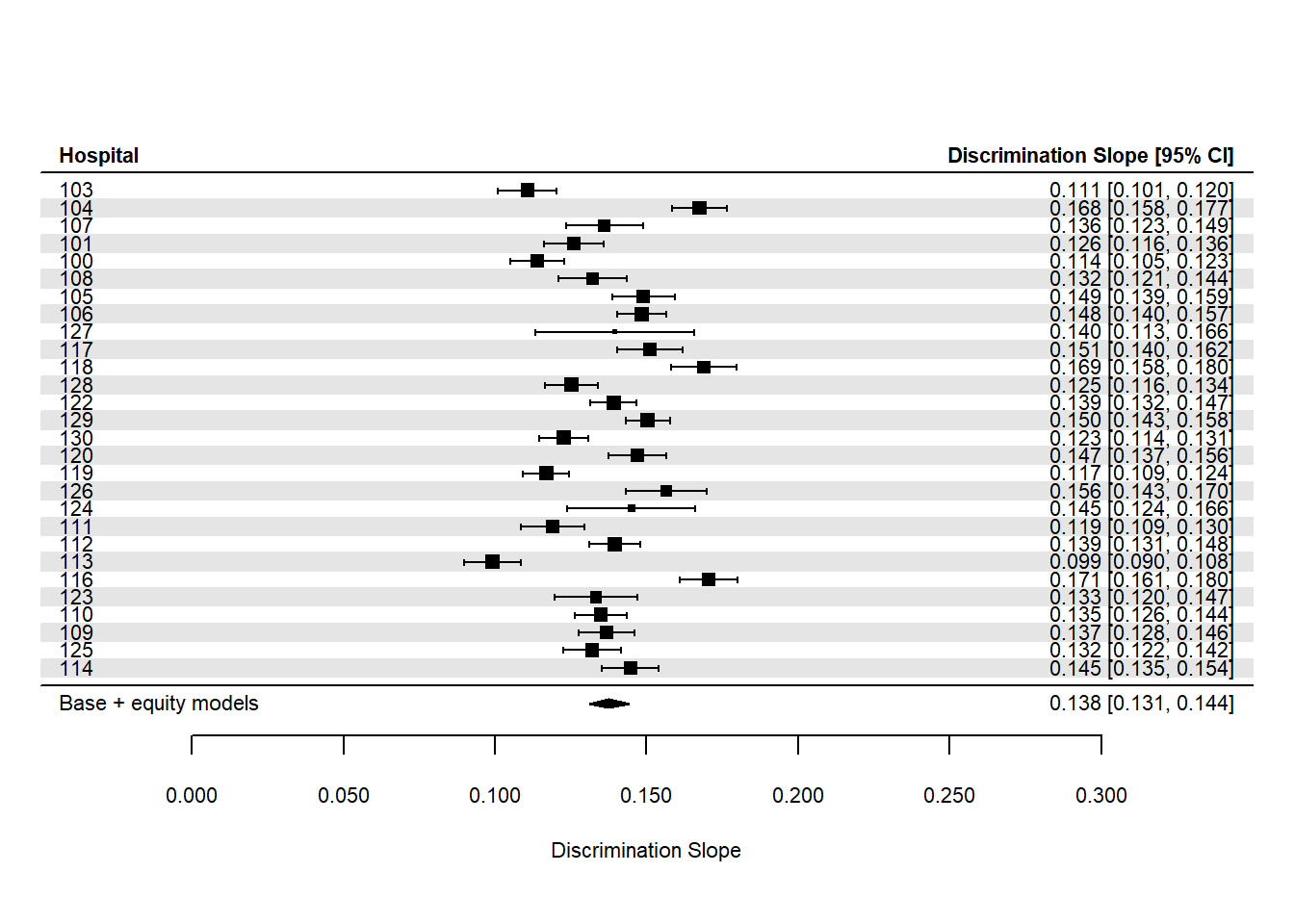


**Figure S2.1 continued: In-hospital mortality: Forest plots of risk adjustment model performance metrics.**


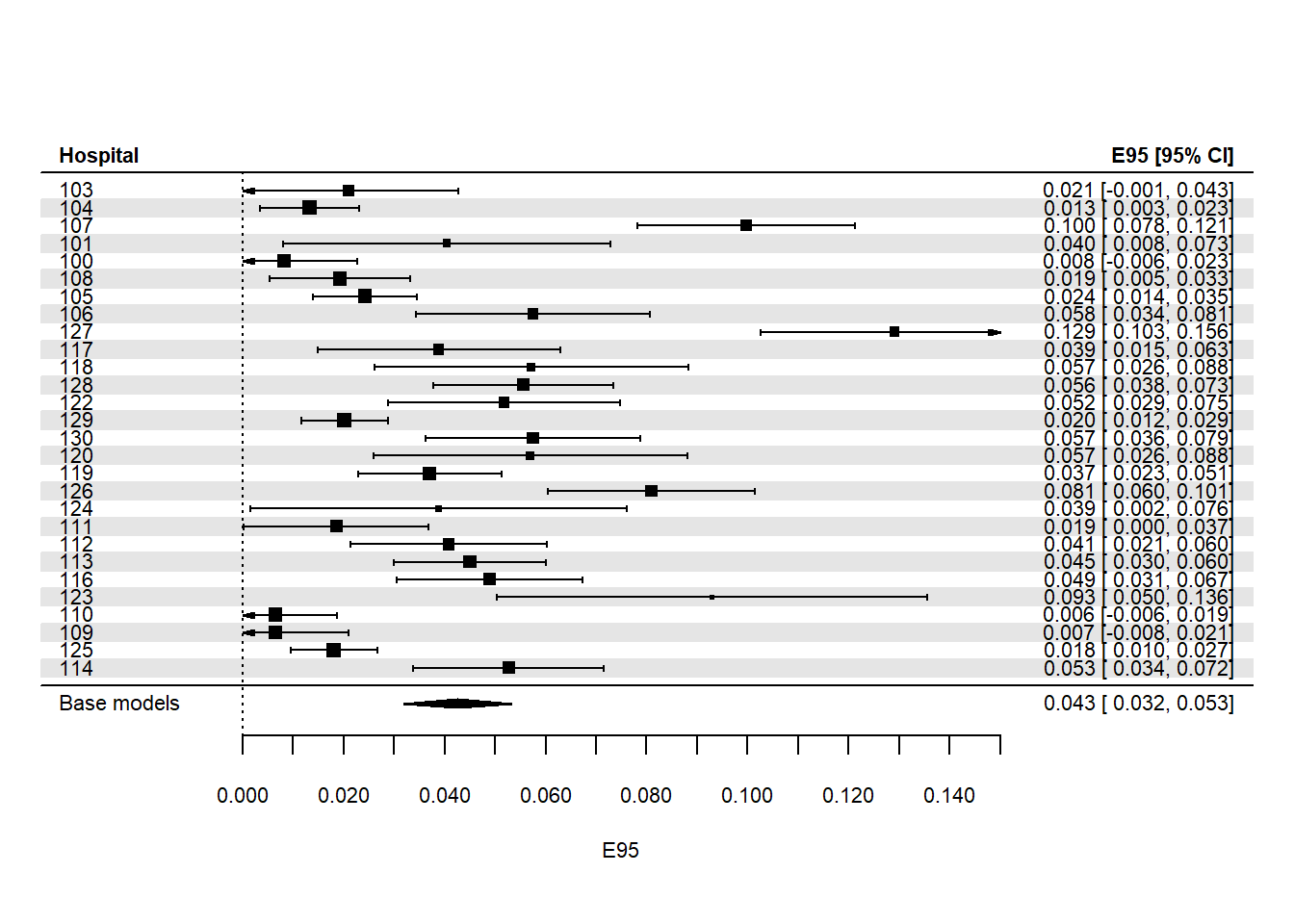

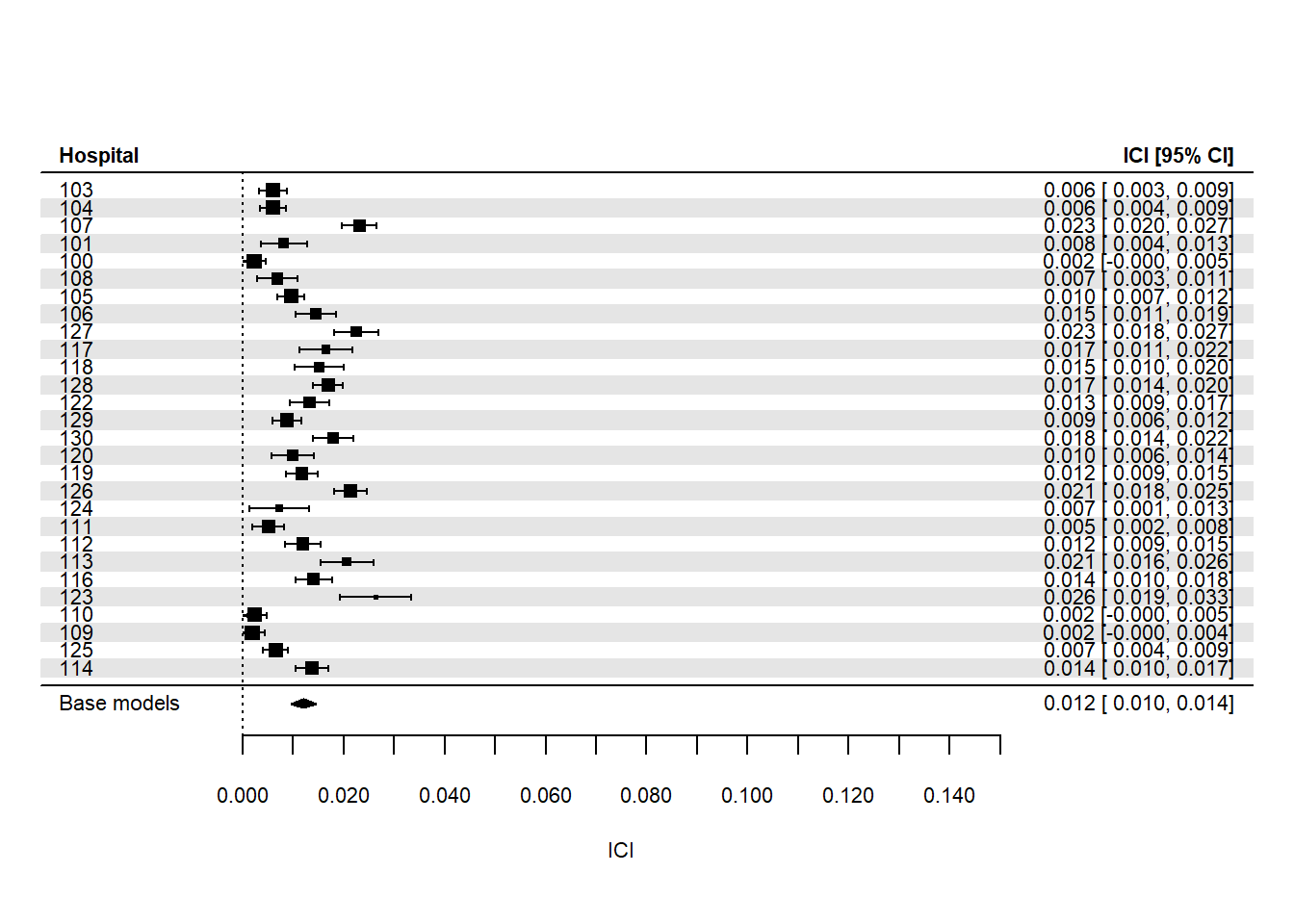

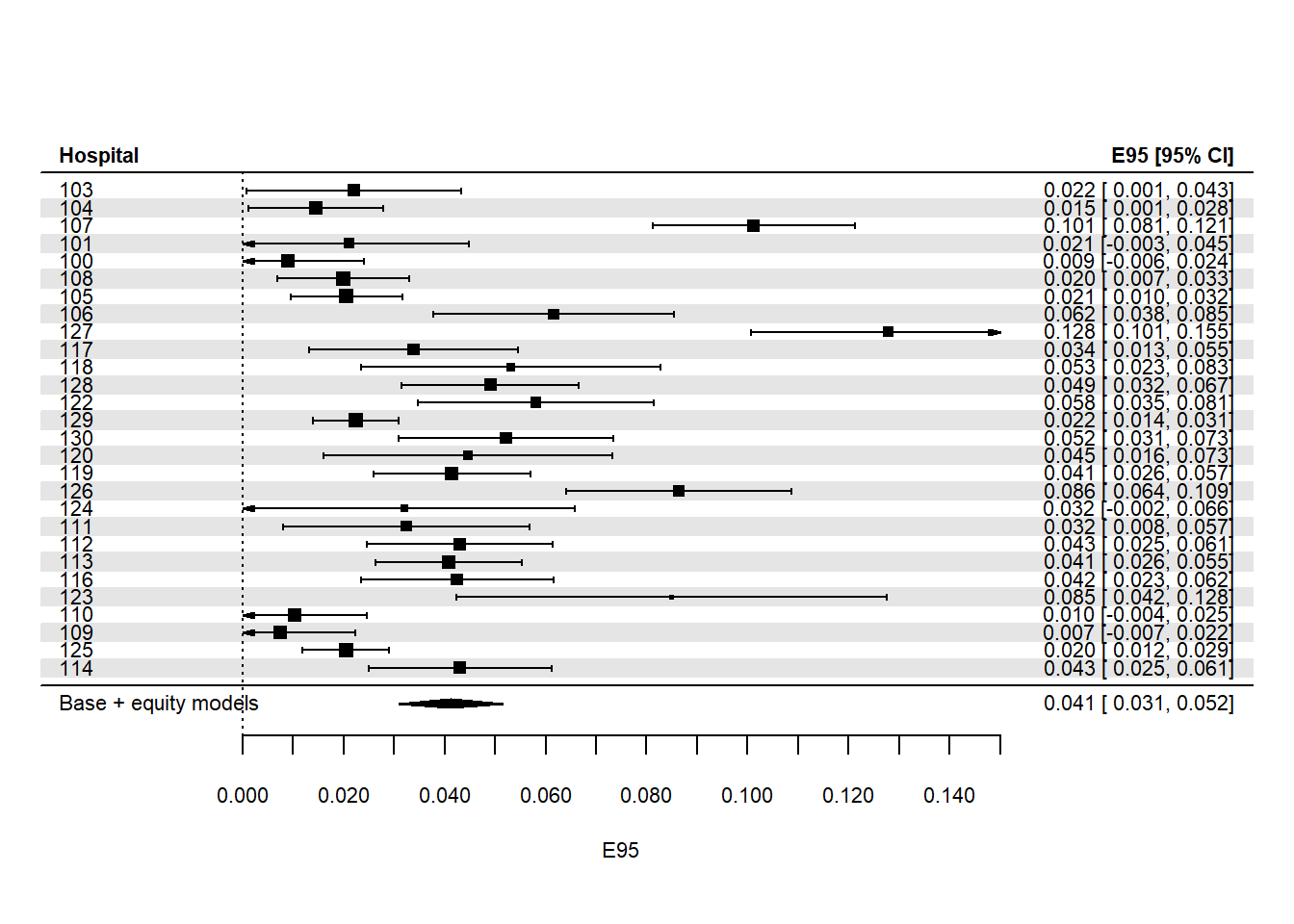

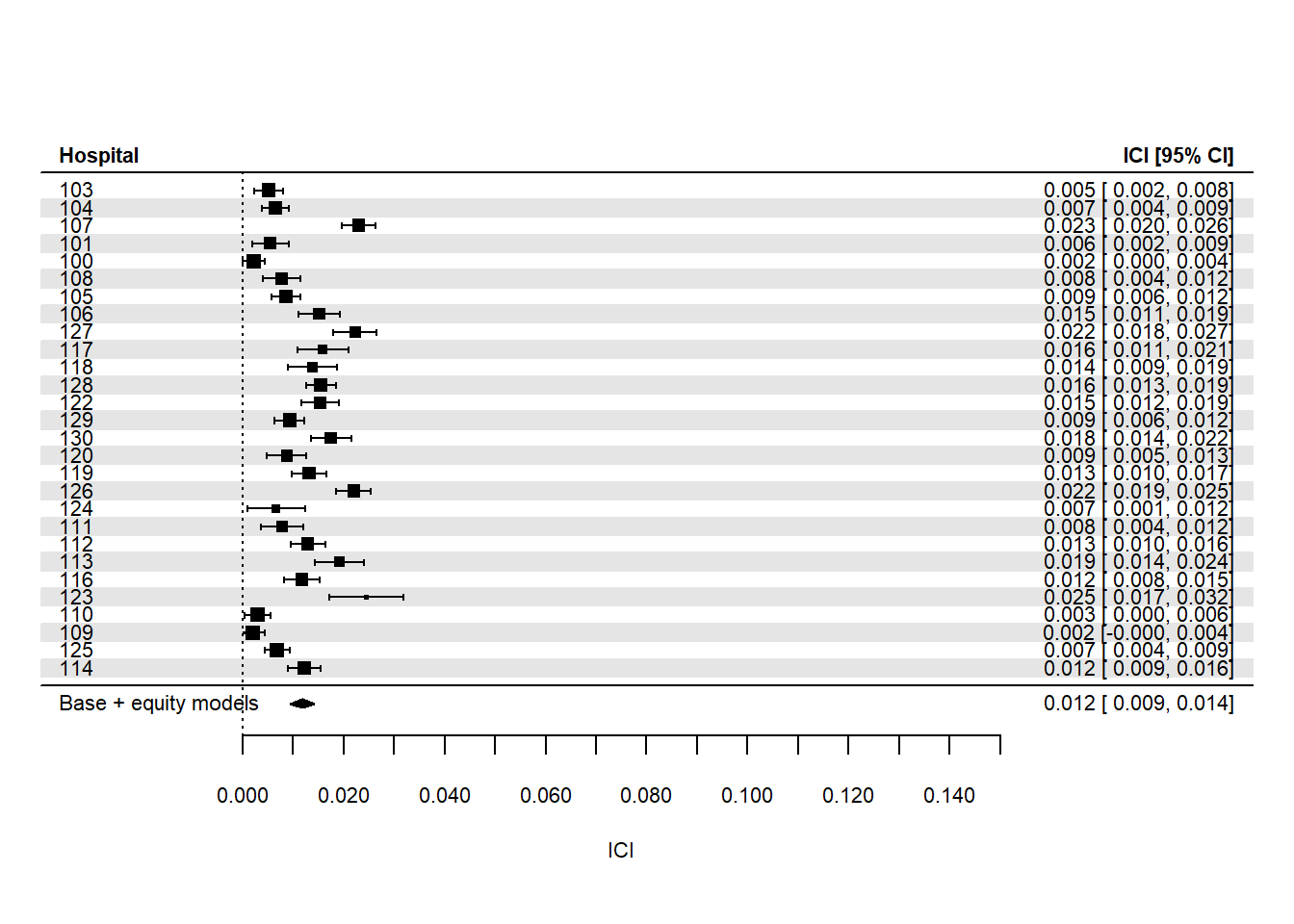


### Figure S2.2: 30-day readmission: Forest plots of risk adjustment model performance metrics.

*Plots on the left are without equity-related adjustment, plots on the right are with equity-related adjustment*

Metrics were obtained through internal-external cross-validation where each hospital is held out separately, models are re-fit in the remaining 27 hospitals, and model performance is assessed in the held-out hospital.


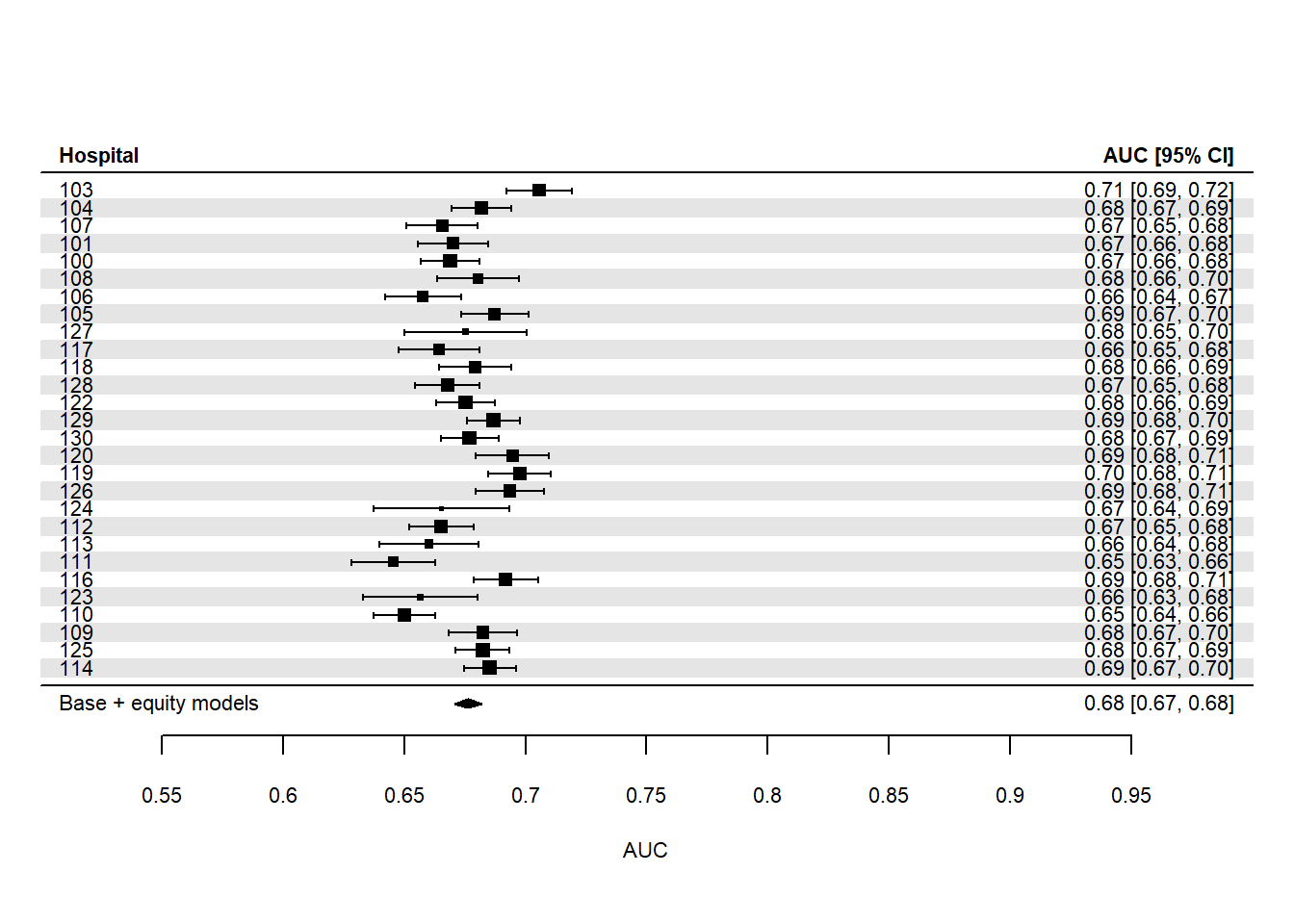

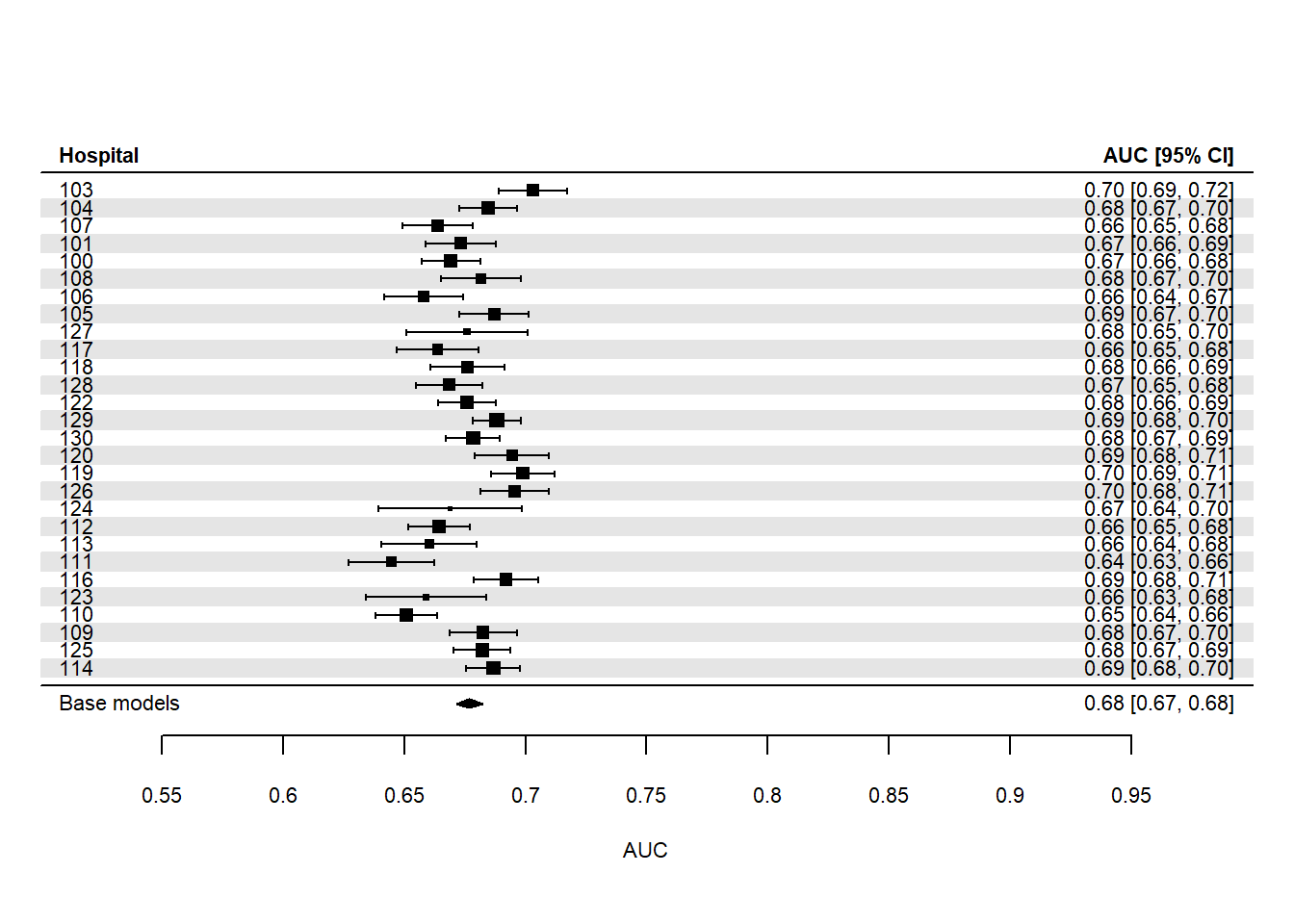


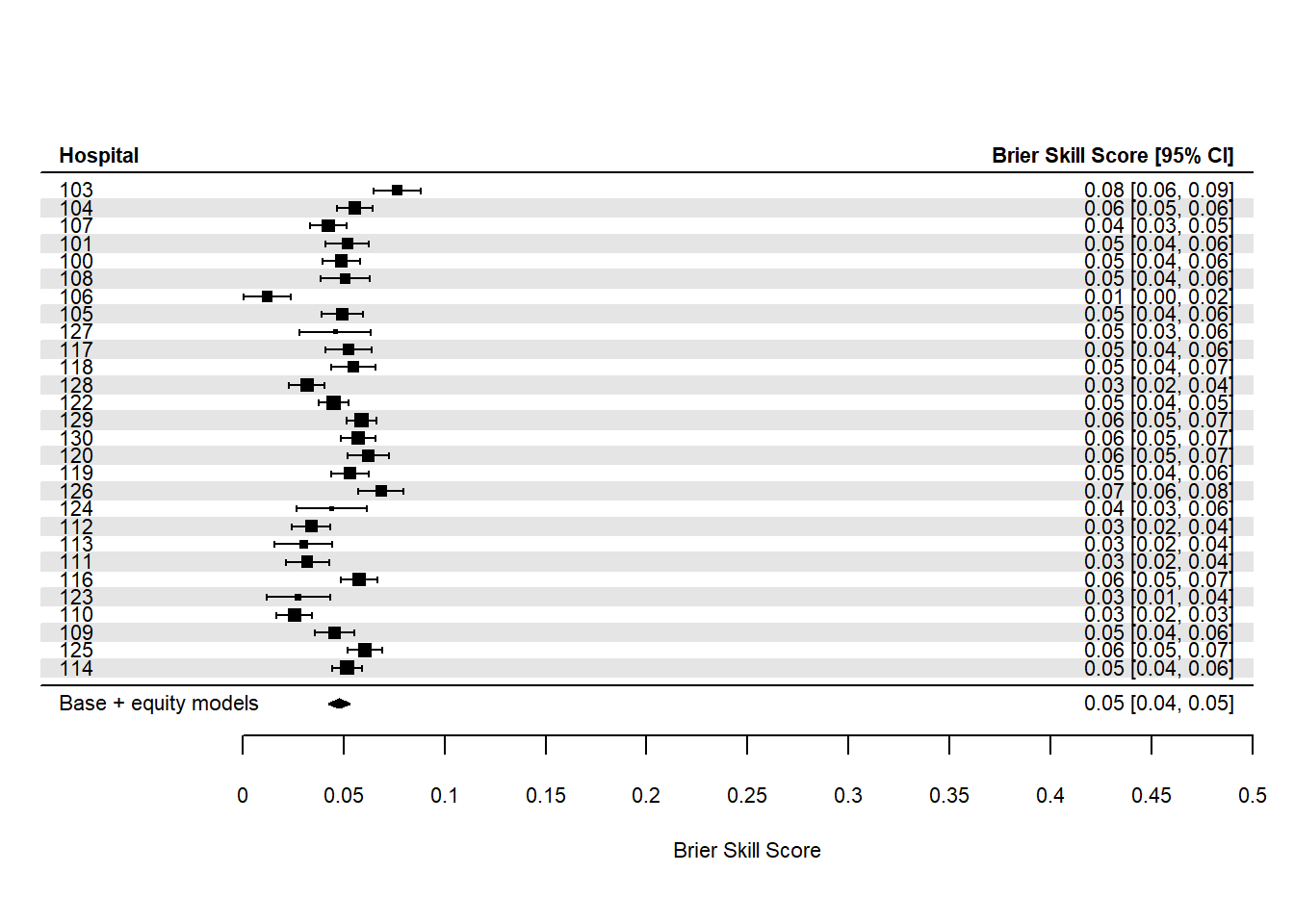

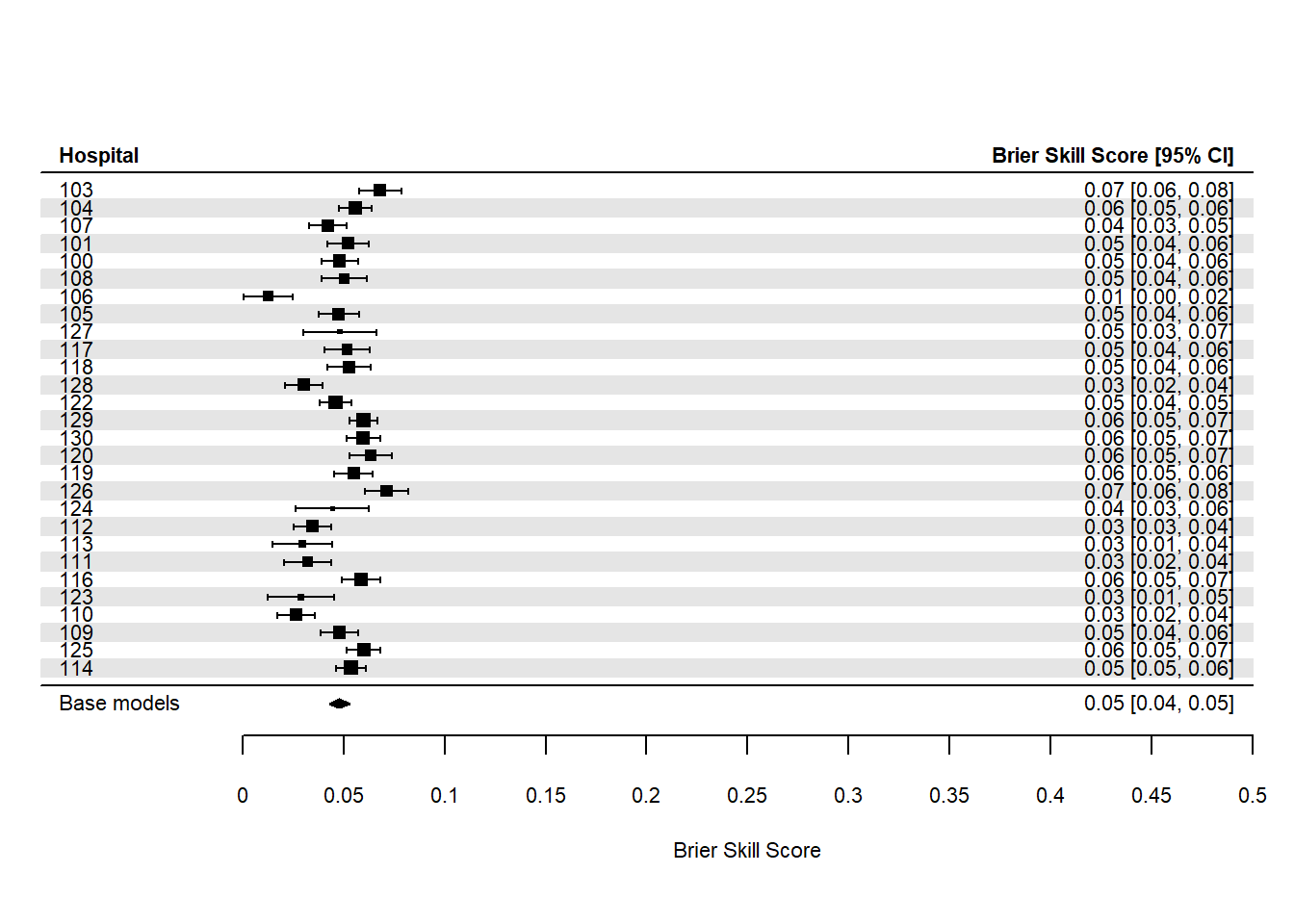


**Figure S2.2 continued: 30-day readmission: Forest plots of risk adjustment model performance metrics.**


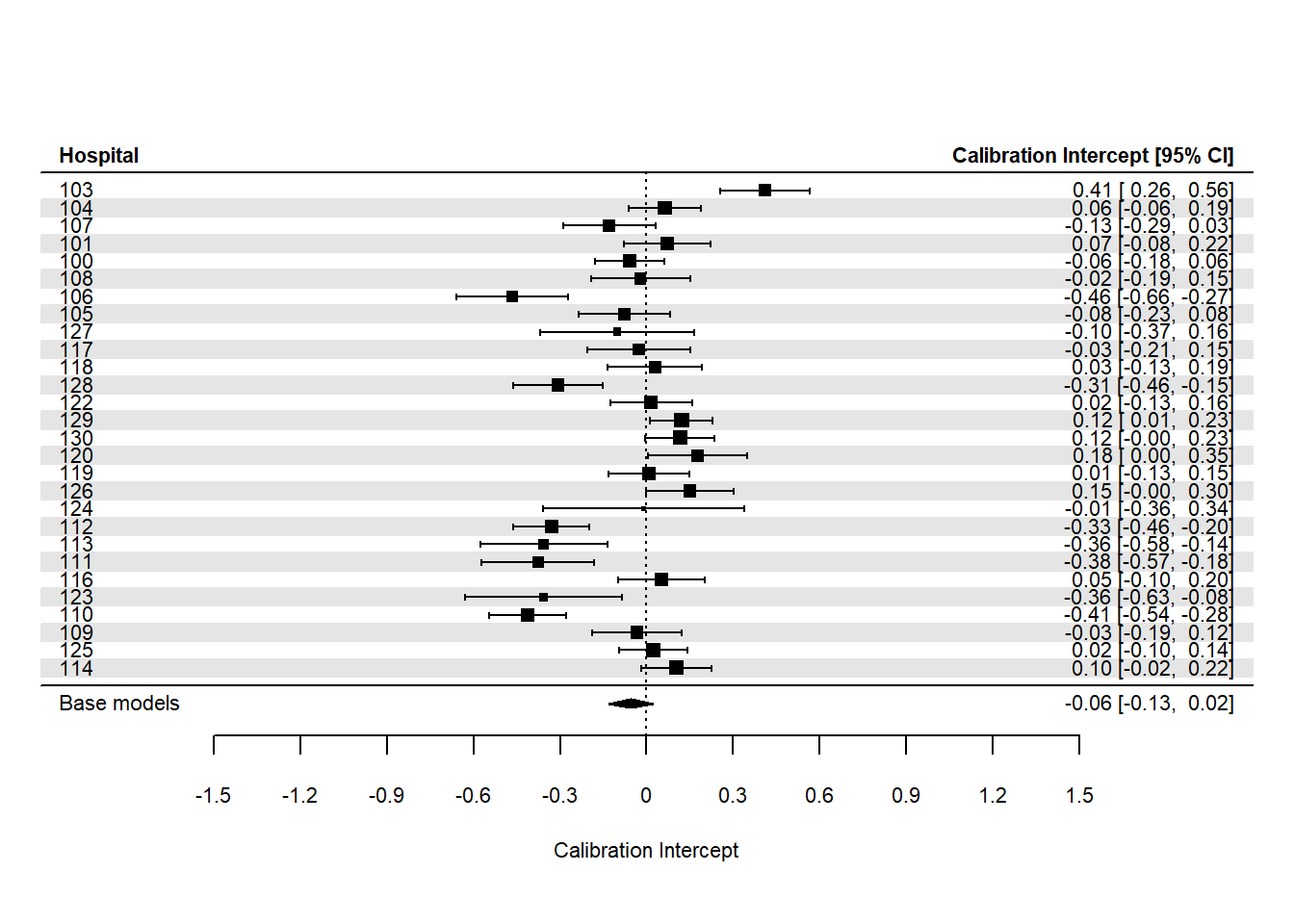

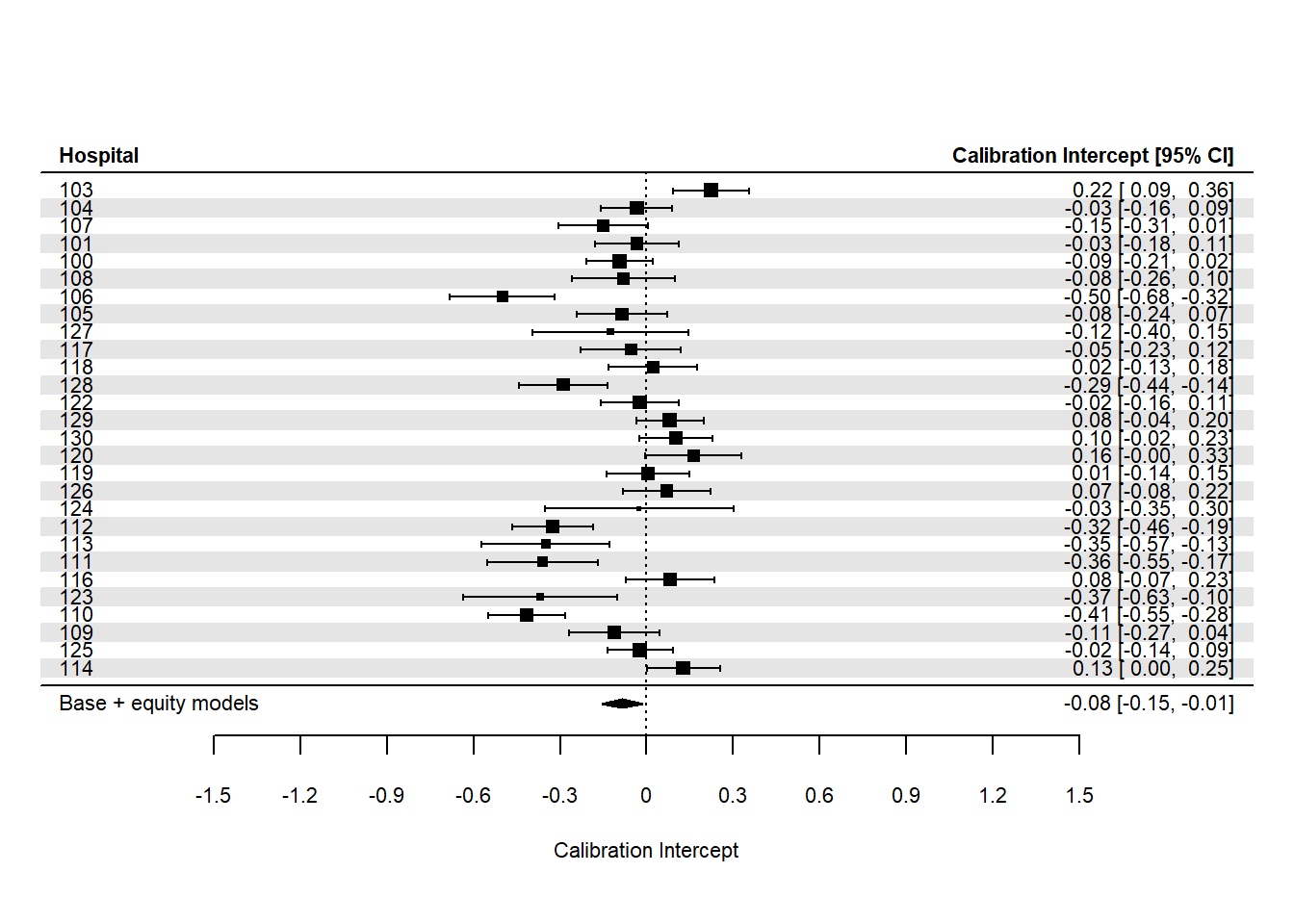


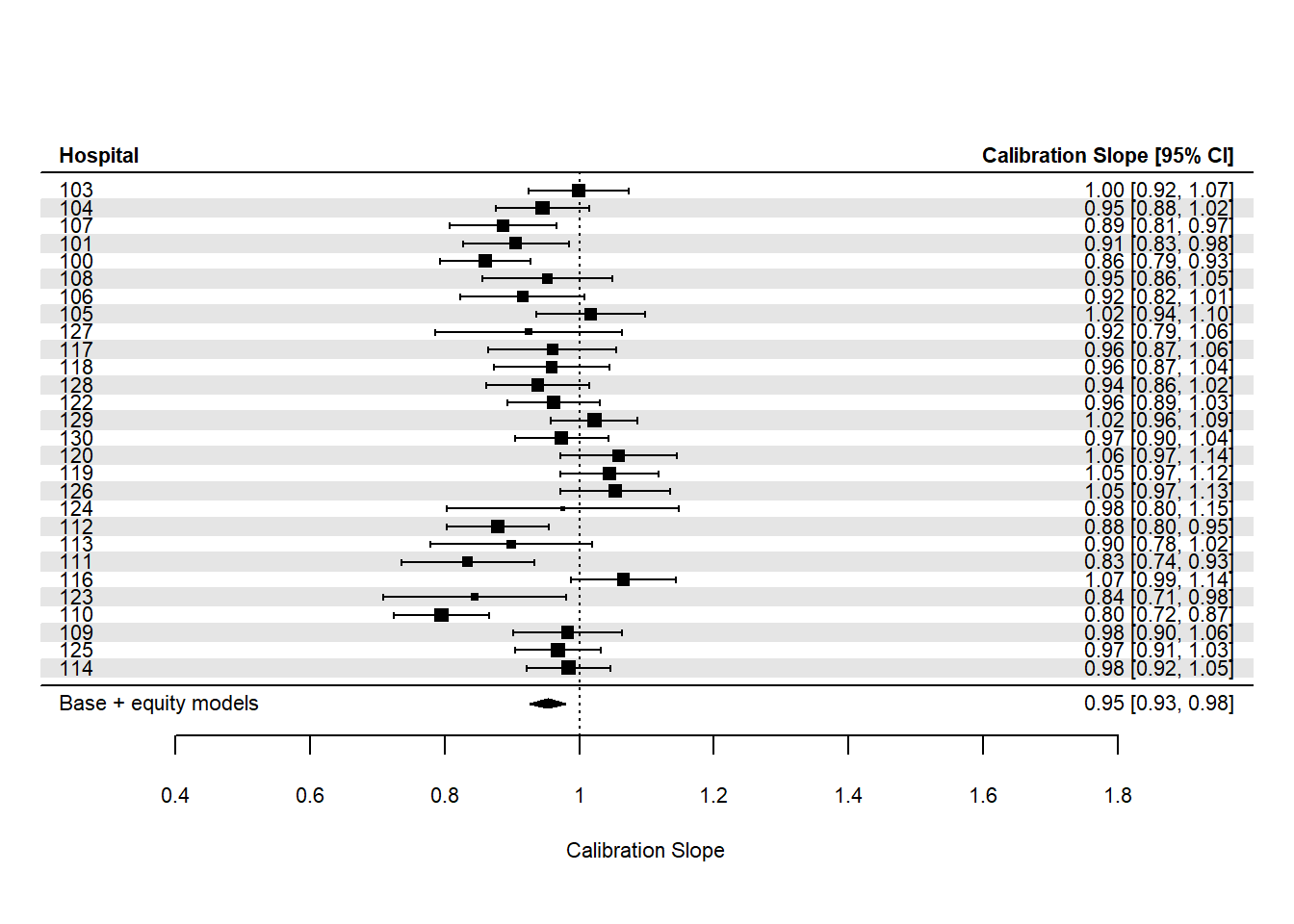

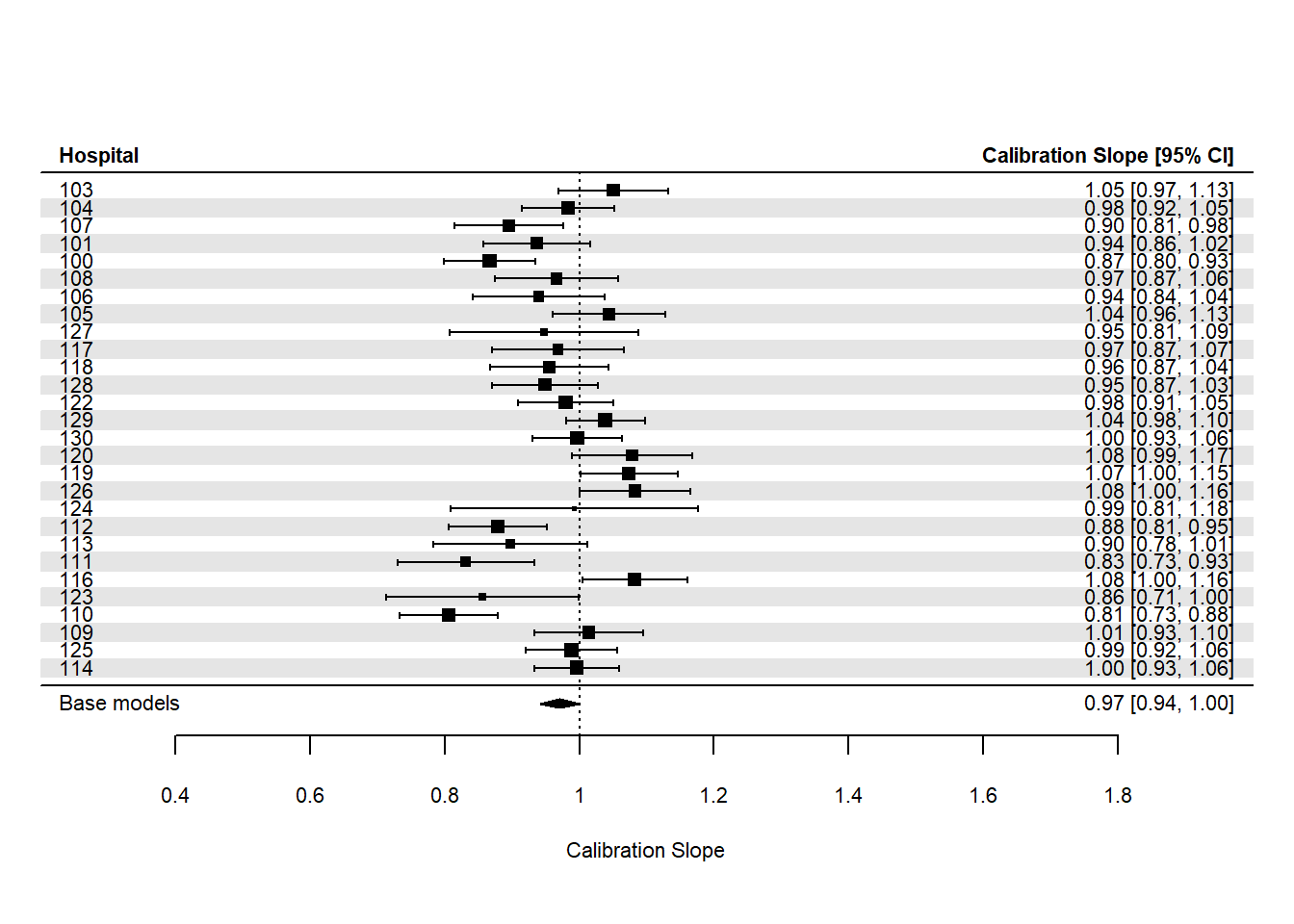


**Figure S2.2 continued: 30-day readmission: Forest plots of risk adjustment model performance metrics.**


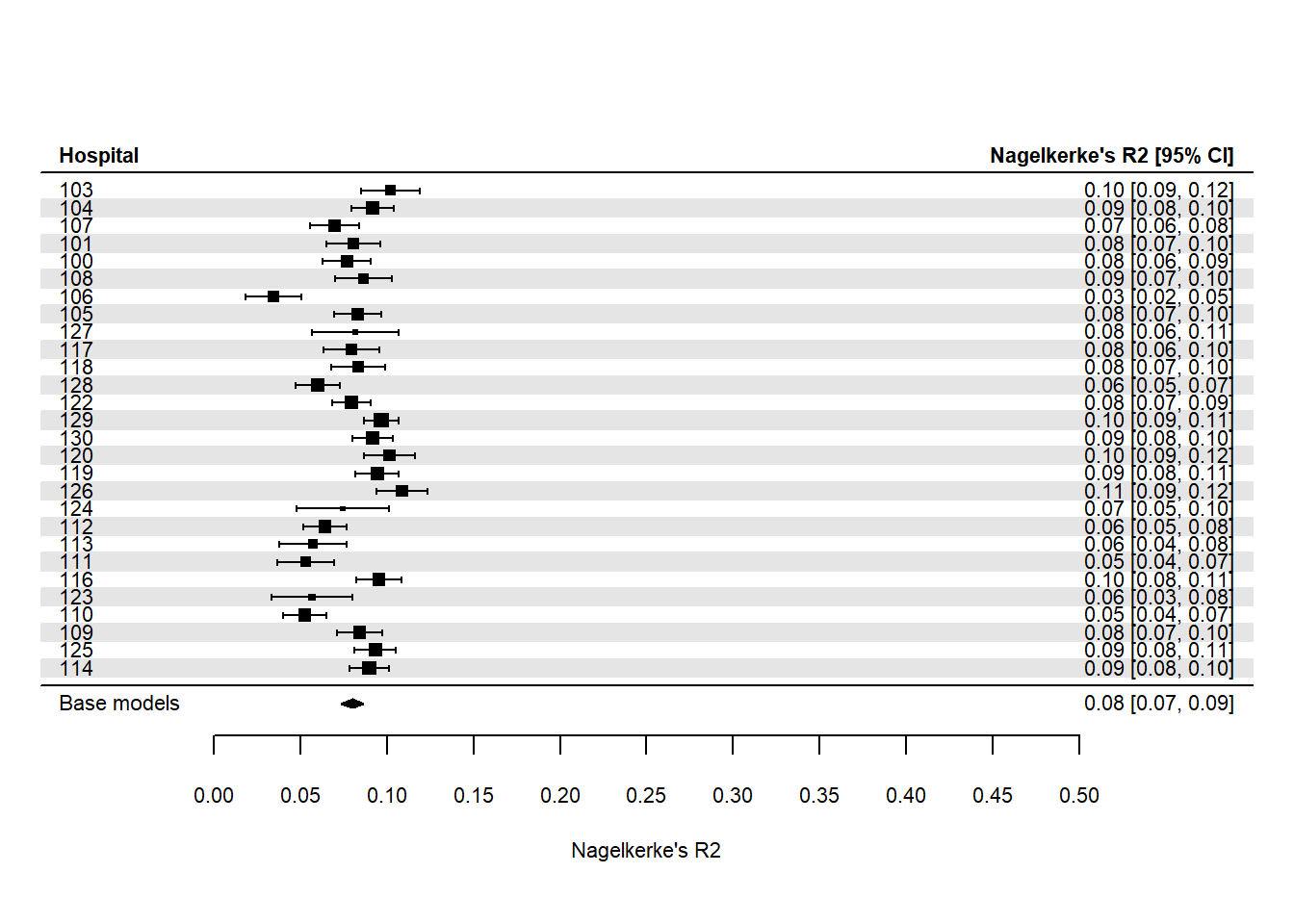

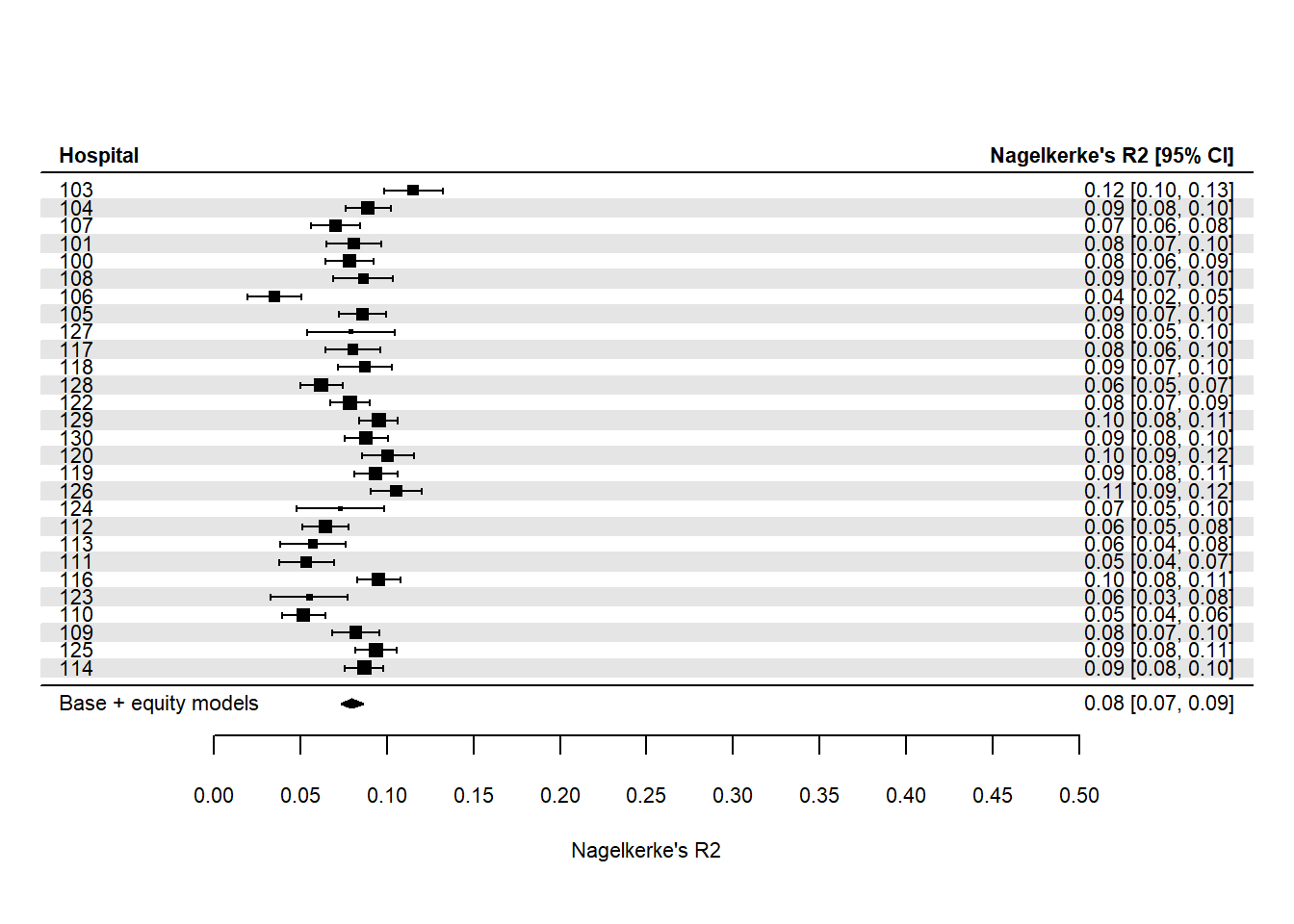

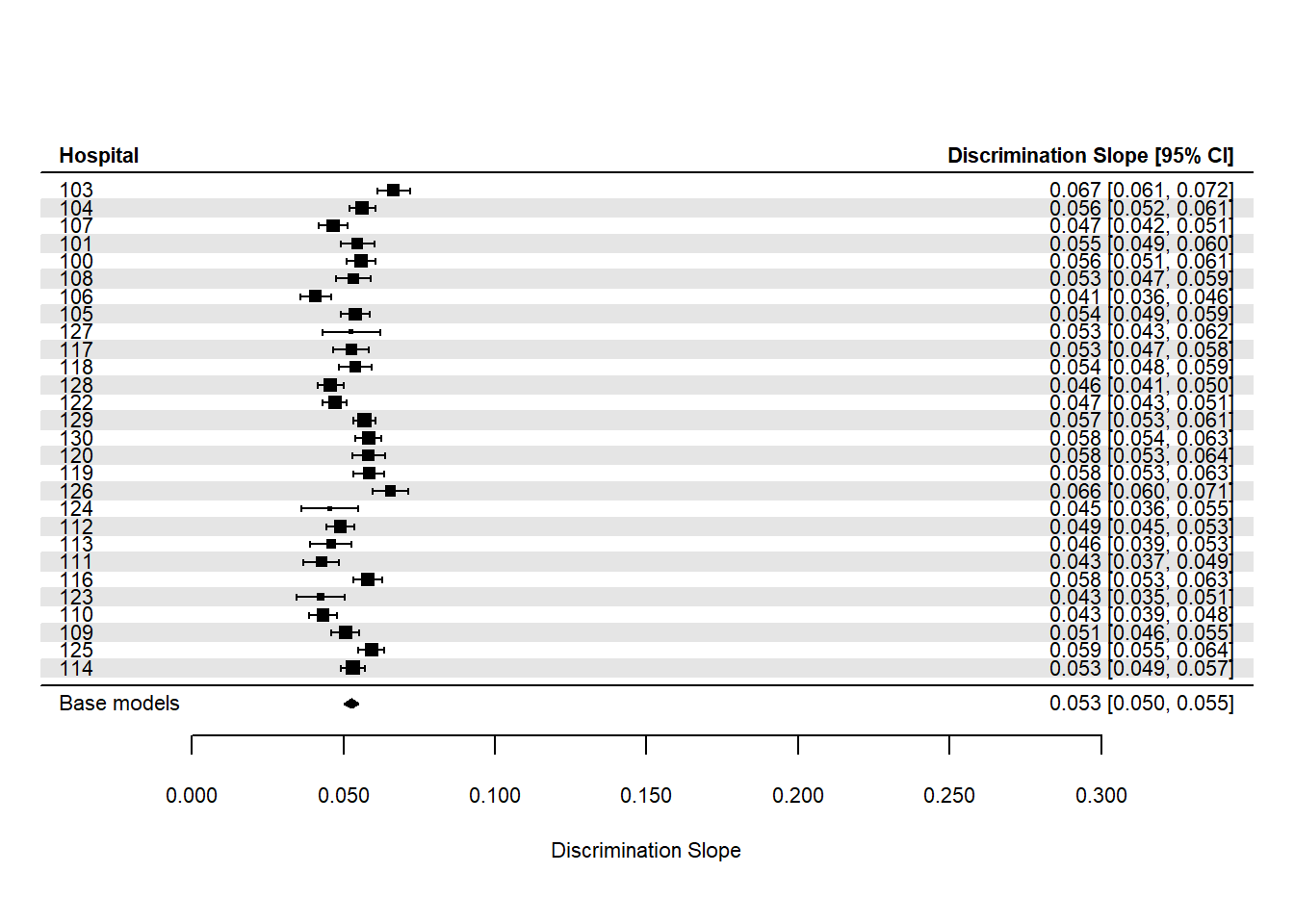

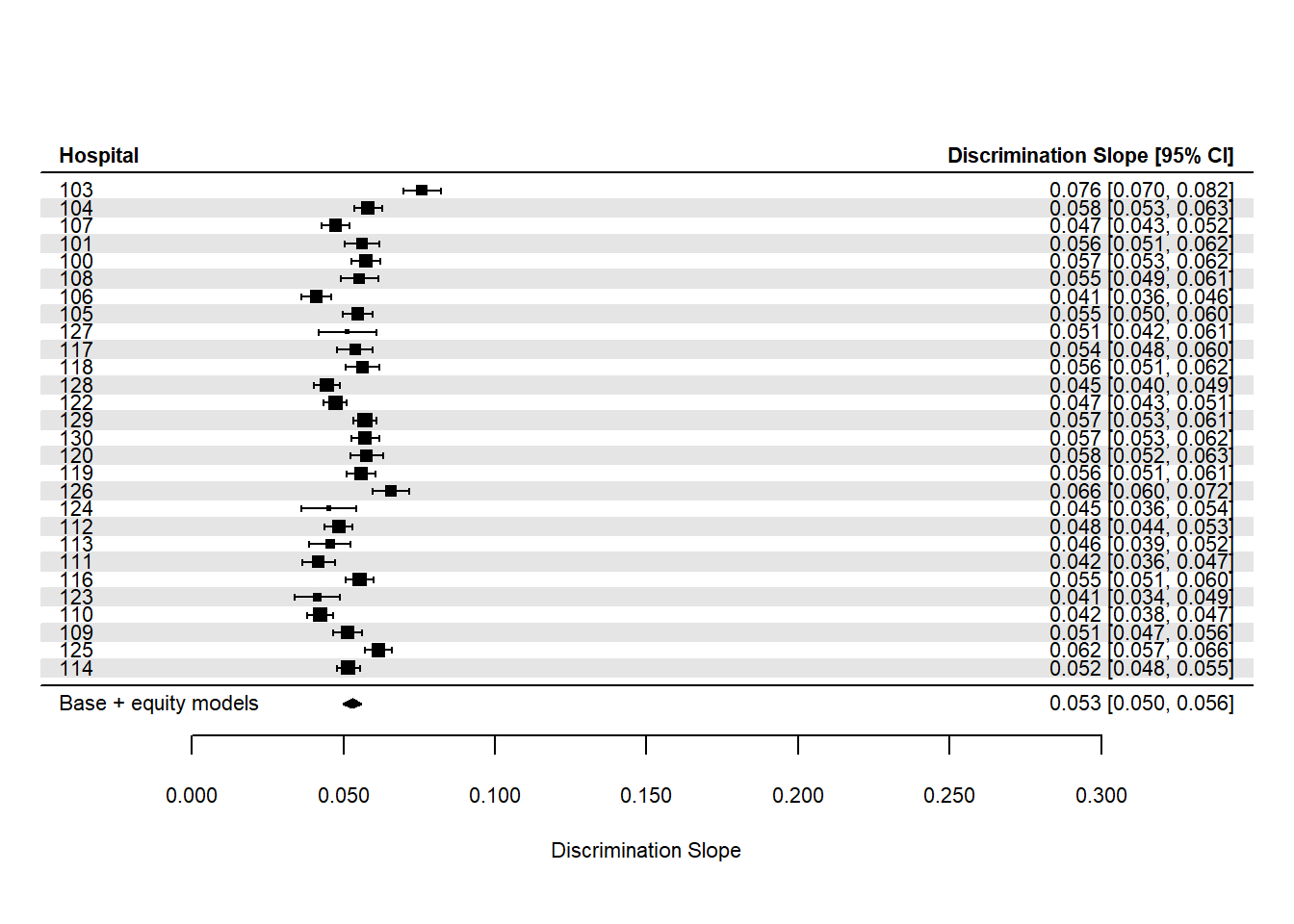


**Figure S2.2 continued: 30-day readmission: Forest plots of risk adjustment model performance metrics.**


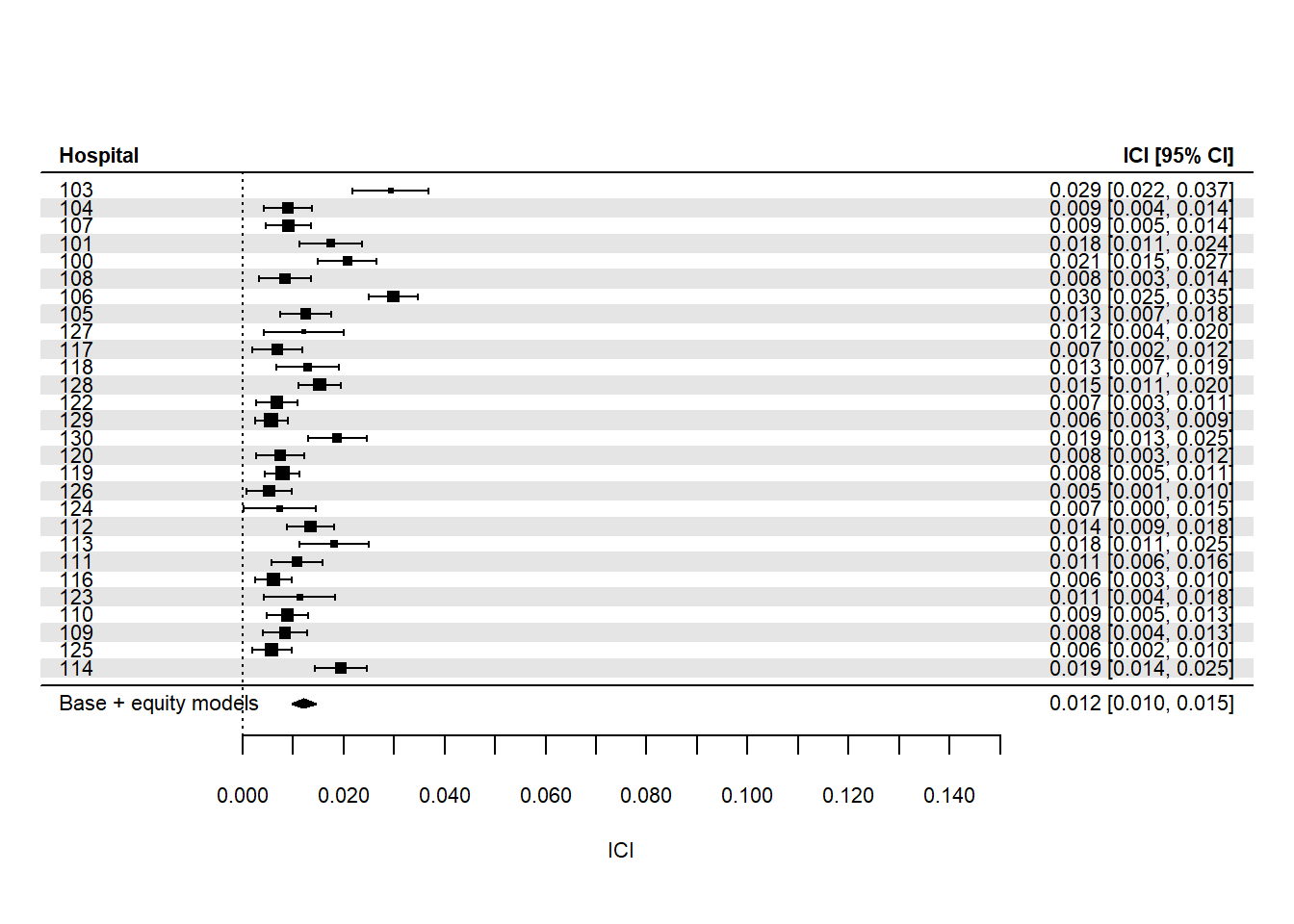

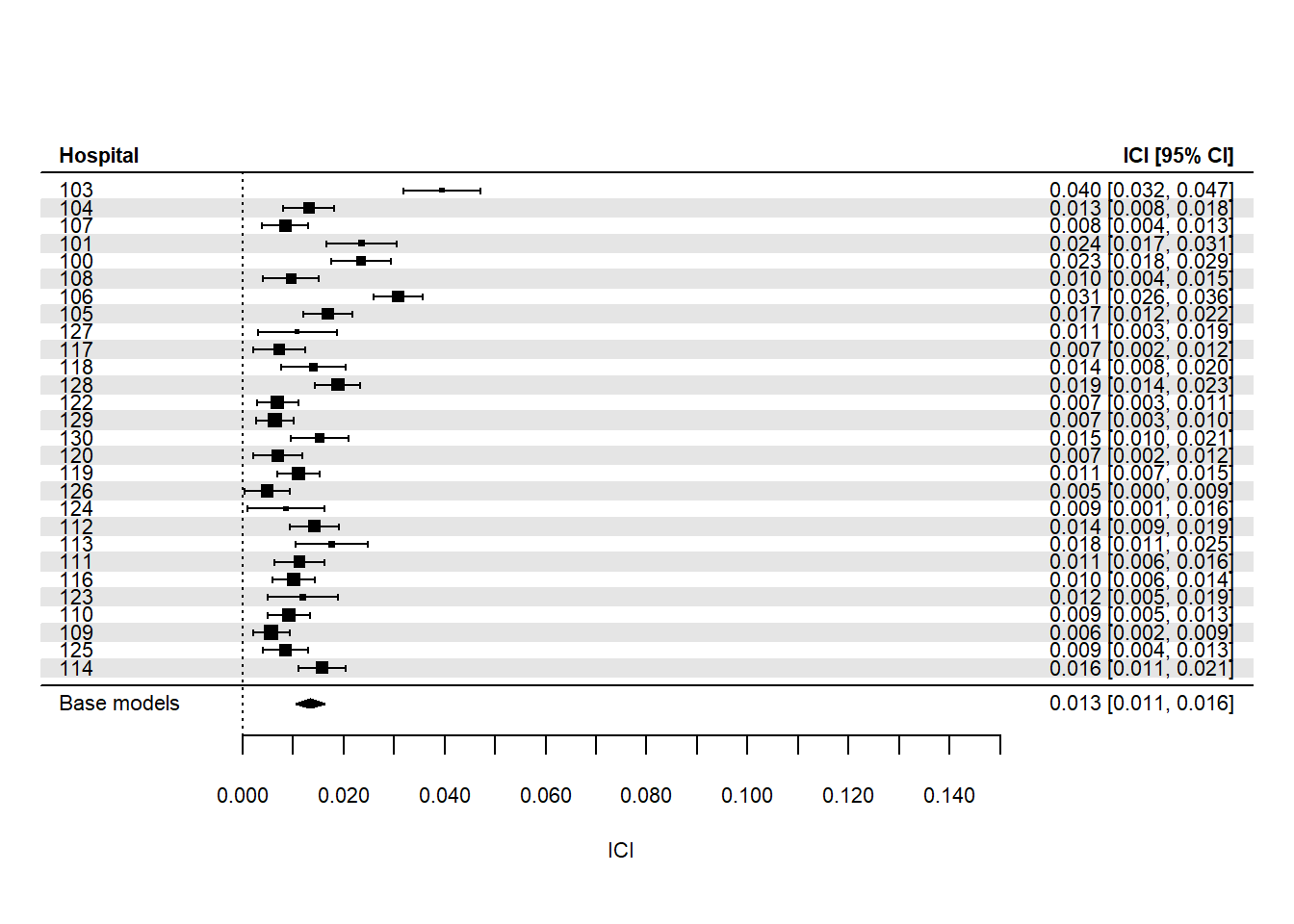

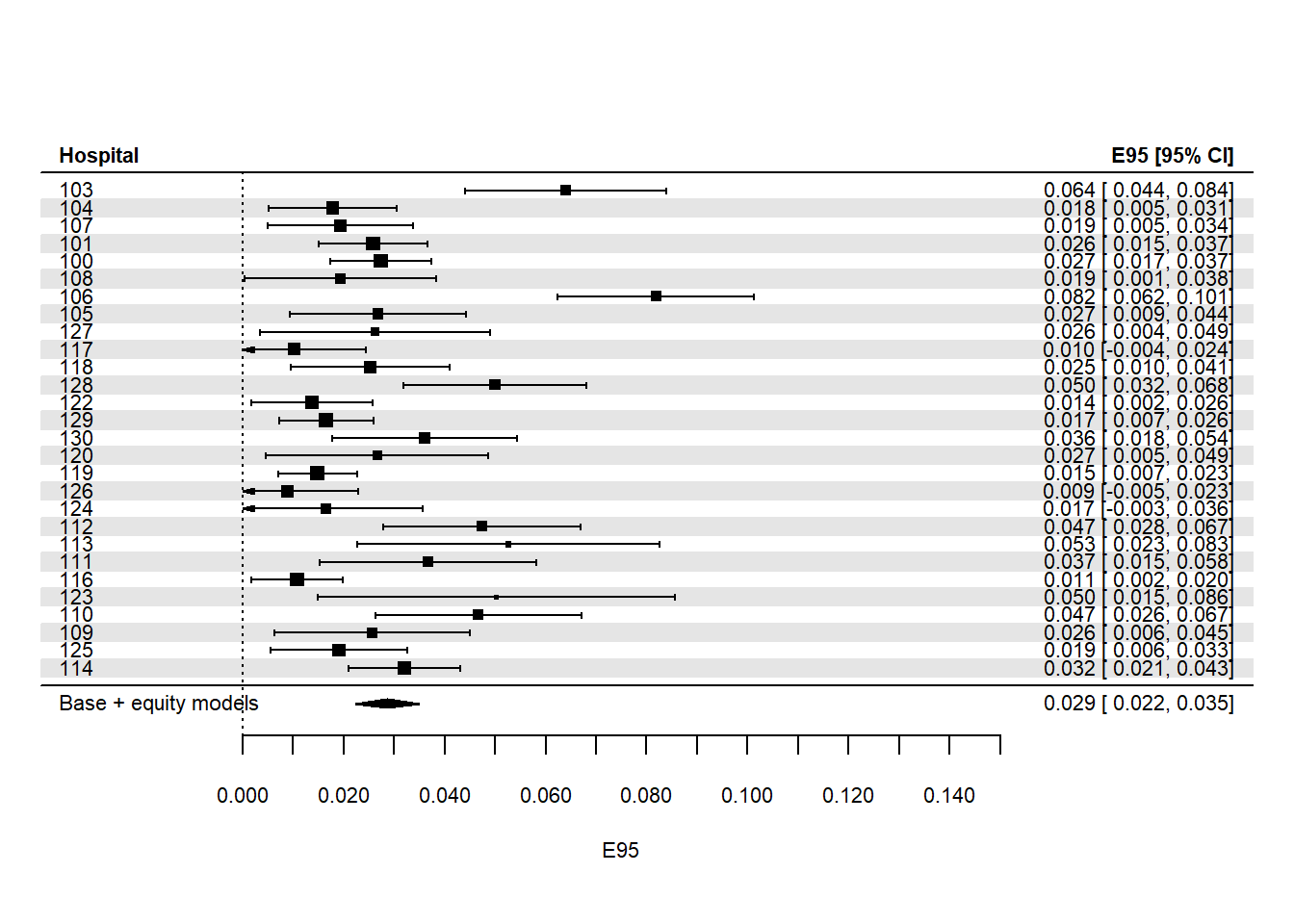

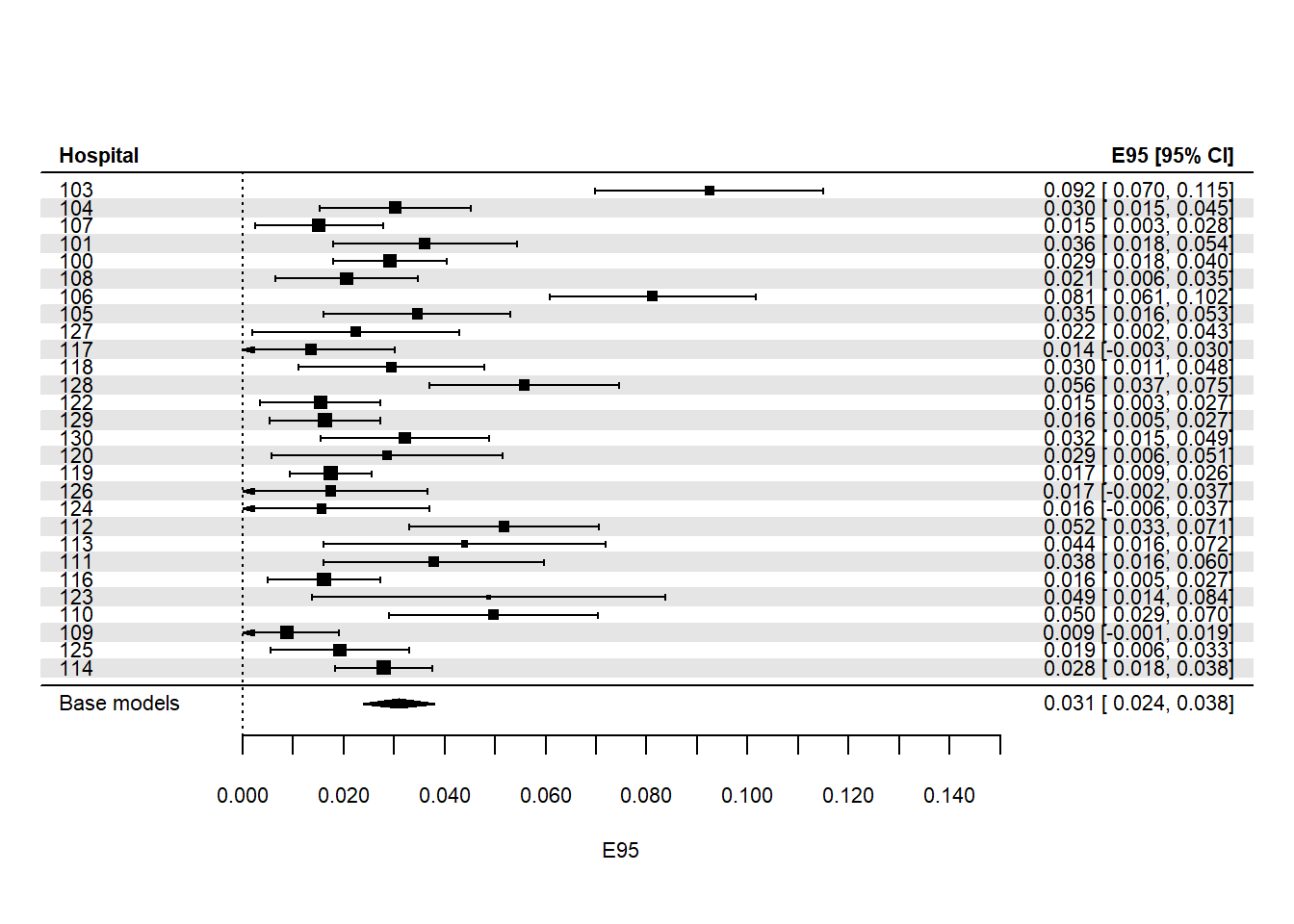


### Figure S2.3: 7-day readmission: Forest plots of risk adjustment model performance metrics.

*Plots on the left are without equity-related adjustment, plots on the right are with equity-related adjustment*

Metrics were obtained through internal-external cross-validation where each hospital is held out separately, models are re-fit in the remaining 27 hospitals, and model performance is assessed in the held-out hospital.
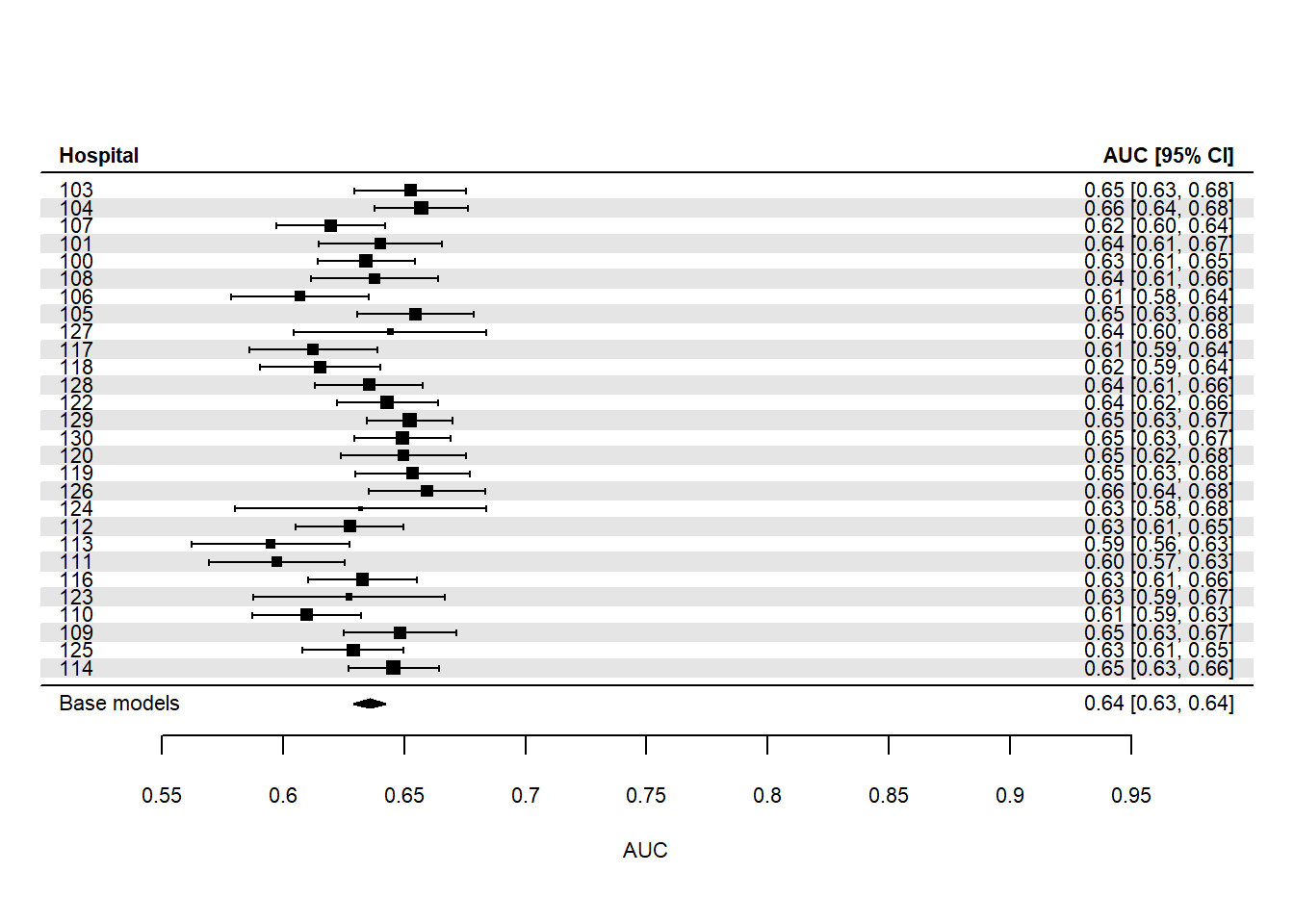

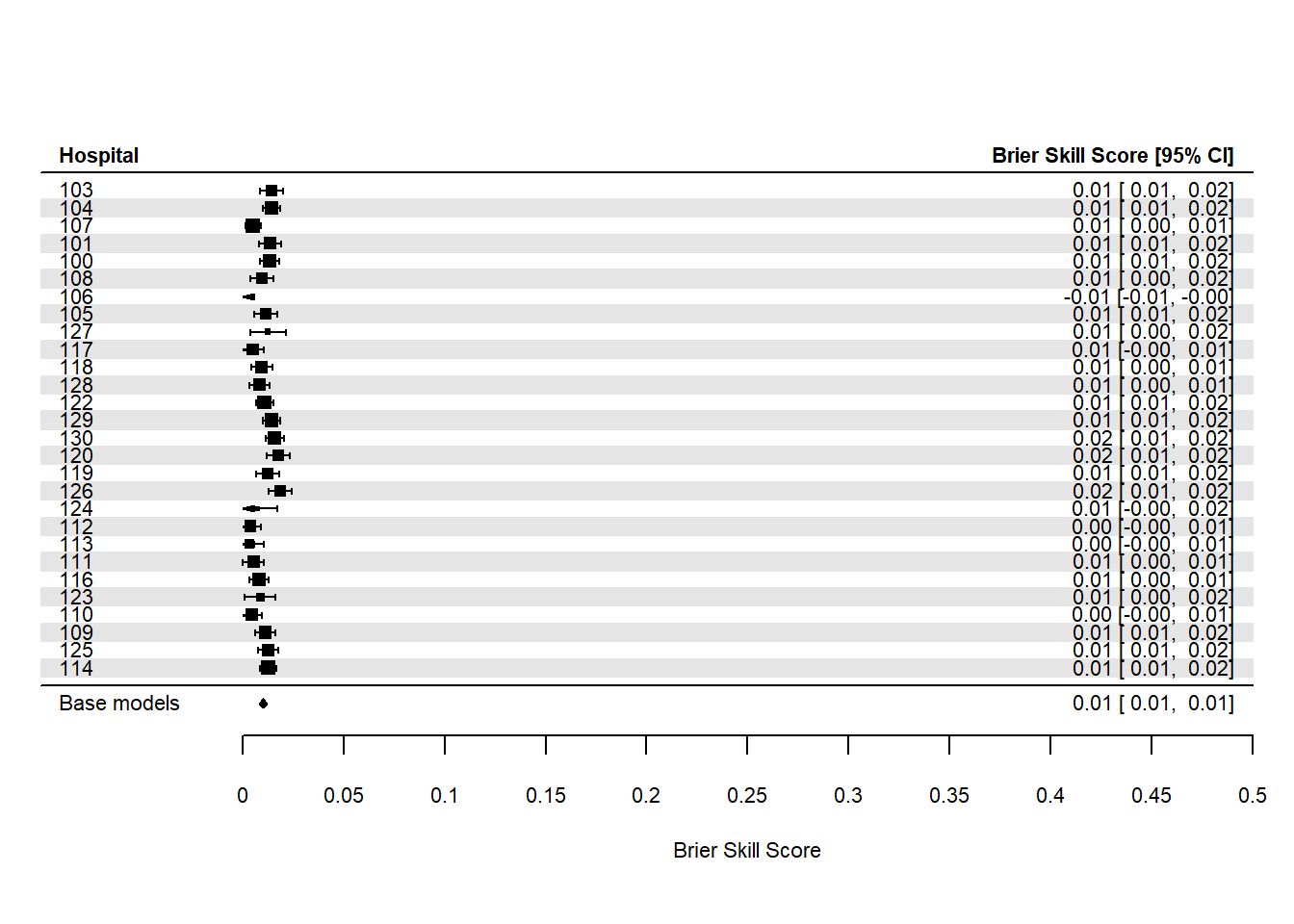

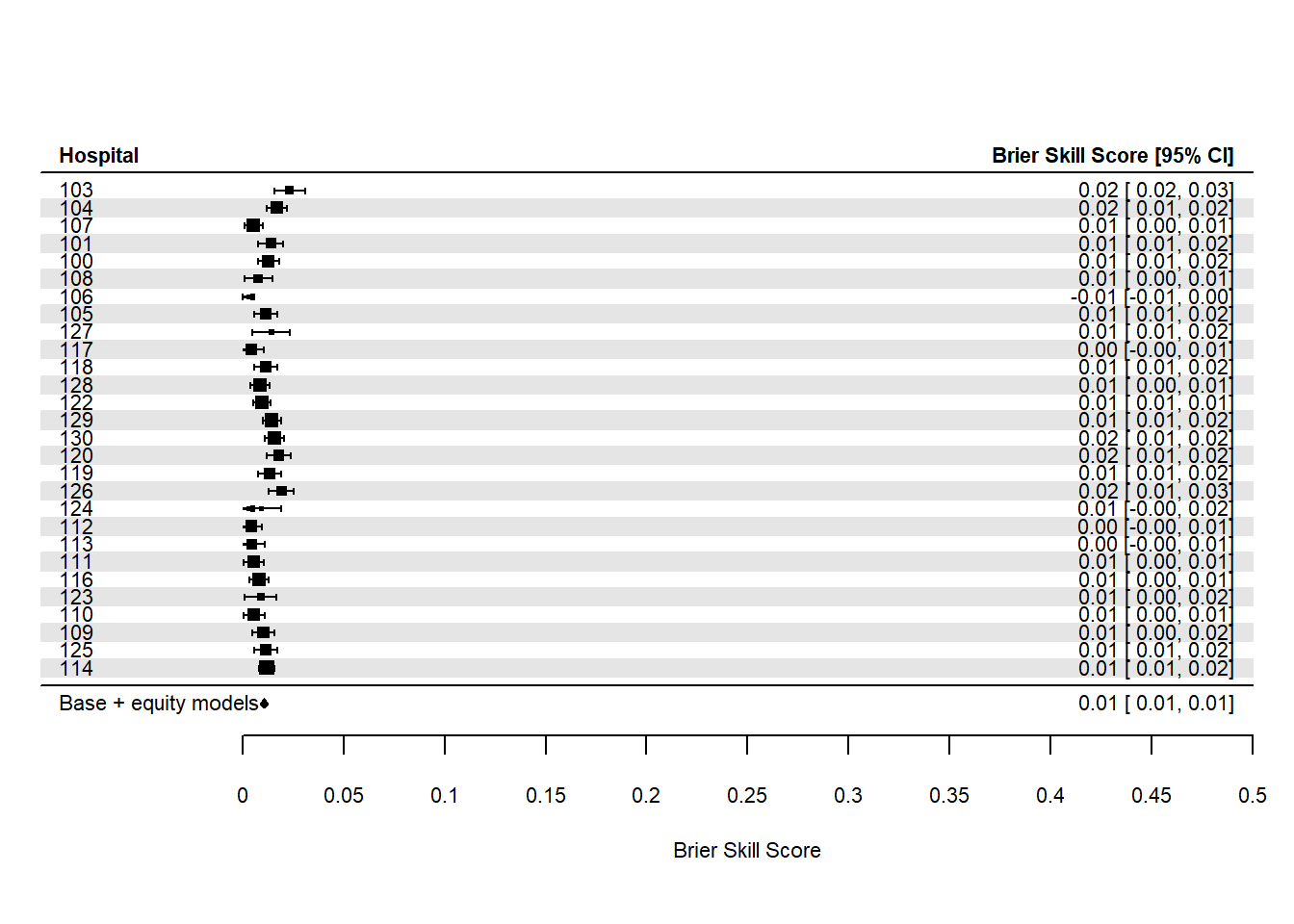

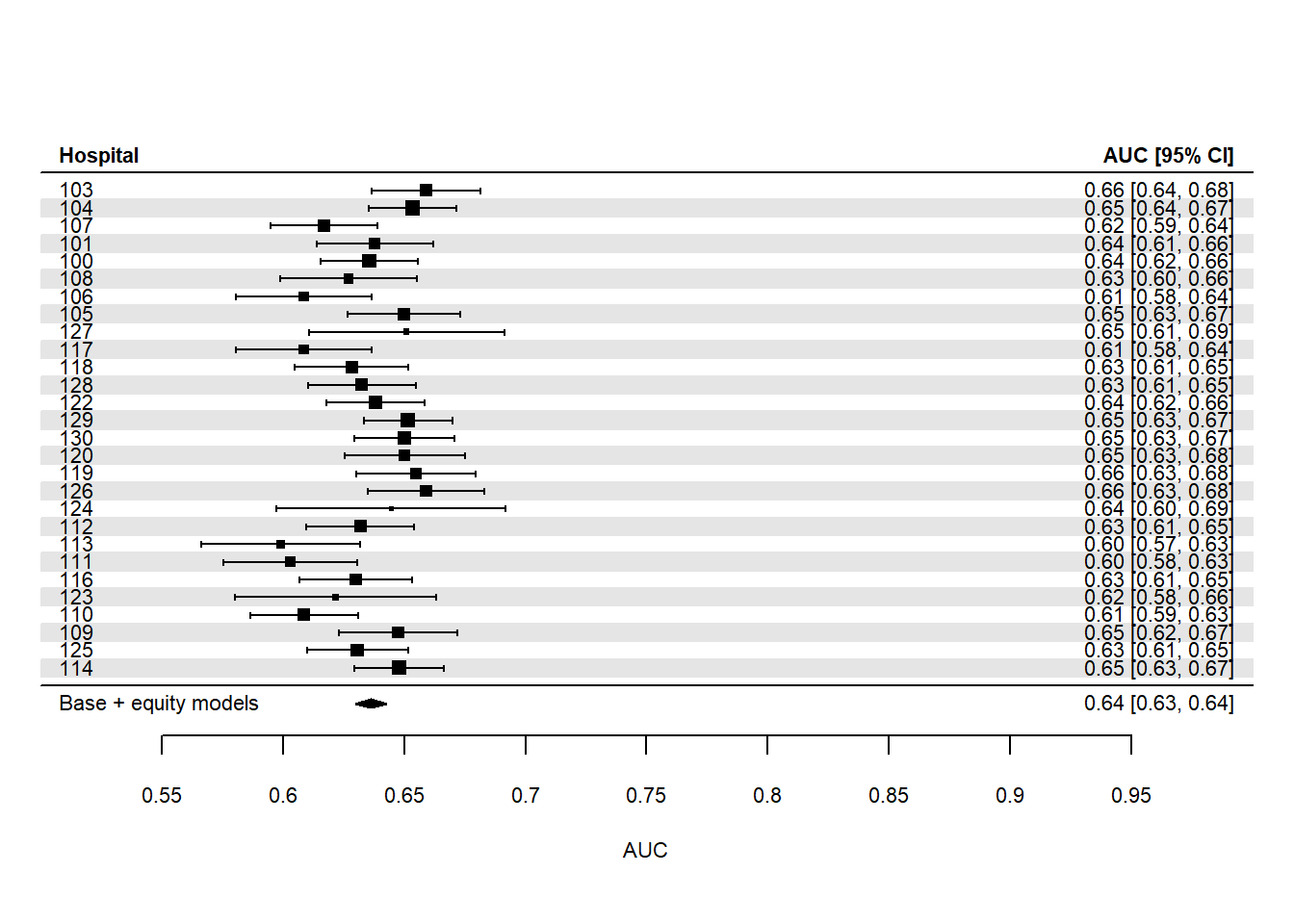


**Figure S2.3 continued: 7-day readmission: Forest plots of risk adjustment model performance metrics.**


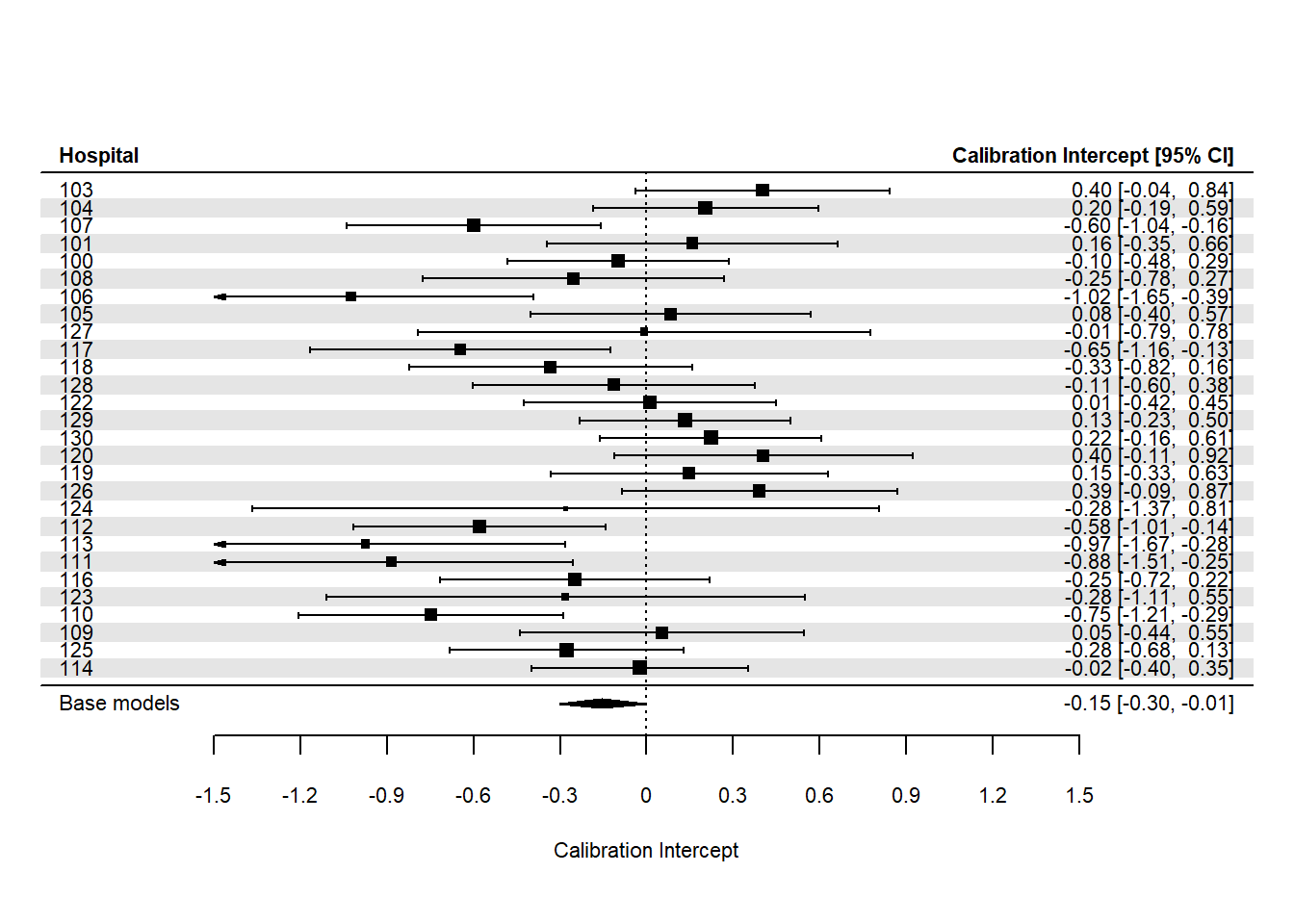

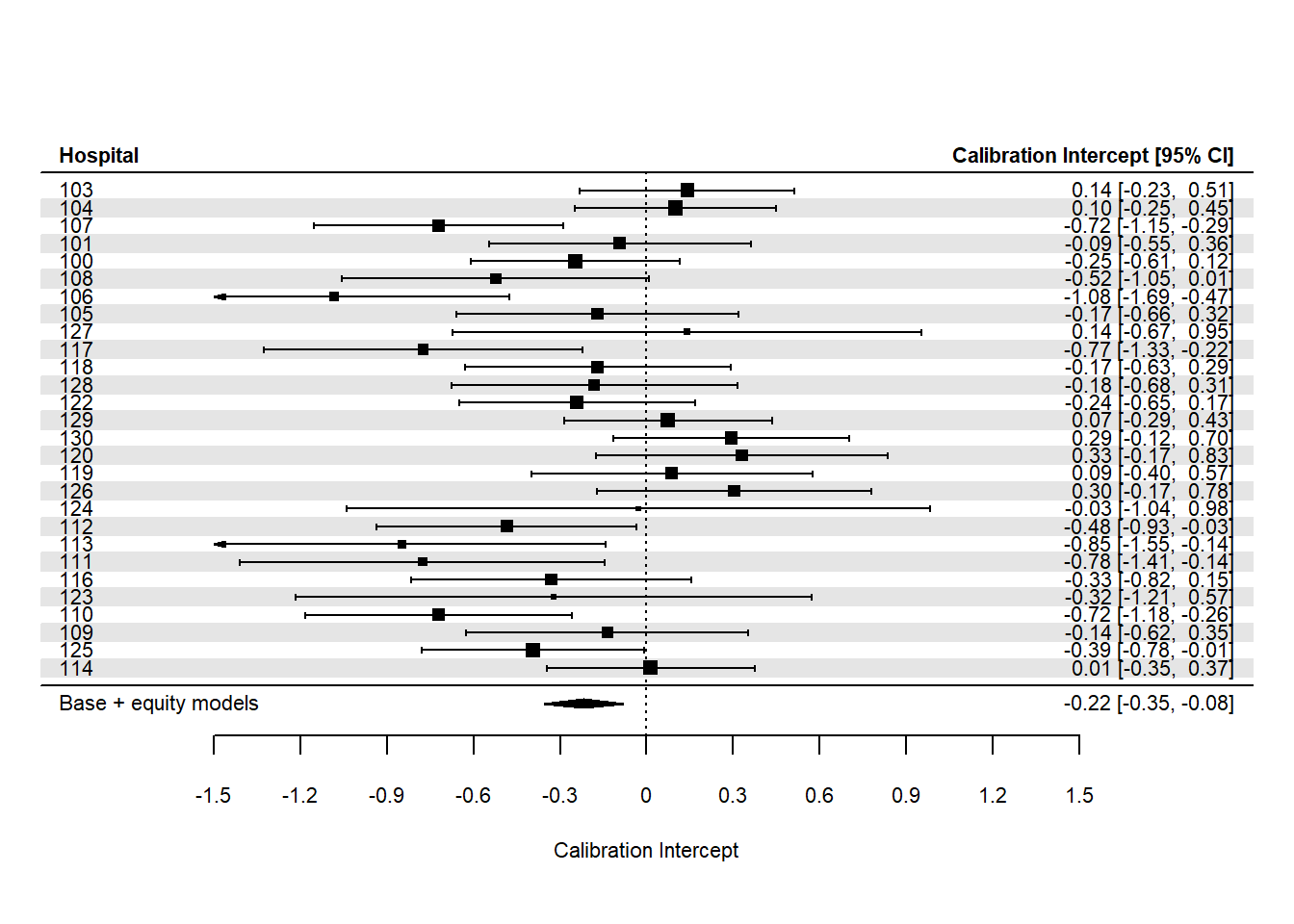

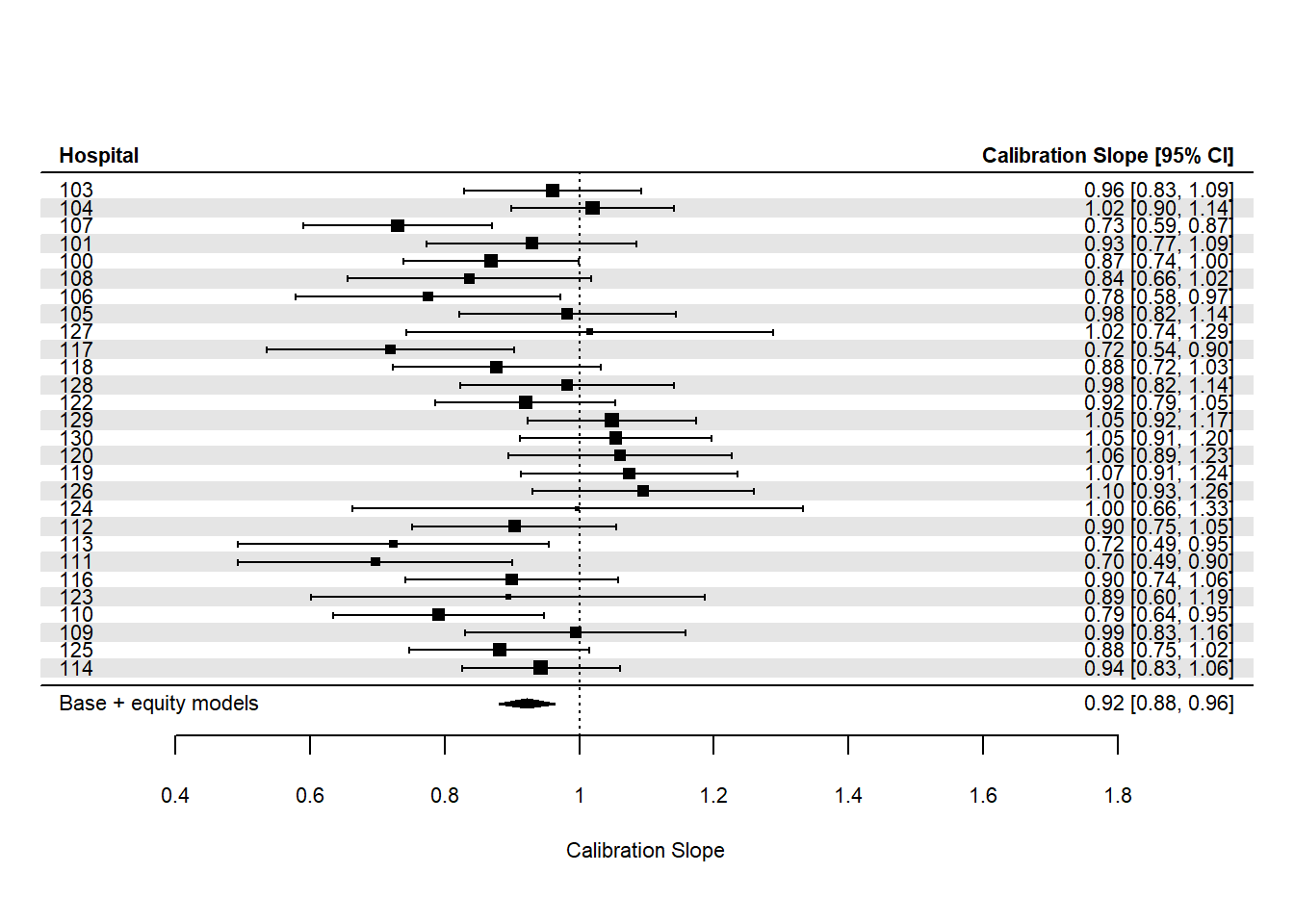

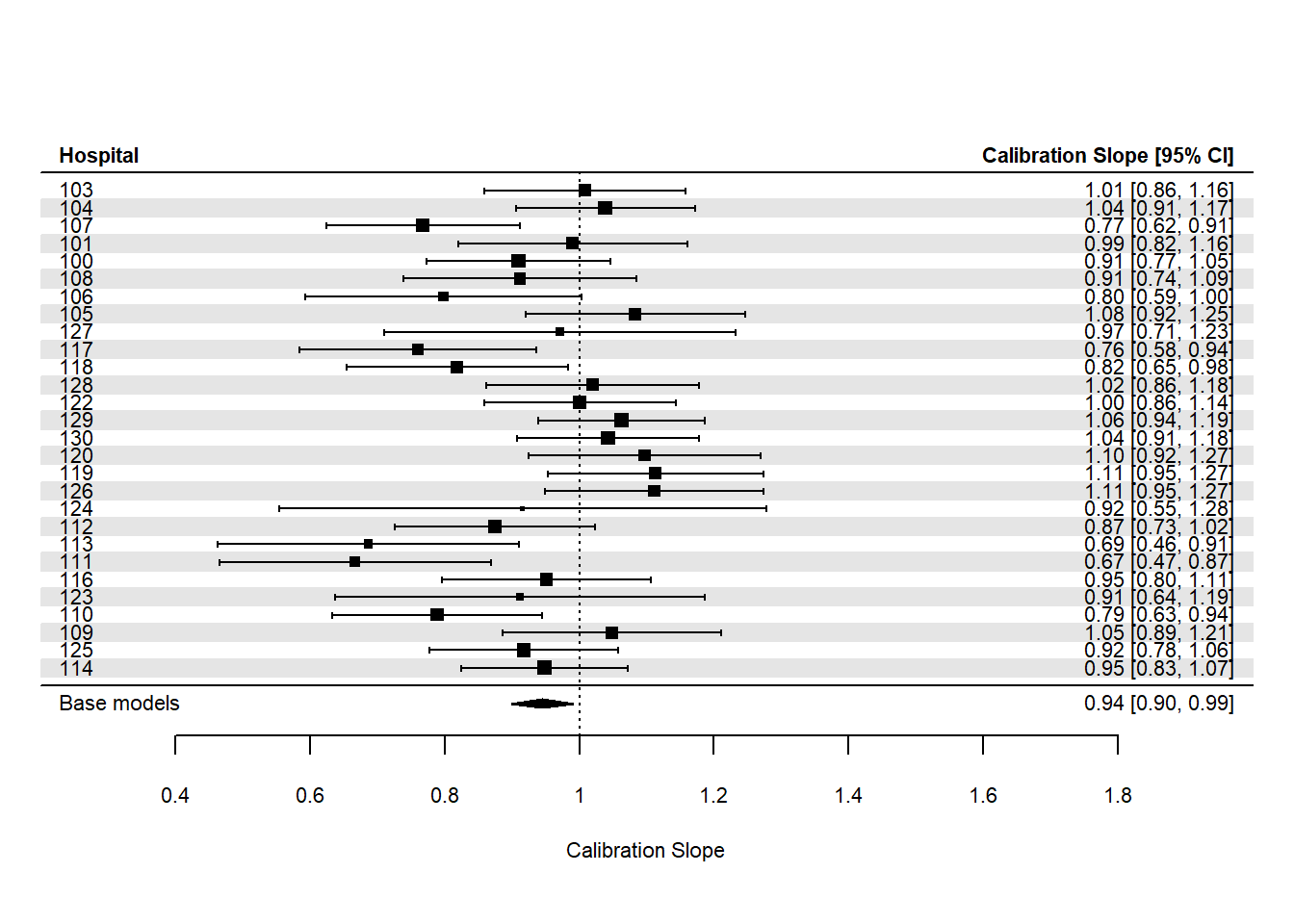


**Figure S2.3 continued: 7-day readmission: Forest plots of risk adjustment model performance metrics.**
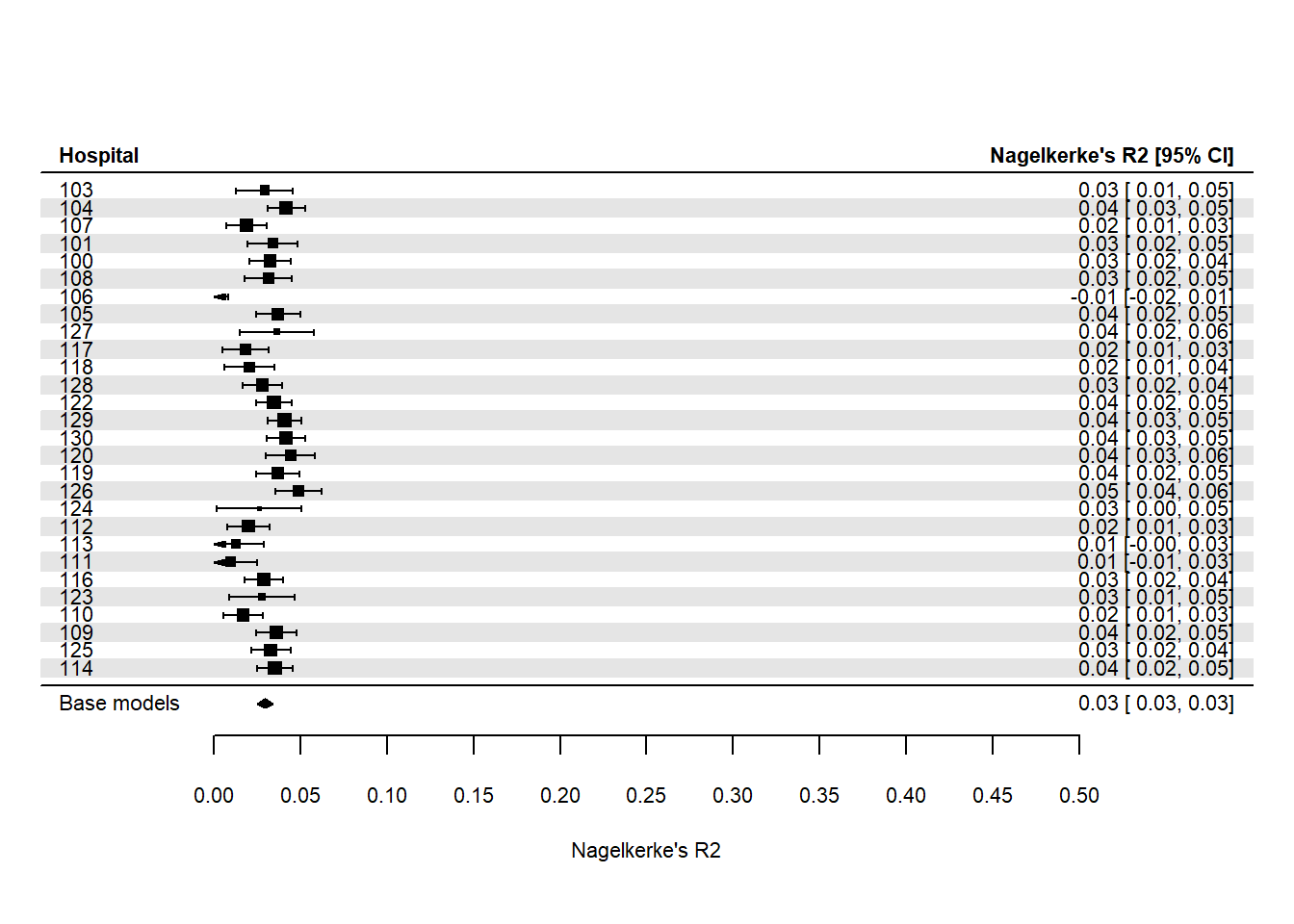

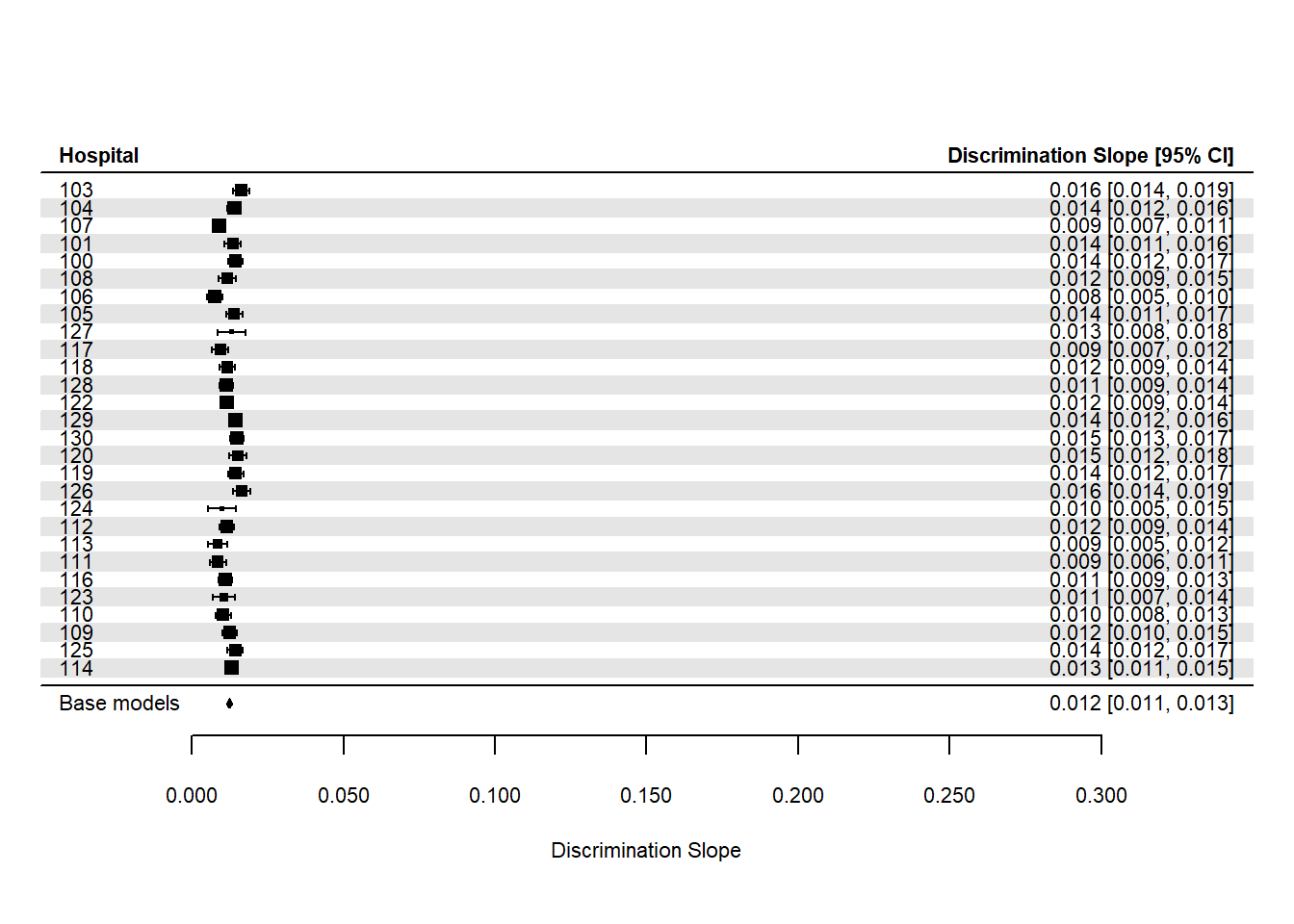

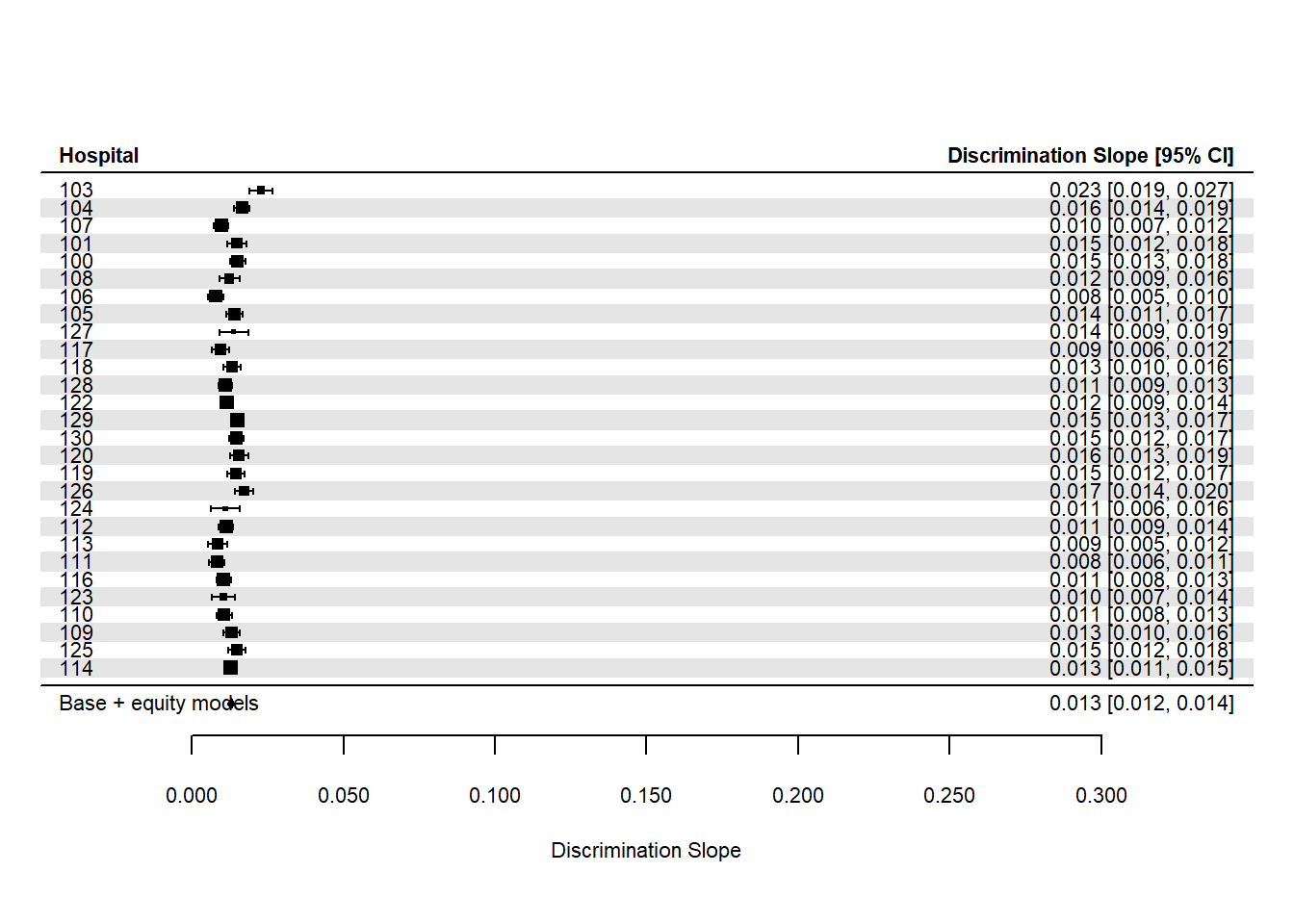

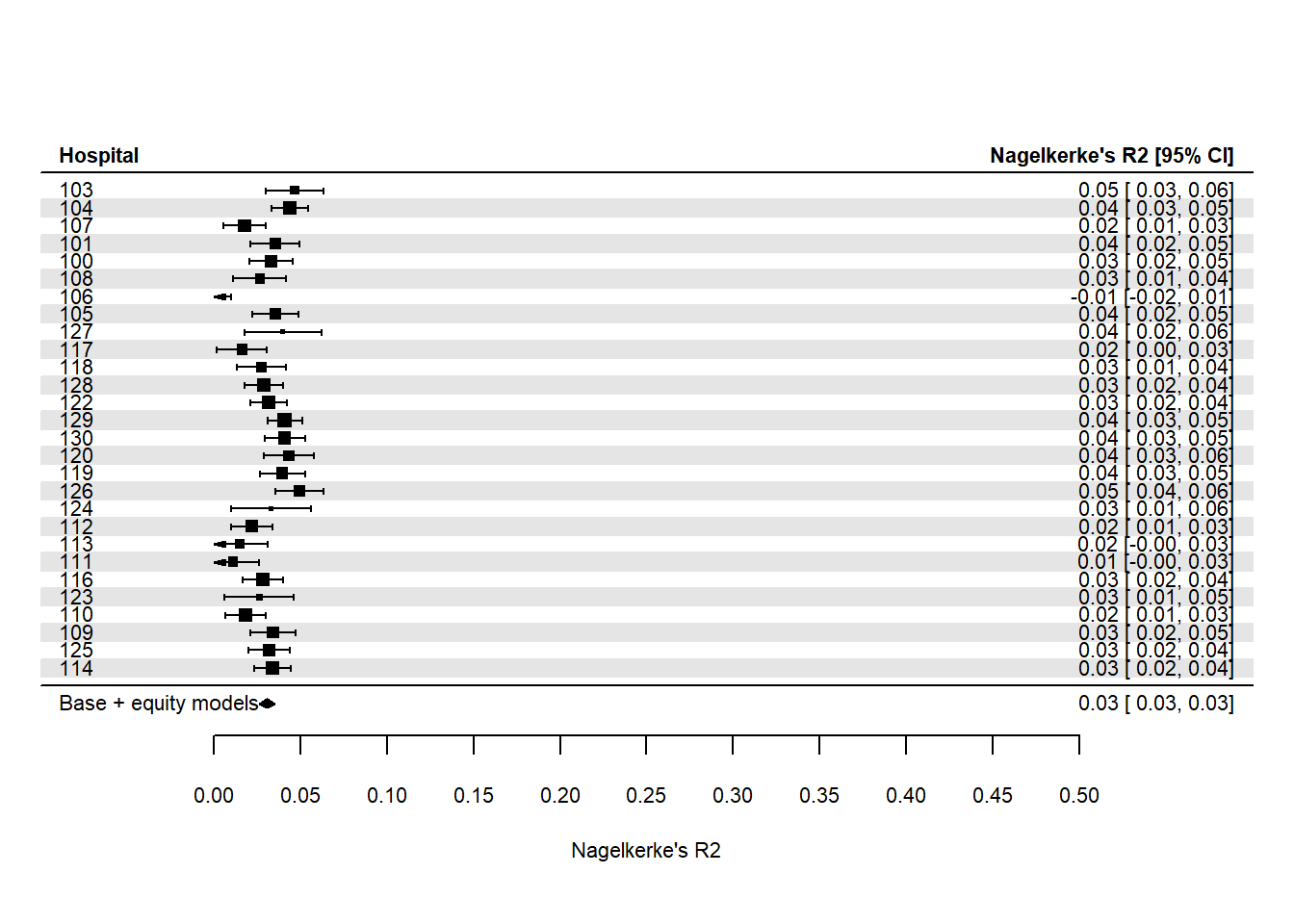


**Figure S2.3 continued: 7-day readmission: Forest plots of risk adjustment model performance metrics.**


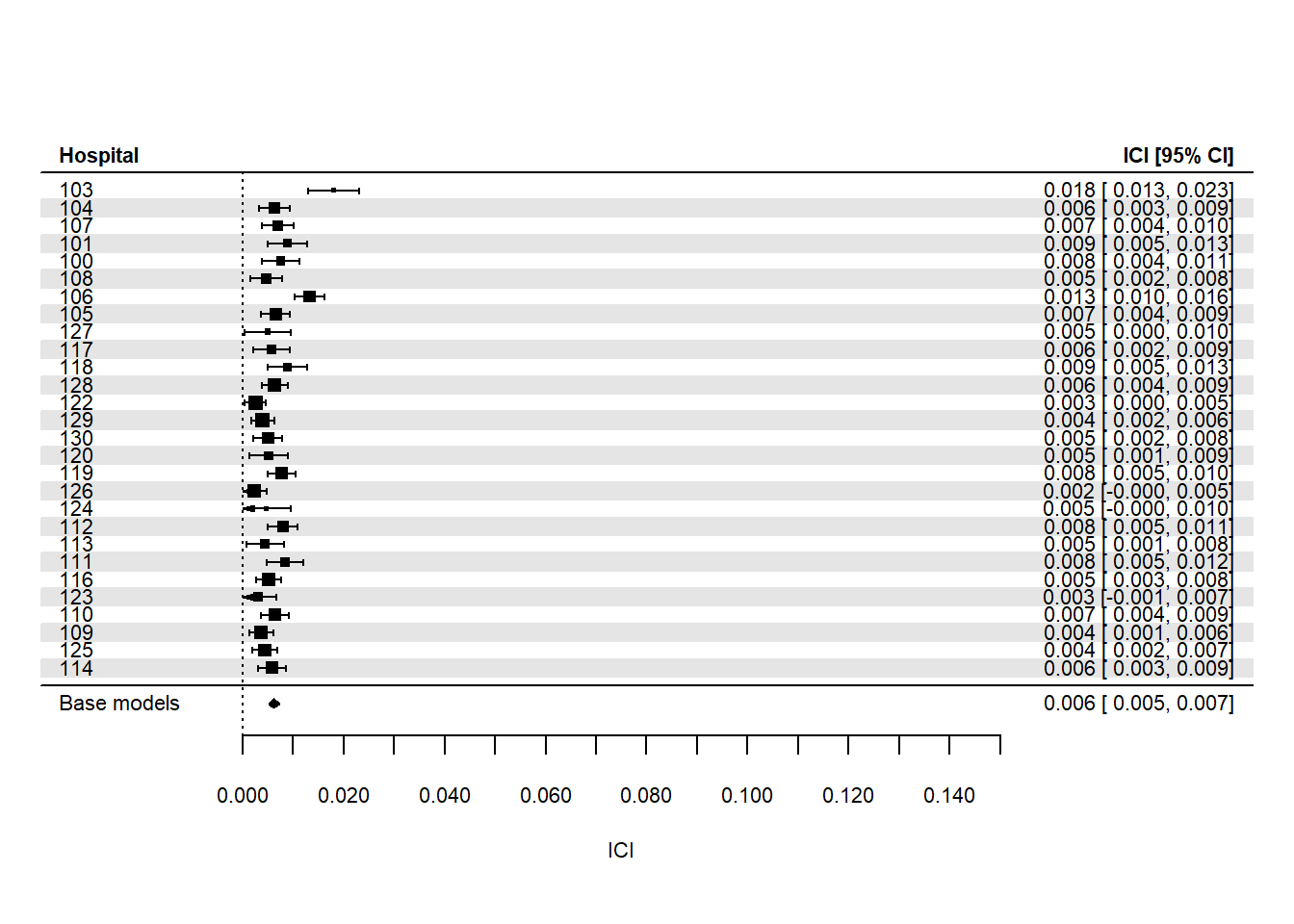

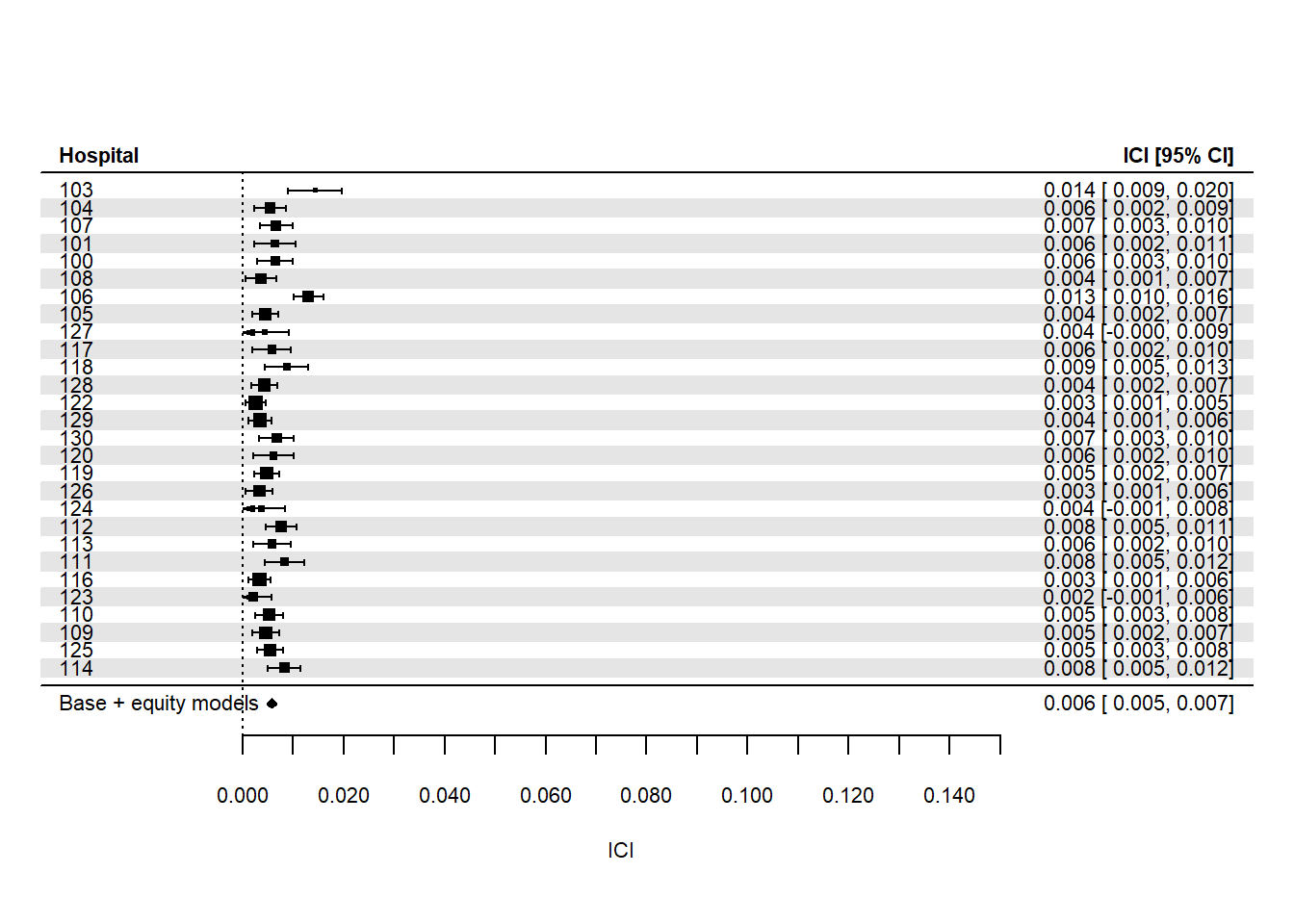

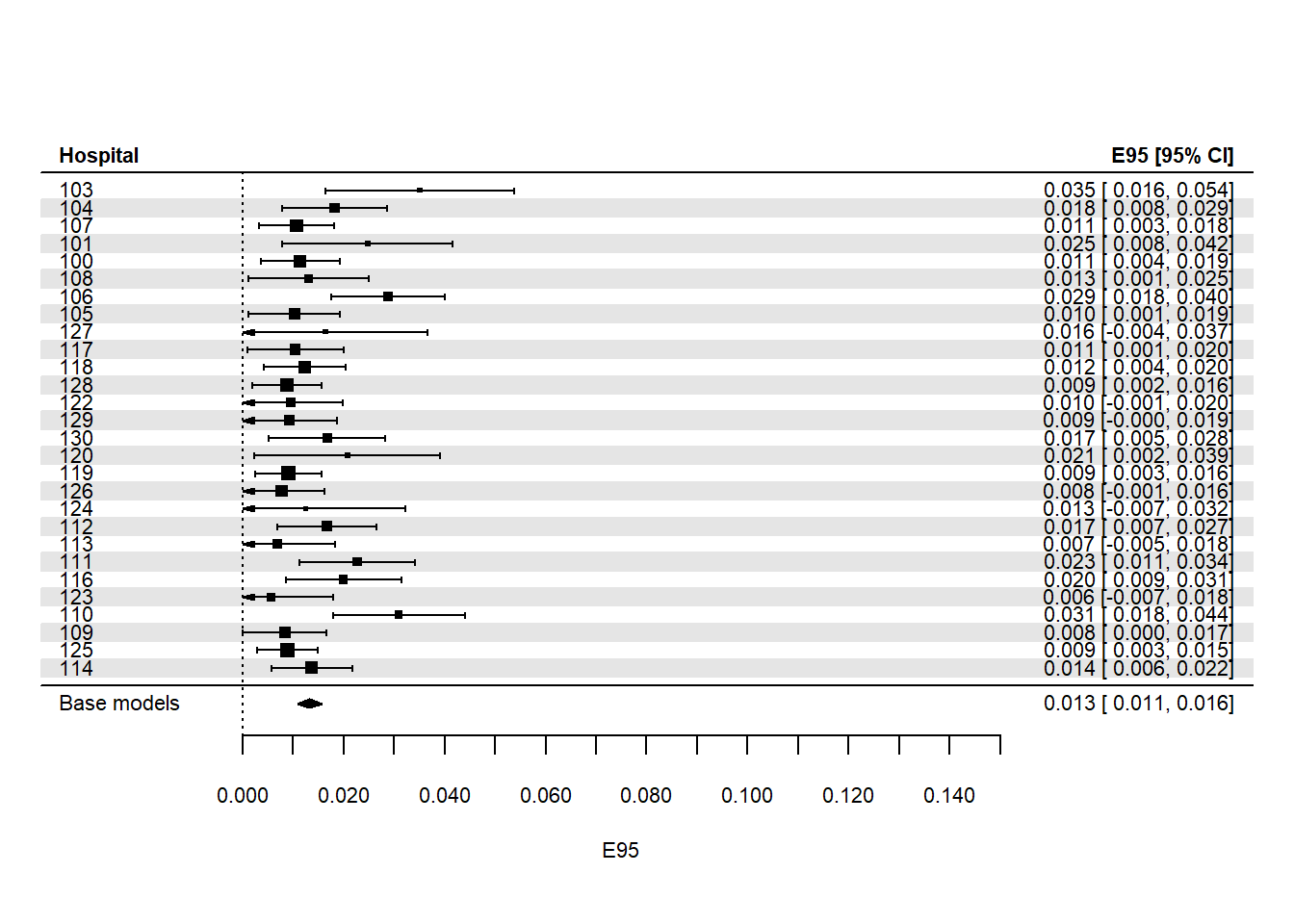

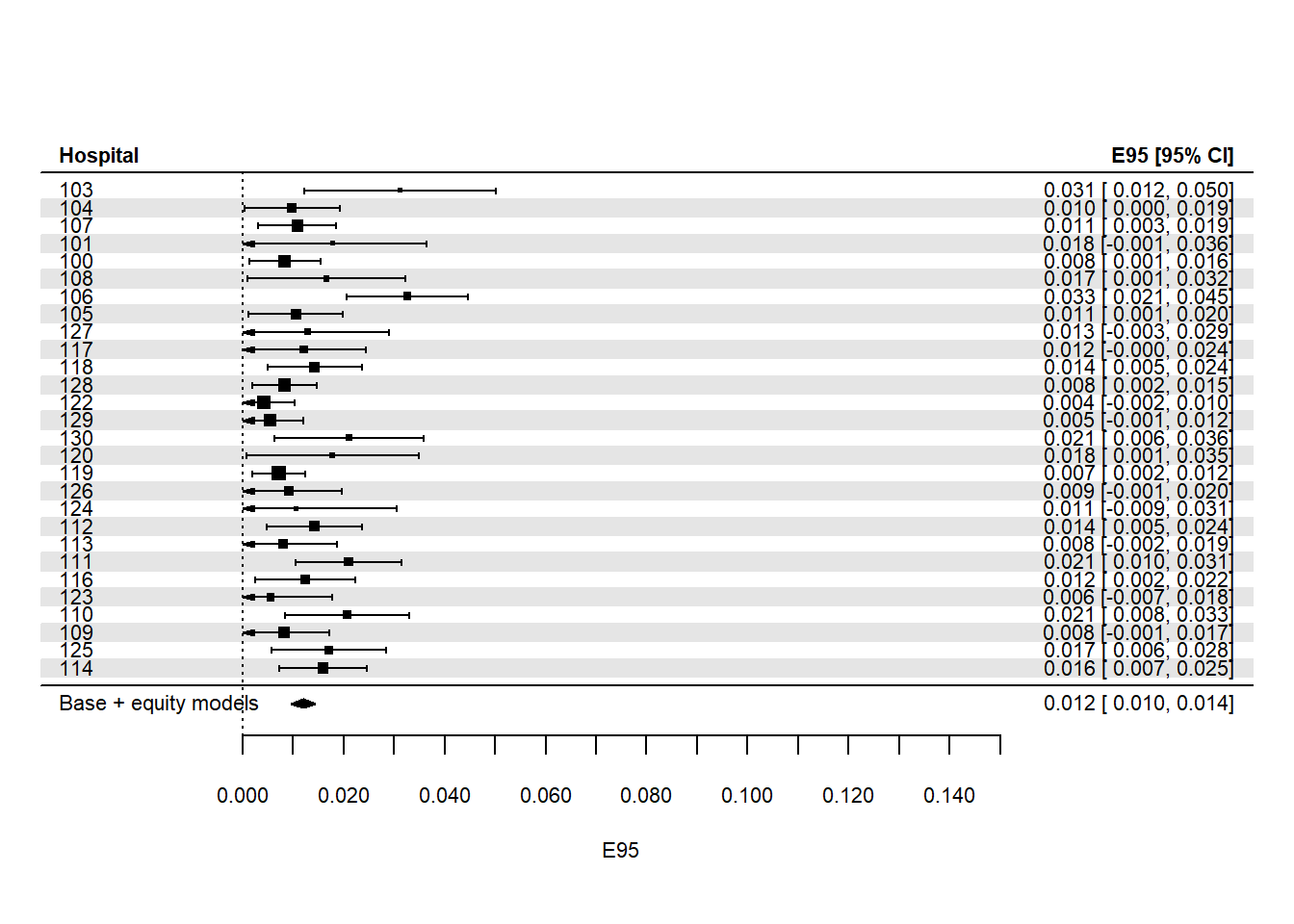


### Figure S3.1: Caterpillar plot of risk-standardized in-hospital mortality rates.

A caterpillar plot of risk-standardized in-hospital mortality rates, with and without equity-related adjustment. The rank order of hospitals in the first panel is maintained in the second panel to visualize changes in rank after equity-related adjustment. The horizontal line represents the crude in-hospital mortality rate across all hospitals.


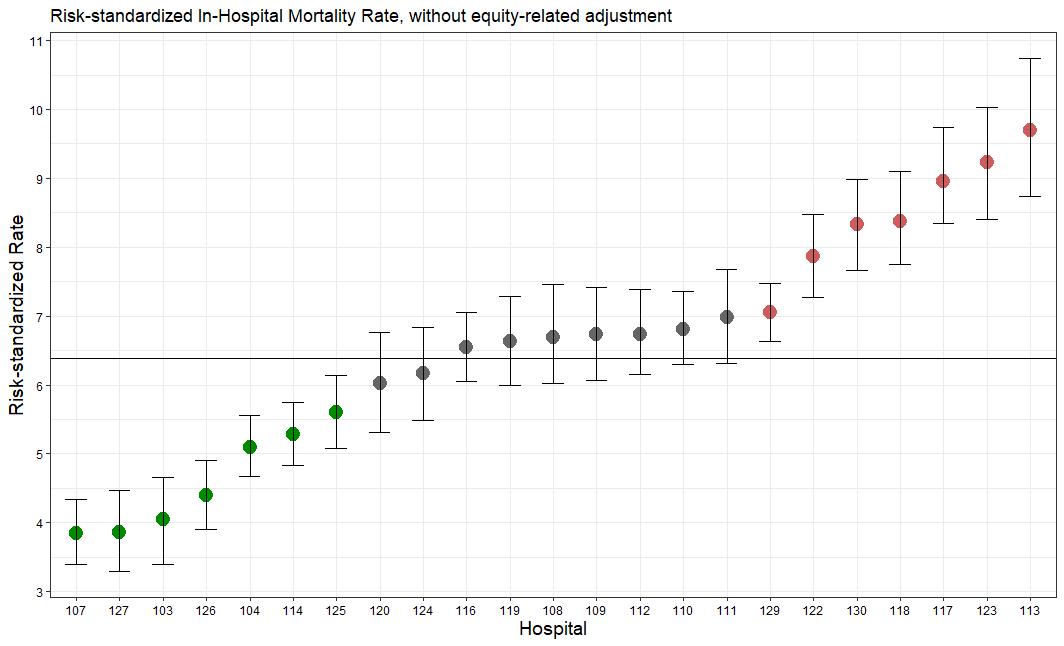


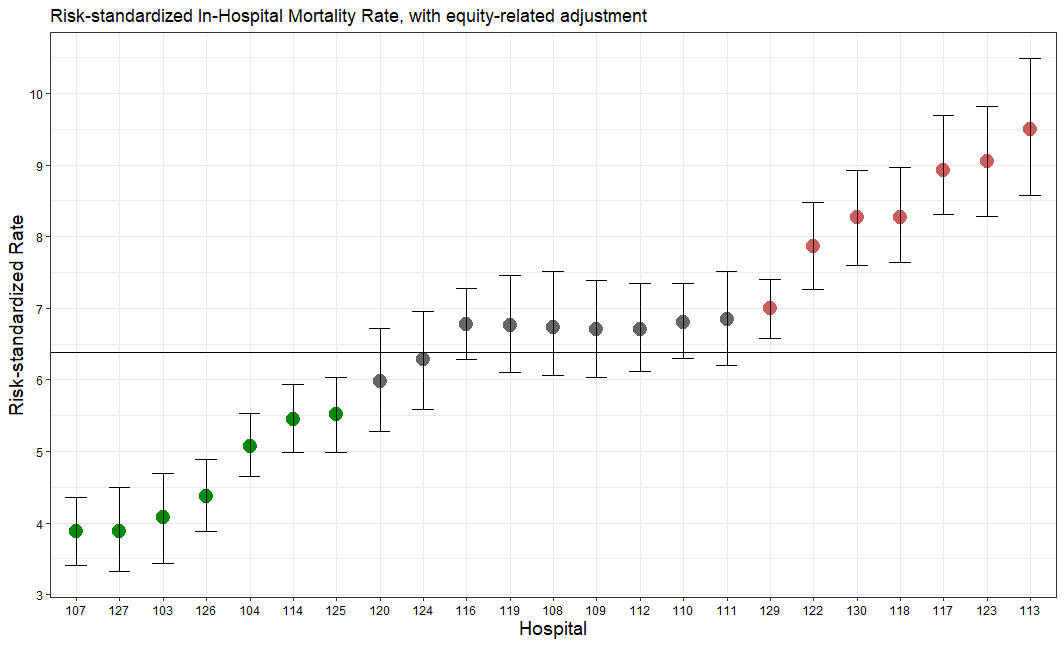


### Figure S3.2: Caterpillar plot of risk-standardized 7-day readmission rates.

A caterpillar plot of risk-standardized 7-day readmission rates, with and without equity-related adjustment. The rank order of hospitals in the first panel is maintained in the second panel to visualize changes in rank after equity-related adjustment. The horizontal line represents the crude 7-day readmission rate across all hospitals.


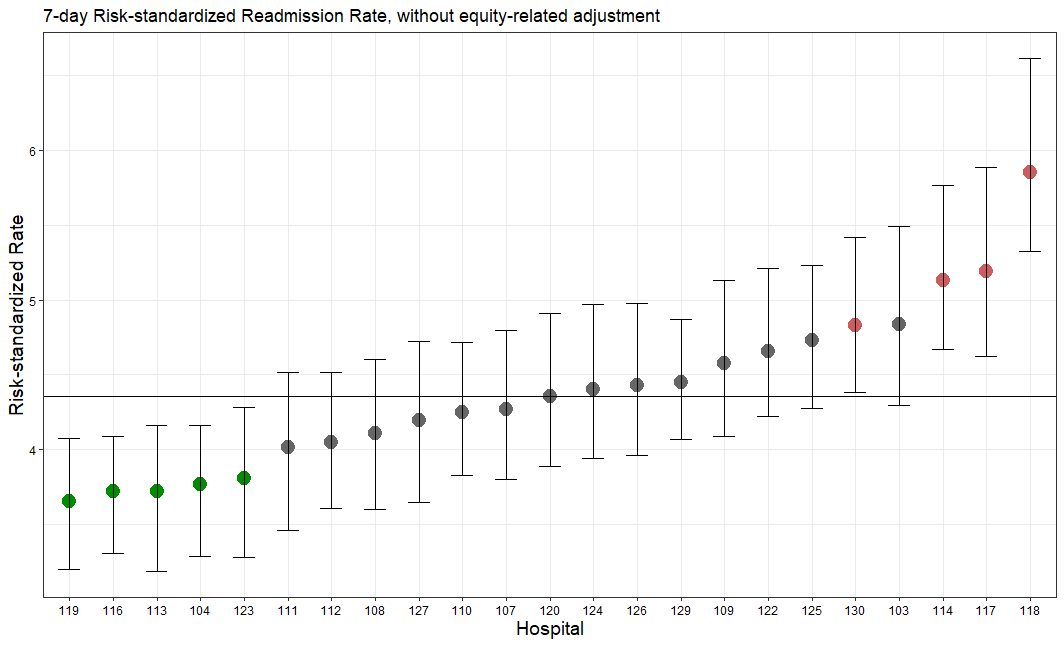

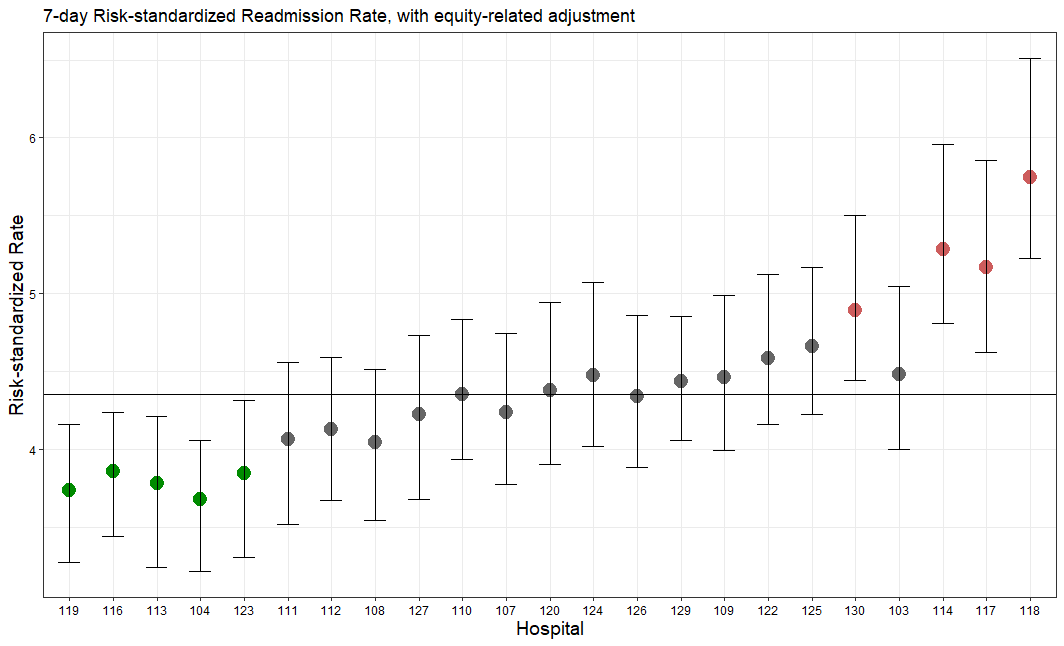


### Figure S4.1: Heatmap to visualize how changes in hospital risk-standardized in-hospital mortality rate are associated with disability, homelessness, and neighborhood marginalization


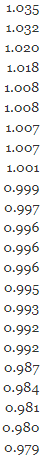
See Figure 3 figure legend.
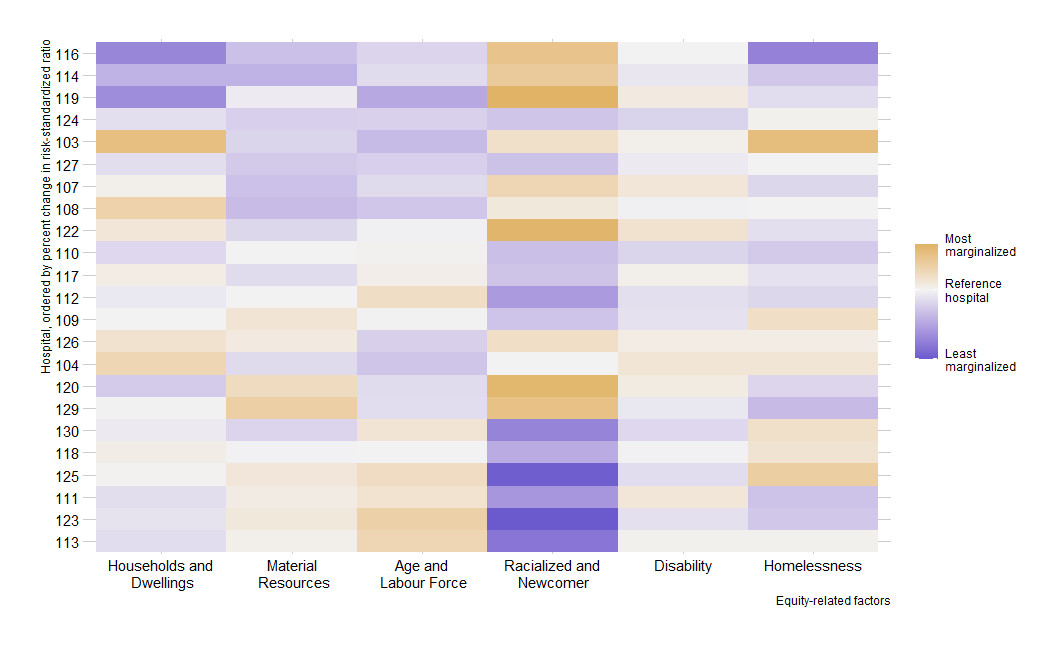


Hospital

Δ RSR

**Decrease** in risk-standardized rate

**Increase** in risk-standardized rate

### Figure S4.2: Heatmap to visualize how changes in hospital risk-standardized 7-day readmission rate are associated with disability, homelessness, and neighborhood marginalization

See Figure 3 figure legend.
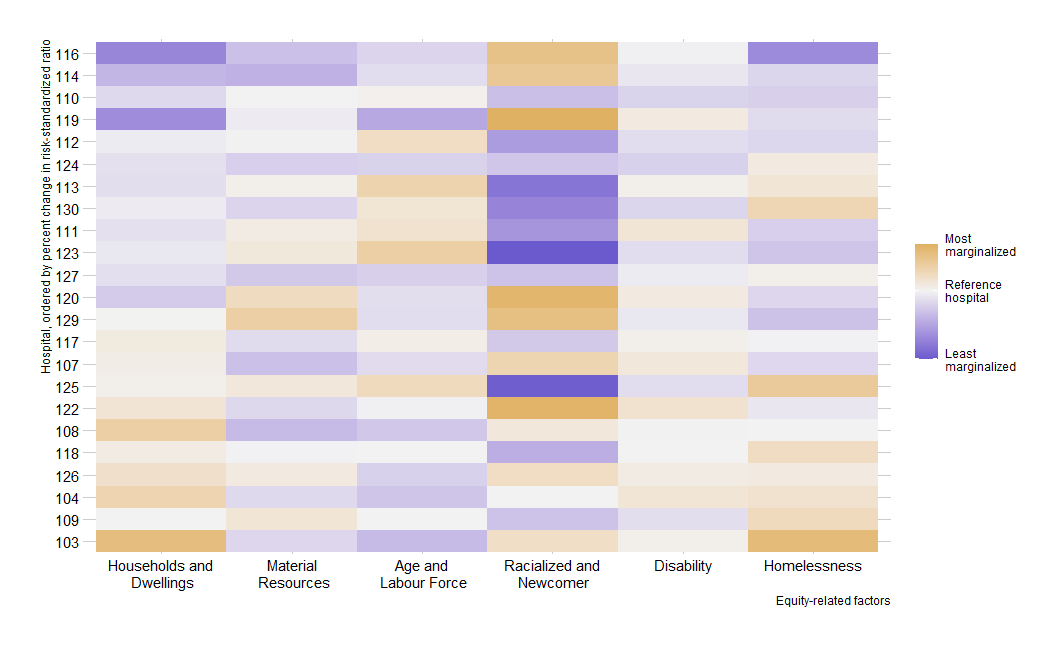


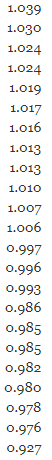


Hospital

Δ RSR

**Decrease** in risk-standardized rate

**Increase** in risk-standardized rate

### Figures S5.1-5.6. Distribution of equity-related factors by hospital

**Figure S5.1: Distribution of Ontario Marginalization Index principal component score for the Housing and Dwellings dimension, by hospital.**

Higher principal component scores indicate a higher degree of marginalization. Data are based on the reporting period. Each number corresponds to a hospital.


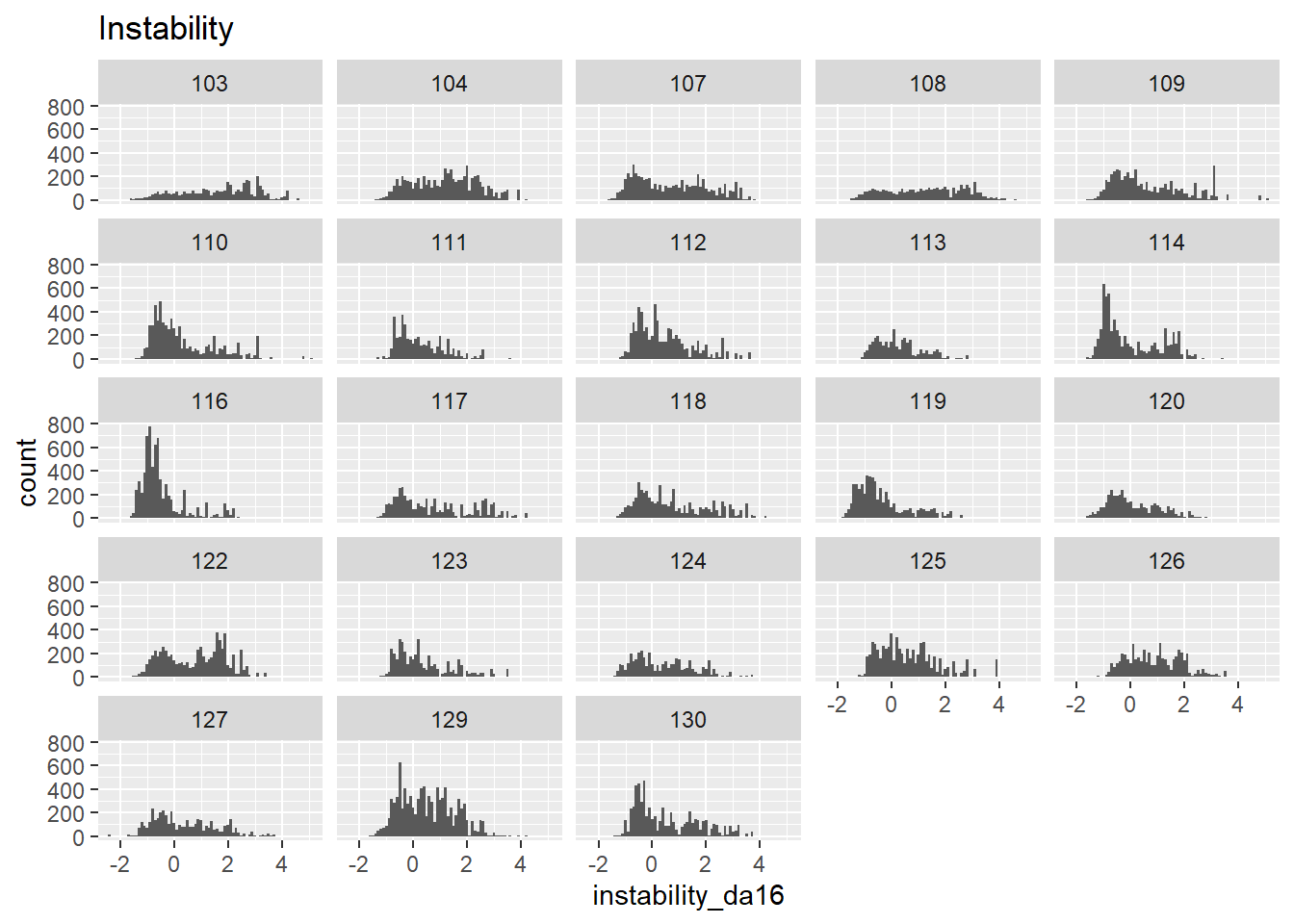


**Figure S5.2: Distribution of Ontario Marginalization Index principal component score for the Racialized and Newcomer dimension, by hospital.**

Higher principal component scores indicate a higher degree of marginalization. Data are based on the reporting period. Each number corresponds to a hospital.


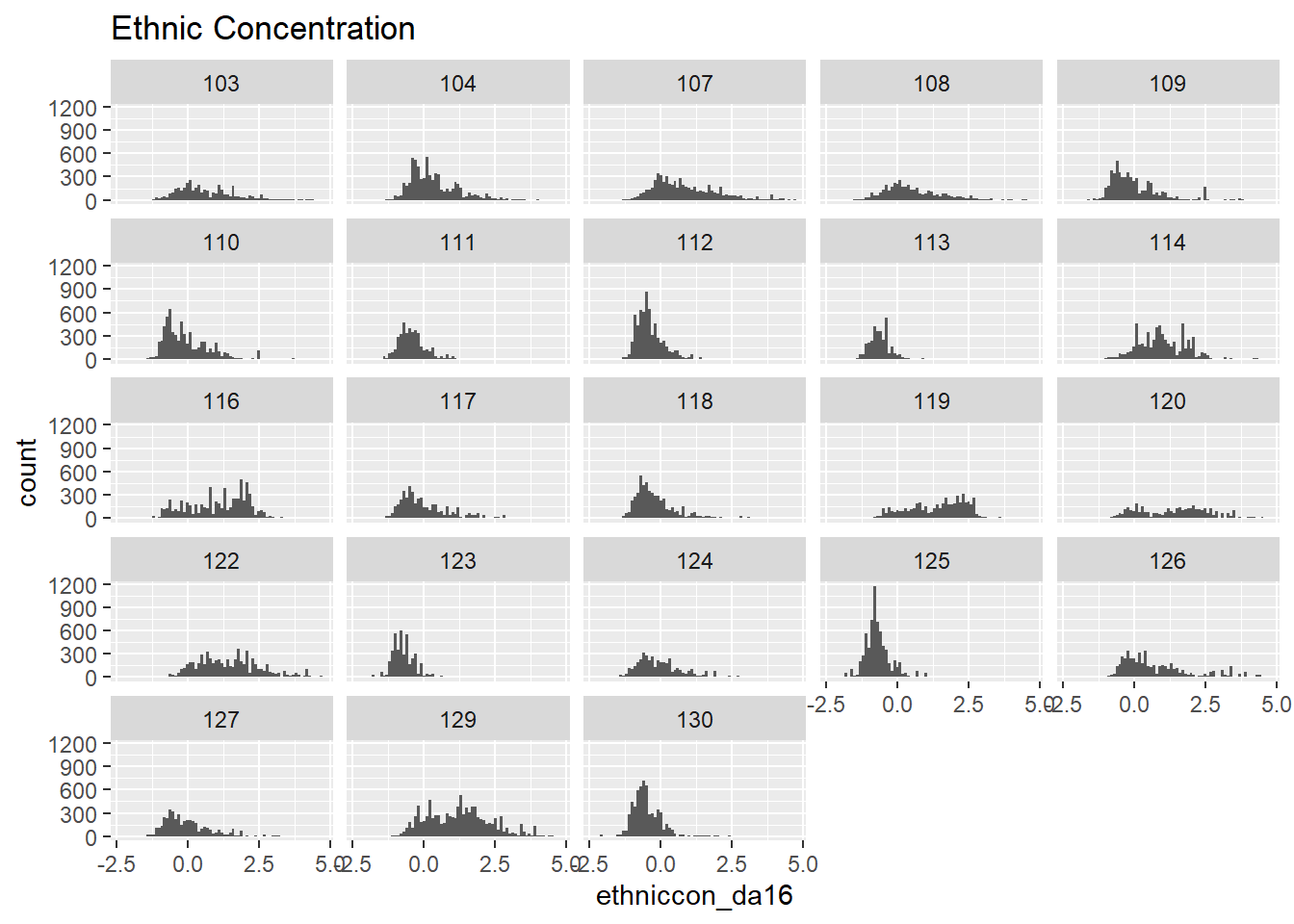


**Figure S5.3: Distribution of Ontario Marginalization Index principal component score for the Age and Labour Force dimension, by hospital.**

Higher principal component scores indicate a higher degree of marginalization. Data are based on the reporting period. Each number corresponds to a hospital.
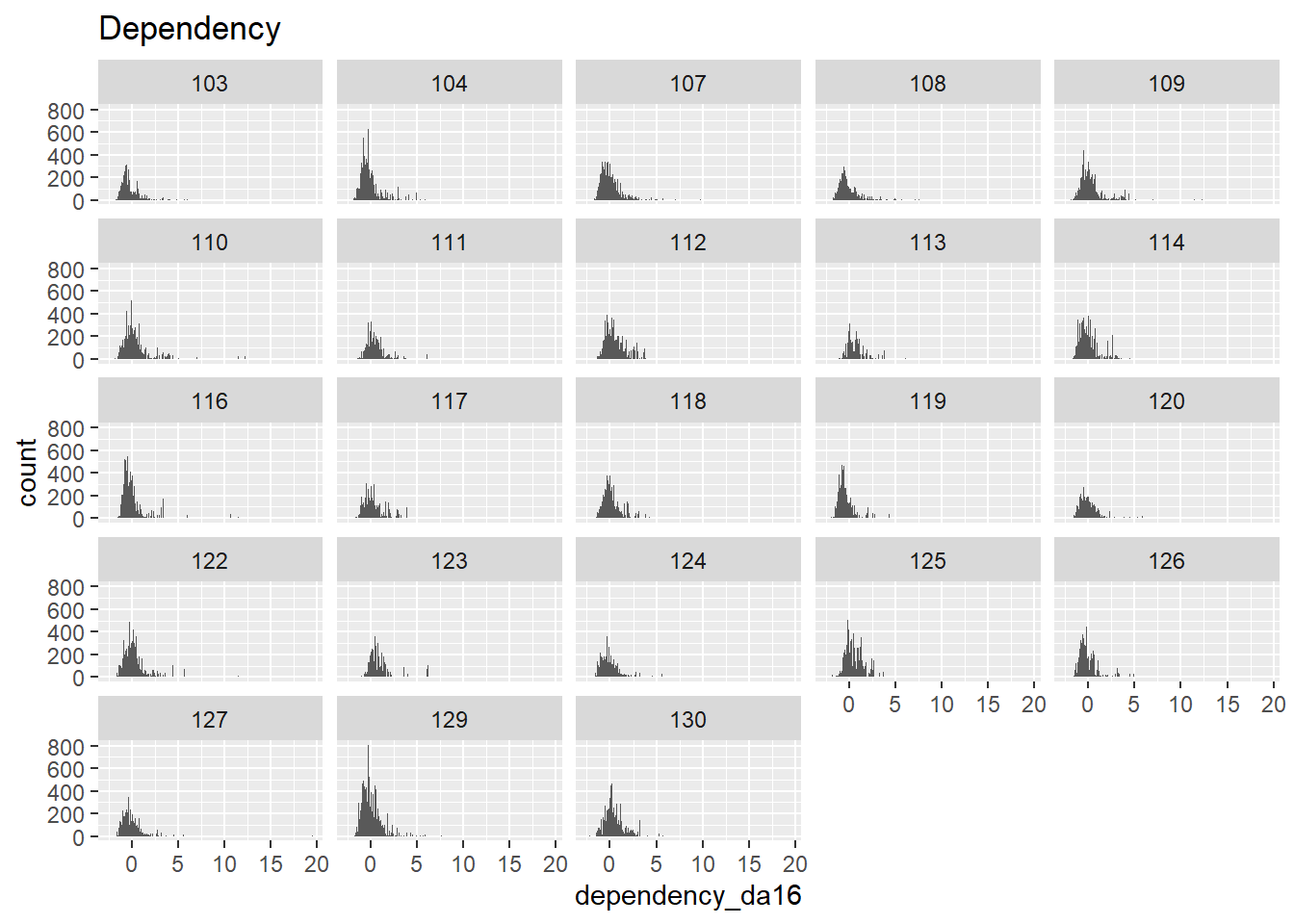


**Figure S5.4: Distribution of Ontario Marginalization Index principal component score for the Material Resources dimension, by hospital.**

Higher principal component scores indicate a higher degree of marginalization. Data are based on the reporting period. Each number corresponds to a hospital.
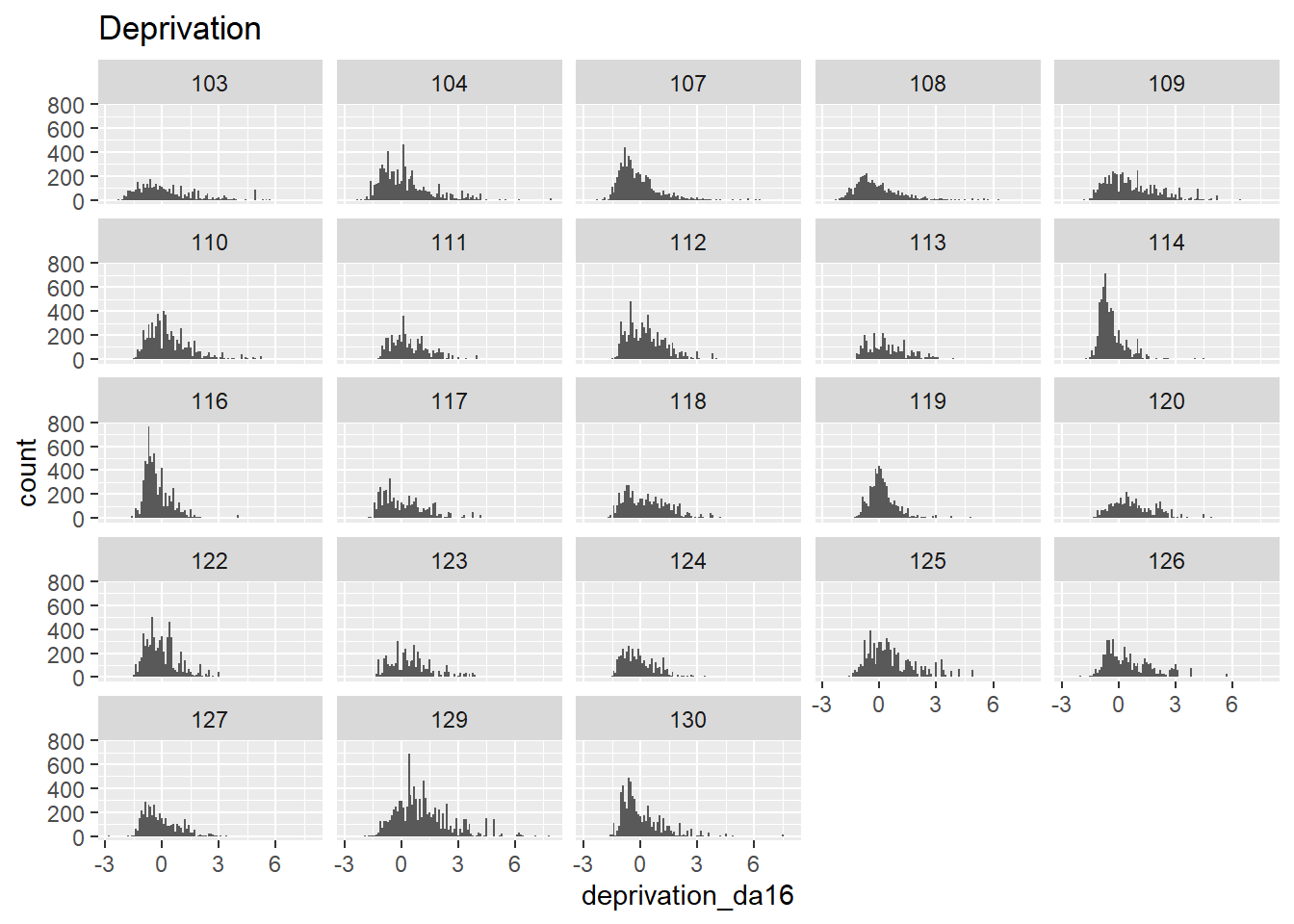


**Figure S5.5: Proportion of admissions for patients experiencing homelessness, by hospital.**

Data are based on the reporting period. Each number corresponds to a hospital.


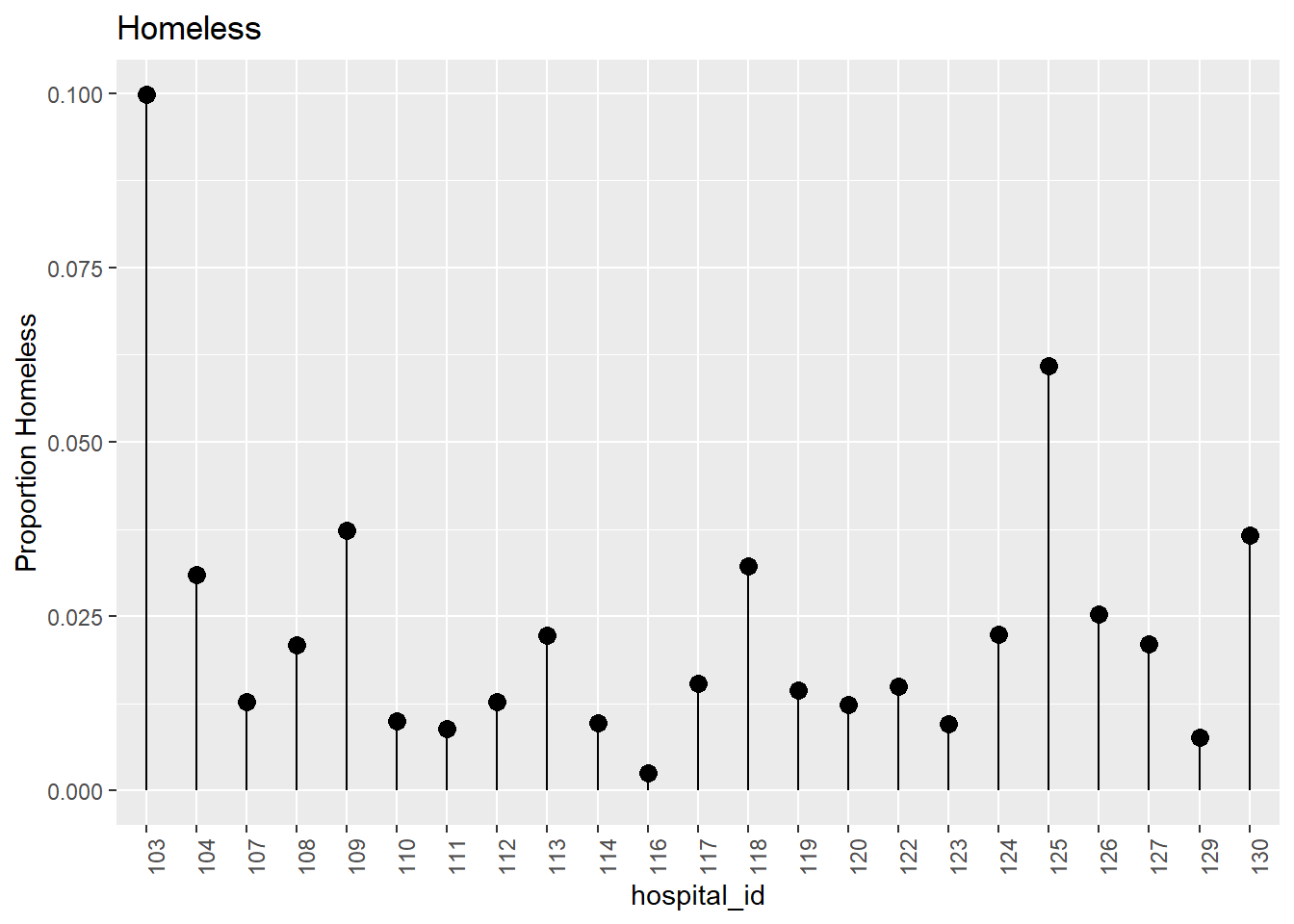


**Figure S5.6: Proportion of admissions for patients with a disability, by hospital.**

Data are based on the reporting period. Each number corresponds to a hospital.


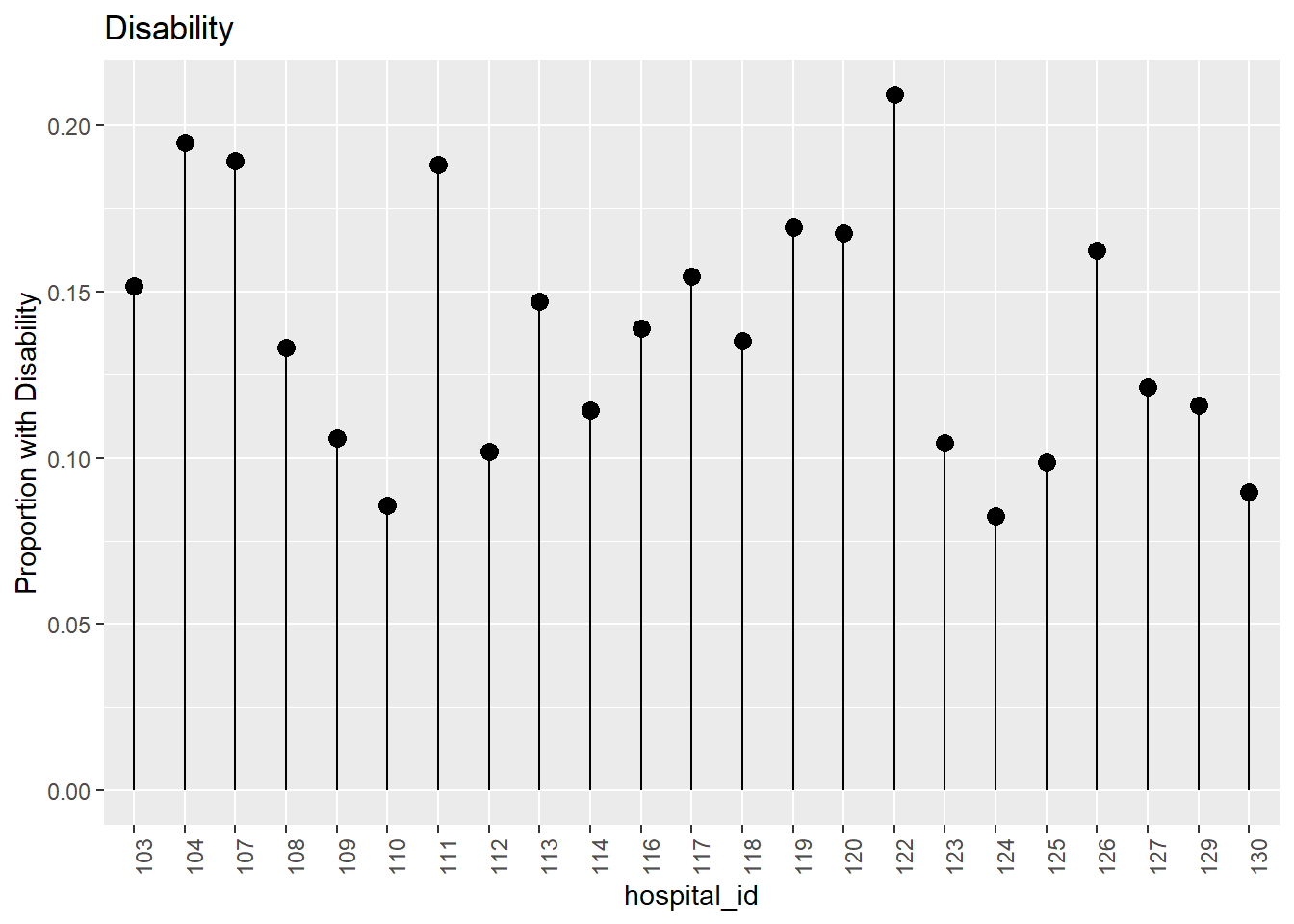


f
